# Forgotten Natural Products: Semisynthetic Development of Blasticidin S As an Antibiotic Lead

**DOI:** 10.1021/acsmedchemlett.3c00527

**Published:** 2024-02-23

**Authors:** Cole Gannett, Kateland Tiller, Anthony J. Briganti, Anne M. Brown, James Weger-Lucarelli, Andrew N. Lowell

**Affiliations:** † Department of Chemistry, Virginia Polytechnic Institute and State University (Virginia Tech), Blacksburg, Virginia 24061, United States; ‡ Center for Emerging, Zoonotic, and Arthropod-borne Pathogens, Virginia Polytechnic Institute and State University (Virginia Tech), Blacksburg, Virginia 24061, United States; § Department of Biomedical Sciences and Pathobiology, Virginia Tech, VA-MD Regional College of Veterinary Medicine, Blacksburg, Virginia 24061, United States; ∥ Department of Biochemistry, Virginia Tech, Blacksburg, Virginia 24061, United States; ⊥ Research and Informatics, Virginia Tech, Blacksburg, Virginia 24061, United States; # Interdisciplinary Program in Genetics, Bioinformatics, and Computational Biology, Virginia Tech, Blacksburg, Virginia 24061, United States; g Faculty of Health Sciences, Virginia Polytechnic Institute and State University (Virginia Tech), Blacksburg, Virginia 24061, United States

**Keywords:** semisynthesis, natural products, antibiotics, antimicrobial resistance, blasticidin S, peptidyl nucleoside, selectivity

## Abstract

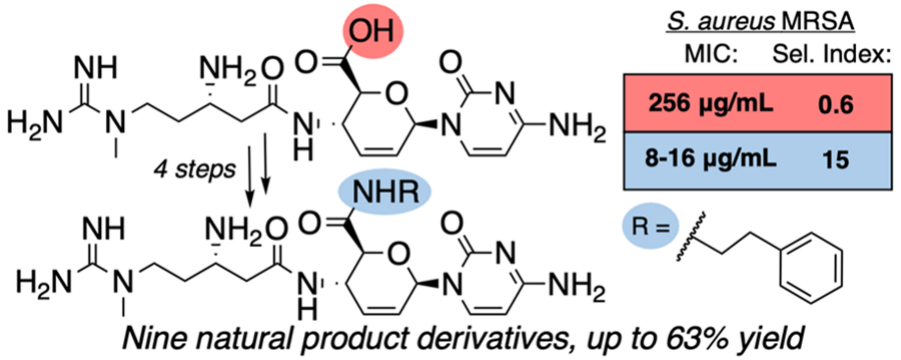

Forgotten natural products offer value as antimicrobial scaffolds, providing diverse mechanisms of action that complement existing antibiotic classes. This study focuses on the derivatization of the cytotoxin blasticidin S, seeking to leverage its unique ribosome inhibition mechanism. Despite its complex zwitterionic properties, a selective protection and amidation strategy enabled the creation of a library of blasticidin S derivatives including the natural product P10. The amides exhibited significantly increased activity against Gram-positive bacteria and enhanced specificity for pathogenic bacteria over human cells. Molecular docking and computational property analysis suggested variable binding poses and indicated a potential correlation between *c*Log*P* values and activity. This work demonstrates how densely functionalized forgotten antimicrobials can be straightforwardly modified, enabling the further development of blasticidin S derivatives as lead compounds for a novel class of antibiotics.

The troubling rise of antimicrobial resistance (AMR) and the imminent threat it poses to public health around the globe necessitate immediate action to prioritize the development of new antibiotics.^1^ The four most widely prescribed antibiotics (amoxicillin, azithromycin, cephalexin, and doxycycline) are all semisynthetic analogues of natural products.^2^ Indeed, natural products have provided the majority of new antibiotics,^3^ but beyond these frequently used and well-studied powerhouse scaffolds, the discovery of natural product antibacterial compounds with new mechanisms of action (i.e., new classes of antibiotics) has drastically slowed.^4^ Known but less-used and under-studied antibacterial natural product architectures hold promise for development into antibiotic leads and offer a critical supplement to ongoing discovery efforts.^5^

The peptidyl nucleoside natural product blasticidin S^6^ (**1**, Figure 1) is hypothesized to trap the ribosome–release factor 1 complex in a pretermination state,^7^ holding promise as an antibacterial with a new mechanism of action. While broadly active against both Gram-positive and Gram-negative bacteria,^8^**1** possesses only moderate activity and is generally cytotoxic,^9^ requiring medicinal chemistry to enhance its potency and selectivity. However, derivatization of forgotten antibiotics, such as blasticidin S, is a major challenge because of their polar (zwitterionic) and acid/base sensitive properties. Toward this goal, we recently synthesized ester derivatives at the C6′ carboxylate of **1**.^8^ Our ester derivatives, synthesized in a single step, increased the antibacterial potency against Gram-positive bacteria and improved selectivity for bacterial versus human cells. However, greater activity and selectivity are required to convert these under-studied scaffolds into antibiotic leads. P10 (**2**), a natural product analogue of blasticidin S,^9^ demonstrated an increased potency, especially against Gram-negative bacteria. The current work focuses on derivatization of the C6′ position to produce various amide derivatives, a synthetic challenge that requires a multistep approach, and use of recent ribosome computational advances to assess potential binding differences.^10^

**Figure 1:**
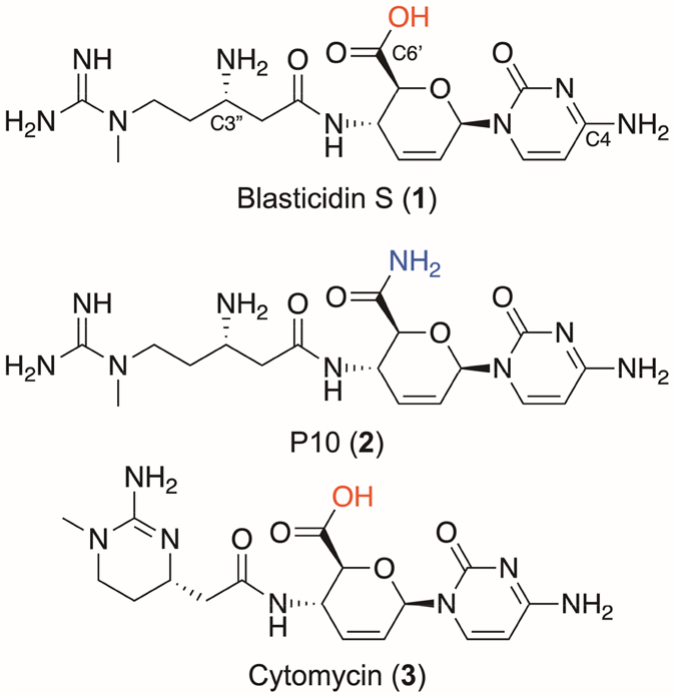
Blasticidin S (**1**), P10 (**2**), and cytomycin (**3**).

While blasticidin S ester derivatives could be generated in one step from **1**, this process was not suitable with amines as nucleophiles due to the base-sensitivity of the β-arginine portion of the molecule. Blasticidin S is known to undergo rapid intramolecular cyclization between the protonated guanidine (p*K*_a_ > 12.5) and the deprotonated (p*K*_a_ = 8.0) primary amine under basic conditions, producing cytomycin (**3**) and liberating ammonia.^11^ Additionally, a strong aqueous acid can hydrolyze the internal amide or the glycosidic bond, compromising activity. Combined, these factors limit the number of available approaches.

To form C6′ amides of blasticidin S, we initially tried peptide coupling reactions under mildly basic aqueous conditions with small alkyl amines. Cytomycin (**3**) was observed as the major product, and decreasing the pH resulted in no reaction. To avoid the formation of **3**, a new route involving protection of the C3″ primary amine was formulated. We hypothesized that this approach would prevent cyclization under basic conditions and enable alternative and more atom economical coupling methods, such as methyl esterification of the acid and subsequent displacement of the ester by an amine to generate the amide. Toward this end, *tert*-butyl carbamate (boc) protected **1** was synthesized but found to be nearly unreactive with diazomethane at the C6′ acid. Similarly, 2,2,2-trichloroethyl carbamate (troc) protected **1** did not react with diazomethane and reacted only sluggishly with thionyl chloride in methanol. This work showed that the C3″ amine could be successfully masked, but that reaction at the C6′ acid of these protected compounds was challenging.

Given previous successes in synthesizing ester derivatives of blasticidin S in high yield,^8^ an optimized route to a protected methyl ester (**5**, Scheme 1) commenced with esterification of **1** using methanol and thionyl chloride. The unpurified methyl ester (**4**, isolated as the trihydrochloride salt) was subsequently boc protected by using boc anhydride and triethylamine in methanol. The protected blasticidin S methyl ester (**5**) was purified using automated reversed-phase flash chromatography and isolated as the monoformic acid salt with a two-step yield of 90%.

**Scheme 1:**
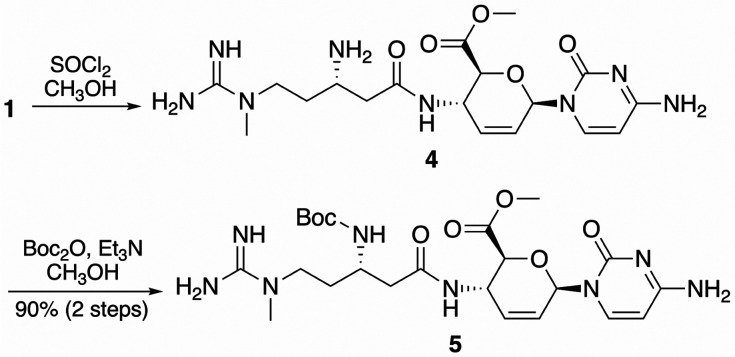
Synthetic Route to Intermediate **5**

Intermediate **5** was exposed to various amines to yield boc-protected amides **6**–**14** (Scheme 2). Notably, carbamate protection of the β-amine was successful in preventing unwanted cyclization (as was observed with **4**) under basic conditions. Commercial solutions of ammonia, methylamine, and ethylamine in methanol were used as the solvent in reactions with **5**, which proceeded to completion at room temperature in a sealed vial to form the corresponding amides (**6**–**8**). Retail dimethyl amine in methanol was also used as solvent; however, the greater steric demands of the dimethyl amine significantly slowed its reaction with **5**, allowing trace amounts of water to compete, hydrolyzing the ester. This process was optimized by the addition of 3 Å molecular sieves and gentle heating (37 °C) in a sealed vial for an extended reaction time, furnishing **10**. Less volatile amines—propylamine, propargylamine, 3-butynylamine, and phenethylamine—were dissolved in methanol and mixed with **5**, providing **9**, **11**, **12**, and **14** in high yields at room temperature in sealed vials over 24–48 h.

**Scheme 2:**
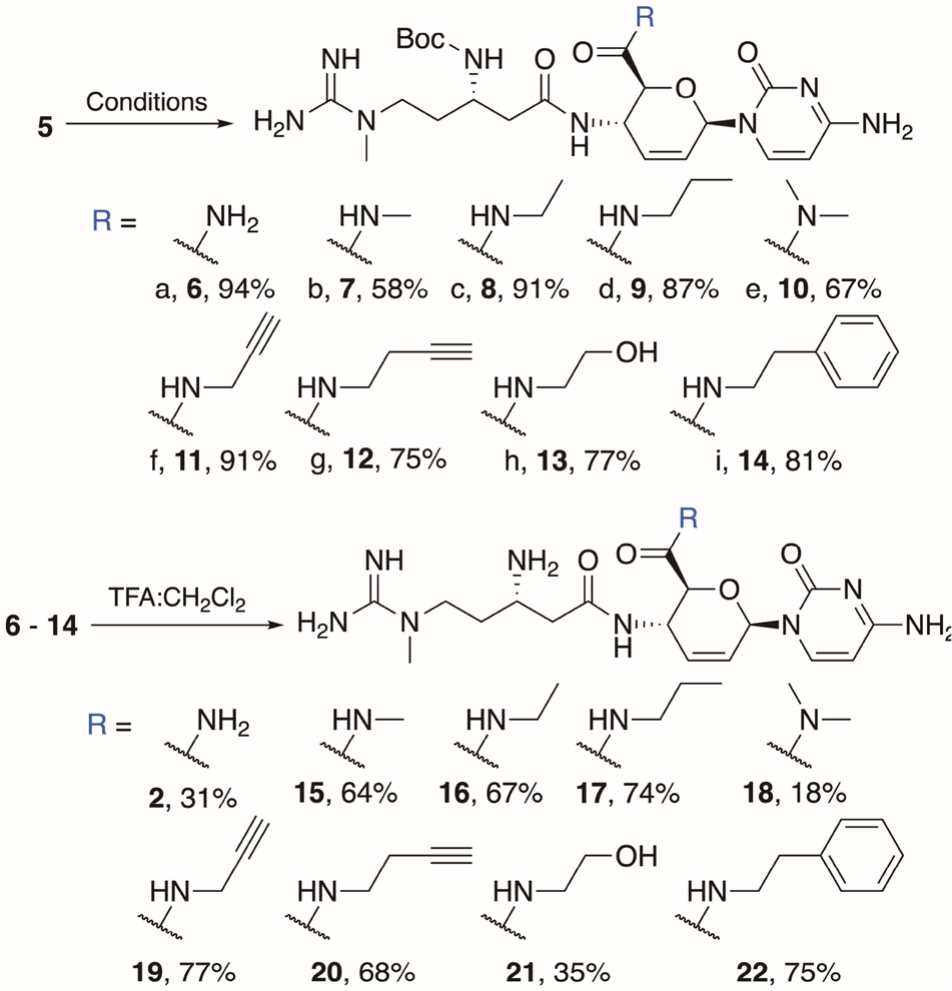
Synthetic Route to Protected Amides **6–14**, **2**, and Derivatives **15–22** (a) 7 M NH_3_ in MeOH, (b) 3 M MeNH_2_ in MeOH, (c) 2 M EtNH_2_ in MeOH, (d) 1:7 *n*PrNH_2_:MeOH, (e) 2 M Me_2_NH in MeOH, 3 Å mol. sieves, 37 °C, (f) 4:1 propargylamine:MeOH, (g) 4:1 3-butynylamine:MeOH, (h) 1:6 ethanolamine:MeOH, (i) 1:6 phenethylamine:MeOH.

We hypothesized that amidation of the methyl ester would be compatible with the free alcohol functionality, selectively producing amides. Treating **5** with a solution of ethanolamine in methanol furnished **13** in a modest yield. Reaction of **5** with hydroxylamine generated the desired hydroxamic acid group at C6′, but additional unwanted reactions occurred. Hydroxylamine also reacted at the C4 and C6 positions via known processes,^12^ producing a mixture of products. This mixture was inseparable using automated flash chromatography, precluding the isolation of a hydroxamic acid derivative. Regardless, this result and the successful isolation of **13** demonstrate that installation of alcohol functionality on the amine chain is indeed compatible with this amidation method.

Automated flash chromatography was used to purify boc-protected amides **6**–**14** and, after deprotection with trifluoroacetic acid (TFA), the corresponding desired salts **2** and **15**–**22**. Typically, polar compounds, such as **2** and **6**-**22**, are purified using semipreparative HPLC.^13^ However, our implementation of medium-pressure reverse phase methods enabled purification of both the protected intermediates (**6**–**14**) and the final amide derivatives (**2**, **15**–**22**) in a straightforward manner, drastically decreasing the time and effort required, despite the highly polar nature of these compounds.

Following successful purification, blasticidin S (**1**), P10 (**2**), and the amide derivatives **15**–**22** were screened for antibacterial activity against various Gram-positive and -negative human pathogens including *Staphylococcus aureus* with the efflux pump NorA knocked out (ΔNorA), *S. aureus*, methicillin-resistant *S. aureus* (MRSA), *Enterococcus faecalis*, vancomycin-resistant *Enterococcus* (VRE), *Klebsiella pneumoniae*, *Pseudomonas aeruginosa*, and *Acinetobacter baumannii*. The minimum inhibitory concentration (MIC) and IC_50_’s are shown in Table 1. Amide derivatization of the C6′ carboxylate of blasticidin S generally increased activity relative to **1** except against the ΔNorA strain of *S. aureus*. The reduced activity against *S. aureus* ΔNorA potentially indicates that cellular entry is dependent on the NorA transporter as has been demonstrated for blasticidin S and P10.^9^ Secondary alkyl amides up to four atoms long (**15**–**17**, **19**–**21**) all showed similar inhibition of Gram-positive bacterial growth, while dialkyl tertiary amide **18** displayed a slightly reduced level of potency, indicating that the H-bond donor of the amide may enhance activity. The best performing derivative against the Gram-positive strains was phenethyl amide **22**, increasing potency against the high-priority pathogens VRE and MRSA by 8-fold and 16 to 32-fold, respectively, compared to **1** and showing similar or better activity than **2**. The longest alkane (**17**) and alkynyl chains (**19** and **20**) showed a slight increase in activity relative to shorter secondary amides, suggesting that some combination of electronics and length are responsible for the superiority of **22**. As an isostere of **22**, the phenethyl ester reported in our previous study^8^ had the same activity against *E. faecalis* and VRE, but **22** has 4- to 8-fold better activity against *S. aureus* and MRSA. This result highlights that aromatic character at this position imparts better inhibition, which might be further enhanced by altering the electronics of the ring.

**Table 1:**
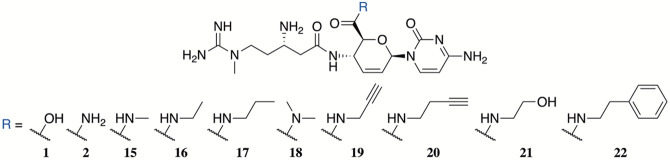
MIC (μg/mL) and IC_50_ (μg/mL ± S.D.) Values for Blasticidin S (**1**), P10 (**2**), and Derivatives **15**–**22**

	**MIC and IC**_**50**_**(μg/mL)**									
**Organism**	**1**	**2**	**15**	**16**	**17**	**18**	**19**	**20**	**21**	**22**
*S. aureus* ΔNorA	**>256**	**64**–**128**	**128**	**128**	**64**–**128**	**128**	**64**–**128**	**64**	**256**	**128**
	140 ± 8.7	14.0 ± 0.3	21.1 ± 0.4	19.6 ± 1.8	15.8 ± 1.1	18.3 ± 1.4	16.2 ± 0.9	15.2 ± 2.1	35.5 ± 2.5	19.3 ± 0.2
*S. aureus*	**>256**	**32**	**32**	**32**	**16**–**32**	**64**	**32**	**16**	**64**	**16**
	33.2 ± 0.8	4.7 ± 1.0	5.9 ± 1.2	5.3 ± 1.5	3.9 ± 0.1	7.0 ± 1.5	3.6 ± 0.3	2.5 ± 0.2	8.1 ± 0.7	3.8 ± 0.2
MRSA	**256**	**32**	**32**–**64**	**32**	**16**–**32**	**32**	**16**–**32**	**32**	**32**–**64**	**8**–**16**
	59.7 ± 7.8	7.0 ± 1.0	13.3 ± 3.3	14.9 ± 1.6	7.6 ± 0.8	12.8 ± 1.9	6.9 ± 1.2	7.2 ± 2.3	11.8 ± 0.2	4.0 ± 0.3
*E. faecalis*	**32**–**64**	**16**–**32**	**16**–**32**	**16**–**32**	**16**–**32**	**16**–**32**^a^	**16**–**32**	**16**–**32**	**32**	**8**
	26.3 ± 13	5.1 ± 4.3	13.2 ± 3.6	9.6 ± 4.0	9.3 ± 1.7	17.0	7.6 ± 1.5	11.1 ± 4.1	22.3 ± 4.0	6.1 ± 2.6
VRE	**256**	**64**	**32**	**64**	**64**	**64**–**128**	**64**	**64**	**128**	**32**
	58.0 ± 9.4	15.0 ± 8.6	13.5 ± 3.6	20.2 ± 8.1	16.8 ± 3.1	27.7 ± 11	21.1 ± 4.8	22.1 ± 12	15.3 ± 3.3	11.1 ± 0.8
*K. pneumoniae*	**128**	**32**	**64**	**256**	**128**–**256**	**256**	**128**	**128**	**64**	**256**
	42.3 ± 3.5	7.6 ± 1.3	26.4 ± 4.1	60.1 ± 6.2	44.9 ± 3.2	60.1 ± 4.7	40.4 ± 6.2	30.7 ± 5.3	19.7 ± 0.5	36.9 ± 4.7
*P.aeruginosa*	**256**	**64**	**64**–**128**	**64**–**128**	**64**	**64**–**128**	**64**	**64**	**128**	**64**
	61.0 ± 6.0	14.6 ± 1.5	20.2 ± 3.4	21.8 ± 2.7	19.3 ± 1.6	18.8 ± 2.6	16.1 ± 0.9	20.2 ± 1.3	34.1 ± 0.3	16.5 ± 2.1
*A. baumannii*	**>256**	**16**	**64**	**128**	**>256**	**256**	**128**	**128**	**64**–**128**	**>256**
	104 ± 25	4.5 ± 0.4	18.3 ± 5.3	35.7 ± 11	39.3 ± 1.9	46.4 ± 7.0	21.1 ± 3.9	24.3 ± 3.2	21.2 ± 0.6	183 ± 19

a Result is based on one biological replicate (*n* = 2); standard deviation was not determined.

Inhibition of the Gram-negative strains by amide derivatives was generally stronger than or similar to that of blasticidin S (**1**). Activity against wild-type *P. aeruginosa* was improved up to 4-fold for the secondary amides and greater than 4-fold against *A. baumannii*, but no derivative showed consistent top activity against all three strains. Methyl amide **15** and ethanol amide **21** showed the broadest activity. The previously discovered natural product P10 (**2**) remained the most active compound against all Gram-negative bacteria assayed.

In our previous SAR with esters of blasticidin S,^8^ activity typically tended to decrease with increasing chain length of the ester alkyl group. In this study, chain length did not appear to have a pronounced effect on inhibition among aliphatic amides. However, the longest straight chain used was four atoms (**20**) for amides versus six atoms for the esters. Further, the longer amides (**19** and **20**) terminated in an alkyne, introducing the potential for π interactions that the longest esters lacked. Chain length did not seem to have a prominent effect among the saturated amides (compare **15** to **17**), but potential π interactions with the ribosome for the longer chain alkynyl amides (**19** and **20**) slightly increased their relative activity.

Cytotoxicity assays (Table 2) of our amide derivatives against human cells (MRC-5 lung fibroblast cells) were used to determine CC_50_ for each compound. The natural product primary amide, P10 (**2**), was found to be the most toxic while the amides **15**–**22** showed a slight to moderate improvement in CC_50_ versus blasticidin S (**1**). The IC_50_’s for each compound were compared to the CC_50_’s to obtain a selectivity index (SI, Table 2) for each compound/pathogen pair. Blasticidin S (**1**) did not have any SI’s greater than 1, while **2** only had three SI’s greater than 1 and none greater than 2. The greatest SI for the amide derivatives was 26 for **16** and 26 for **20** both versus *S. aureus*. The average selectivity index for each new amide derivative was between 3.3 and 9.4, significantly higher than **1** (0.4) and **2** (0.8) and the previous ester derivatives.^8^ The ethyl and phenethyl amides (**16 & 22**) had the most SI’s greater than 10 (*S. aureus*, *E faecalis* and *S. aureus*, MRSA, respectively). Selectivity indices greater than 10, and especially greater than 25, approach the viable range for a lead compound suitable for clinical development.

**Table 2:** CC_50_ (μg/mL) and SI (CC_50_/IC_50_) Values for Blasticidin S (**1**), P10 (**2**), and Derivatives **15**–**22**

	**1**	**2**	**15**	**16**	**17**	**18**	**19**	**20**	**21**	**22**
**CC**_**50**_**(μg/mL) for human cells**										
MRC-5 (lung fibroblast)^a^	20.1	5.9	52.8	137	61.1	63.9	33.1	65.9	58.1	58.4
**Selectivity index (CC**_**50**_**/IC**_**50**_**)for bacteria**										
*S. aureus* ΔNorA	0.1	0.4	2.5	7.0	3.9	3.5	2.0	4.3	1.6	3.0
*S. aureus*	0.6	1.3	8.9	26	16	9.1	9.2	26	7.1	15
MRSA	0.3	0.8	4.0	9.2	8.1	5.0	4.8	9.1	4.9	15
*E. faecalis*	0.8	1.2	4.0	14	6.6	3.7^b^	4.4	5.9	2.6	9.6
VRE	0.3	0.4	3.9	6.8	3.6	2.3	1.6	3.0	3.8	5.2
*K. pneumoniae*	0.5	0.8	2.0	2.3	1.4	1.1	0.8	2.2	3.0	1.6
*P. aeruginosa*	0.3	0.4	2.6	6.3	3.2	3.4	2.1	3.3	1.7	3.5
*A. baumannii*	0.2	1.3	2.9	3.8	1.6	1.4	1.6	2.7	2.7	0.3

a Results are based on one biological replicate (*n* = 3); standard deviation was not determined, but results are representative of multiple trials.

b Result is based on one biological replicate.

To explore how molecular features influence binding, molecular docking studies were performed with blasticidin S (**1**), P10 (**2**), and the amide derivatives (**15**–**22**), comparing their binding poses to the cocrystal structure of blasticidin S with the *T. thermophilus* ribosome and *E. coli* release factor 1 (PDB 6B4V).^7^ Bearing in mind the charge state (Figure 2A), comparison of the crystal structure (2**B**) to the lowest energy poses for **1** (**2C**), **2** (**2D**), and the most active amide **22** (**2E**) show conserved interactions with the underlying ribosome pharmacophore model. (Figures S42–S52 show the poses of the other amides). The critical interactions are π stacking or cation-π interactions (orange ring on the left side of the panels) and binding to a highly negative pocket (represented by two red spheres in the upper half of the panels).

**Figure 2:**
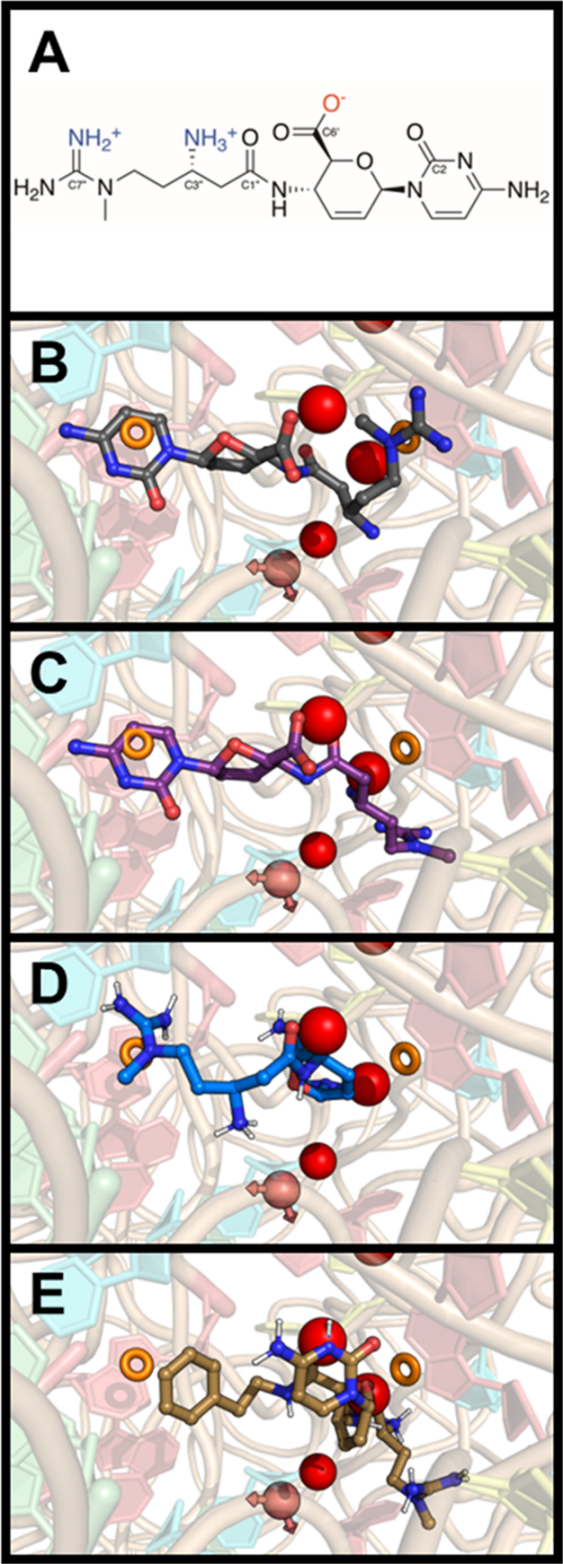
(A) Numbering scheme and charge state for blasticidin S used for docking studies. (B) Crystal blasticidin S and the pharmacophore model. Pharmacophore features of the ribosome near the binding site are represented by red spheres (negative charge), orange rings (π stacking or cation-π interactions), and pink spheres (hydrogen bond acceptor with the arrows pointing in the direction of the lone pairs); (C–E) Lowest energy pose and pharmacophore model for **1** (purple sticks), **2** (blue sticks), and **22** (gold sticks), respectively.

Redocking of the parent molecule, blasticidin S (**1**), shows that the C1″ carbonyl oxygen occupies a highly negative pocket of the pharmacophore model (Figure 2C, red spheres), causing unfavorable, repulsive interactions. Transformation of the C6′ carboxylic acid of **1** enables the resulting amide derivatives (such as **22** in Figure 2E) to create a more favorable interaction by reorienting the C6′ nitrogen, a relatively electropositive and H-bond-donating group, into this negative pocket. This reorientation suggests why the tertiary amide (**18**) is less active; its steric inability to properly orient the nitrogen of the C6′ amide or its lack of H-bond interaction with this pocket is detrimental compared to that of the secondary amides. The C6′ amide of **2** (Figure 2D) also occupies this pocket in a fashion similar to that of the substituted amide derivatives. However, the lowest energy orientation of **2** reverses the orientation of the cytosine and guanidine groups. Besides this shared electrostatic interaction, all derivatives maintain important intercalation interactions (left orange circle in Figure 2C–E) between the C-74 and A-76 nucleotides to displace C-75.

Building on the pharmacophore model of Figure 2, the specific docking interactions of **1**, **2**, and **22** show close proximity to several specific nucleotides (Figure 3). The phenethyl amide (**22**) is particularly adept at maintaining essential interactions and enhancing new ones; it is uniquely able to maintain two different aromatic interactions in the binding site (Figure 3G). The phenyl ring of **22** maintains the aforementioned intercalation between C-74 and A-76 through π interactions, displacing C-75 and allowing the cytosine base to exchange its usual Watson–Crick base pair interactions with G-2251 for H-bonds with A-2439 and A-2602, bases that are usually associated with the guanidine tail of **1** (Figure 3A). The guanidine tail of **22** is then able to extend into a new region of the P-site, coordinating with H-bonds to the RNA phosphate backbone. The lowest energy pose of P10 (**2**, Figure 3D) flips its orientation in the pharmacophore model relative to blasticidin S (**1**, Figure 3A) so that the guanidine tail of **2** intercalates between C-74 and A-76 instead of the cytosine as in **1**. This pose allows H-bond interactions between the C3″ amine and C-2064. Additionally, this reorientation places the cytosine base and sugar in a new region of the P-site, interacting with A-2439 and A-2602. Adopting a different binding mode does not necessarily mean a different mechanism of action is at work; other poses of **2** (Figure 3E) match that of **1**, highlighting the dynamic nature of ligand binding to the ribosome.

**Figure 3:**
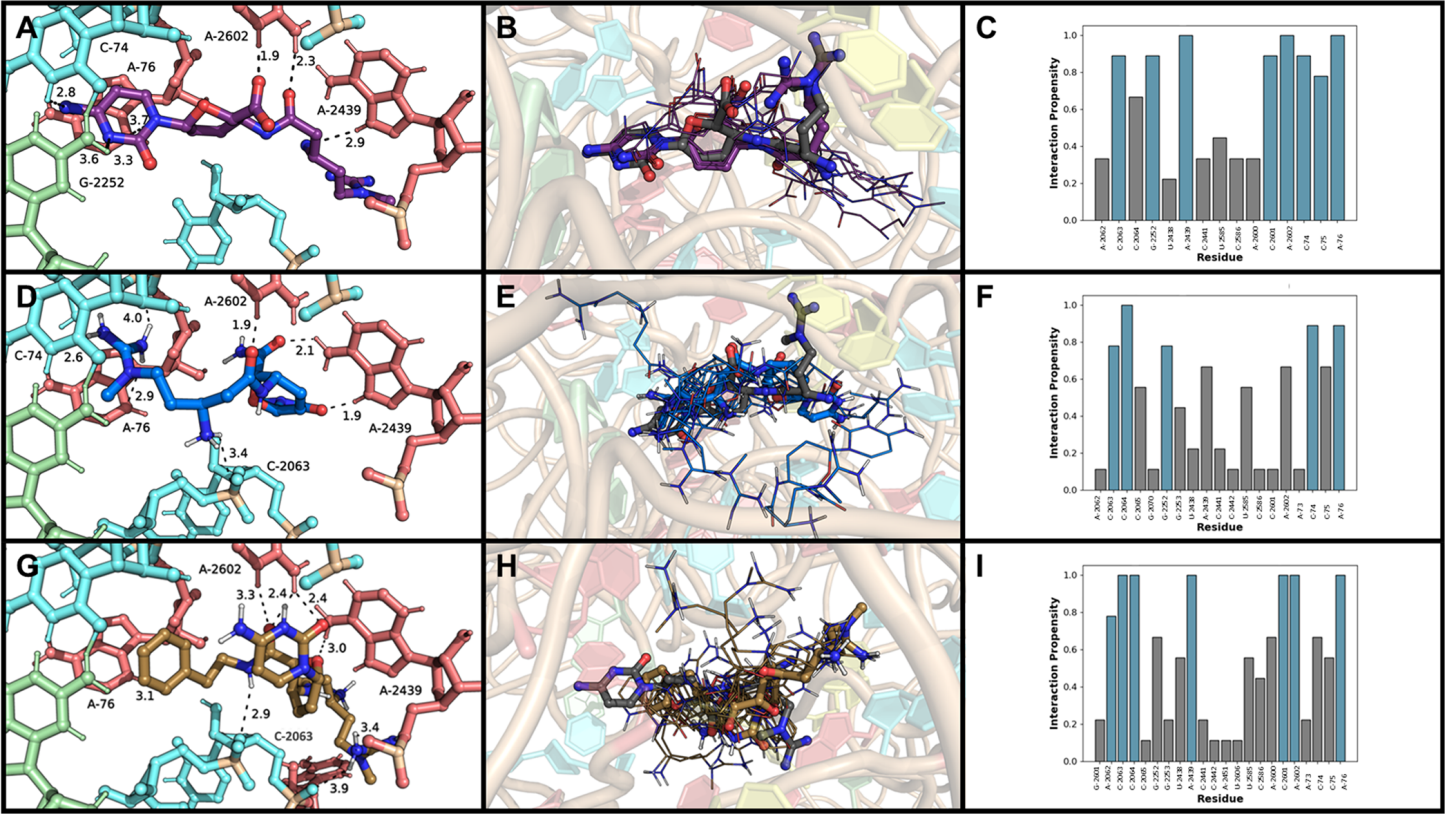
Interaction map and proximities for lowest energy pose of (A) **1**, (D) **2**, and (C) **22**. Crystal structure of **1** (gray sticks) overlaid with ten lowest energy poses for (B) **1** (purple), (E) **2** (blue), and (H) **22** (gold); the lowest energy pose (matching Figure 2C–E) is shown as sticks, and the other nine are shown as lines. Fingerprint analyses for (C) **1**, (F) **2**, and (I) **22**; interactions greater than 70% are highlighted in blue.

Consideration of all docked poses of **1**, **2**, and **22** (Figure 3B, E, and H, respectively) also enables fingerprint analyses (Figure 3C, F, I), which identifies high-frequency nucleotide-compound interactions in support of binding. Among the multiple binding modes of each compound, persistent intercalative interactions are present between C-74/A-76, and the cytosine of **1**, guanidine of **2**, and phenethyl group of **22**, respectively. The differing orientations also support new high frequency interactions between the C3″ amine of **2** with the backbone phosphate of C-2063 and from the C6′ amide nitrogen and guanidine of **22** to the backbone phosphates of C-2063 and C-2064.

The ability of **2** and the amide derivatives to replace the repulsive interactions between the C1″ carbonyl oxygen of **1** and the negative pocket in which it is oriented with more attractive electrostatic interactions between the electropositive nitrogen of the C6′ amide and this pocket may contribute to some of the observed increase in antibacterial activity.

Besides the computational results suggesting that favorable electrostatic interactions and alternative binding poses of **2** and the amides (**15**–**22**) are responsible for their increase in activity, bacterial cellular uptake rates may be the cause of the observed differences. Previous studies assessing **1** and **2** with cell-free protein synthesis (*E. coli* ribosome) assays showed similar inhibition by both compounds,^9^ a result that suggests the increased activity of **2** and **15**–**22** could be attributable to membrane permeability. Indeed, property data for **1**, **2**, and **15**–**22** (computed using the SwissADME web tool)^15^ show a potential correlation between computed Silicos-IT log *P* values^16^ and MIC for the Gram-positive bacteria excluding *S. aureus* NorA knockout (see Table S3 and Figures S49–S52). Further intensive studies, such as cell-free protein synthesis assays coupled with in-depth computational properties and docking studies, are needed to discriminate between the binding and permeability effects of blasticidin S derivatives.

These results, along with those from the previous study,^8^ suggest that while derivatizing the C6′ position enhances antibacterial potency and selectivity, further work is needed to achieve an increase in selectivity (SI > 100) sufficient for use as a treatment. The more selective nucleoside antibacterial natural product amicetin has been shown to bind in an overlapping binding site with blasticidin S,^14^ indicating that guided derivatization of other locations may result in greater gains in selectivity. Thus, continuing SAR studies and the development of other positions on the molecule are critical for continued advancement of blasticidin S derivatives as potential antibiotics.

In conclusion, a semisynthetic route to blasticidin S derivatives was developed by using an esterification and protection strategy that enabled, after deprotection, diversification of this chemically challenging compound into a library of amides. This strategy enabled purification of these highly polar natural product derivatives using reverse phase automated flash chromatography. The amide derivatives have increased antibacterial activity against Gram positive bacteria and showed minor improvement against Gram negative bacteria versus blasticidin S. The cytotoxicity of the amide derivatives was lower than that of the blasticidin S, resulting in compounds with much better selectivity indices versus blasticidin S and the previous ester derivatives,^8^ approaching the levels required for clinical development. Docking studies suggested that the enhanced activity of the amide derivatives of blasticidin S is due to more favorable electrostatic interactions with the ribosome, some of which may be due to alternative binding conformations. Future investigations will seek to disentangle binding enhancement from activity increases due to other cellular factors. This study indicates that C6′ derivatization of compounds considered to be cytotoxins can increase their bacterial inhibition and selectivity for pathogenic bacteria versus human cells; however, greater selectivity gains are needed and likely achievable through derivatization at other sites on the molecule.

## Supporting Information Available

Experimental details, assay information, NMR spectra, representative HPLC traces, computational methods, and property data (PDF)

## Abbreviations

boc: *tert*-butyl carbamate
troc: 2,2,2-trichloroethyl carbamate
ΔNorA: *Staphylococcus aureus* mutant lacking the NorA efflux transporter
MRSA: methicillin resistant *Staphylococcus aureus*
VRE: vancomycin resistant *Enterococcus* species
TFA: trifluoroacetic acid
