# Supplementary material for: Forgotten Natural Products: Semisynthetic Development of Blasticidin S As an Antibiotic Lead
Source: ACS Med Chem Lett. 2024 Feb 23;15(3):362–8. doi: 10.1021/acsmedchemlett.3c00527 (PMC10945559; doi:10.1021/acsmedchemlett.3c00527)
Supplement: Supplementary file 1 — ml3c00527_si_001.pdf [file ml3c00527_si_001.pdf]

Supporting Information

Forgotten natural products: Semisynthetic development of blasticidin S as an antibiotic lead

Cole Gannett,[a,b] Kateland Tiller,[b,c] Anthony J. Briganti,[d] Anne M. Brown,[b,d,e,f] James Weger-Lucarelli,[b,c], Andrew N. Lowell[a,b,g,*]

[a] Department of Chemistry, Virginia Polytechnic Institute and State University (Virginia Tech), Blacksburg, VA 24061, United States

[b] Center for Emerging, Zoonotic, and Arthropod-borne Pathogens, Virginia Polytechnic Institute and State University (Virginia Tech), Blacksburg, VA 24061, United States

[c] Department of Biomedical Sciences and Pathobiology, Virginia Tech, VA-MD Regional College of Veterinary Medicine, Blacksburg, VA 24061, United States

[d] Department of Biochemistry, Virginia Tech, Blacksburg, Virginia 24061, United States

[e] Research and Informatics, Virginia Tech, Blacksburg, Virginia 24061, United States

[f] Interdisciplinary Program in Genetics, Bioinformatics, and Computational Biology, Virginia Tech, Blacksburg, Virginia 24061, United States

[g] Faculty of Health Sciences, Virginia Polytechnic Institute and State University (Virginia Tech), Blacksburg, VA 24061, United States

[*]Corresponding author; email for A.N.L., alowell@vt.edu

Table of Contents

General Experimental..S3

Synthetic Procedures and Characterization for Compounds **5-22**...S4

^{1}H and ^{13}C NMR Spectra for Compounds **5-22**..S16

Representative HPLC traces for compounds **1**, **2**, and **22** S34

Antibacterial Assay Procedure...S36

Cytotoxicity Assay Procedure..S37

Molecular Docking Information...S38

Property Data for Compounds **1**, **2**, **15-22**...S42

References.. S44

General Experimental

Blasticidin S hydrochloride was purchased from Diagnocine. Unless otherwise specified, all reagents, solvents, and media components were purchased commercially and used as received from Sigma Aldrich, Fisher Scientific, or Oakwood Chemical. Deionized water was obtained from the house deionized water system. All synthetic reactions were stirred with a magnetic stir bar under a nitrogen atmosphere unless otherwise stated. No unexpected or unusually high safety hazards were encountered. All compounds are >95% pure by HPLC.

Specific rotations were obtained on a Jasco P-2000 polarimeter. ^{1}H NMR spectra were recorded on a Bruker Avance II 500 MHz spectrometer, Agilent U4-DD2 400 MHz spectrometer, or Bruker Avance III 600 MHz spectrometer. Chemical shifts are reported in parts per million (ppm) using the solvent resonance as an internal standard (CD_3OD 3.31 ppm). Data are reported as follows: chemical shift, multiplicity (s=singlet, d=doublet, t=triplet, q=quartet, quint=quintet, sext=sextet, m=multiplet), coupling constants (Hz), and number of protons. Proton decoupled ^{13}C NMR were recorded on a Bruker Avance II 500 MHz (^{13}C 125 MHz) spectrometer or an Agilent U4-DD2 400 MHz (^{13}C 100 MHz) spectrometer. Chemical shifts are reported in ppm using the solvent resonance as an internal standard (CD_3OD 49.0 ppm). NMR signals attributed to the counteranions (formate, trifluoroacetate) for salts of **5**-**22** are not tabulated but included in yield calculations. High resolution mass spectra were obtained on an Agilent Technologies 6220 TOF LC/MS, a Waters Synapt Q-TOF G2, or Thermo Exploris 120 HESI Orbitrap MS in the Department of Chemistry or the VT-Mass Spectrometry Incubator at the Virginia Polytechnic Institute and State University. Automated flash chromatography was performed using a Biotage Selekt system using water with 0.1% (v/v) formic acid as solvent A and acetonitrile (CH_3CN) with 0.1% (v/v) formic acid as solvent B. Commercial C18 cartridges were purchased from Biotage. Analytical HPLC for purity assessment was performed using a Shimadzu Prominence system: CBM-20A communications bus module, LC-20AD pumps, SIL-20A HT auto sampler,

and SDP-M20A diode array detector with a Phenomenex Luna 5 μm C18(2) 100 Angstrom, 250 x 4.6 mm LC column (P/No. 00G-4252-E0).

<u>Synthetic Procedures and Characterization for Compounds 5-22</u>

Methyl (2*S*,3*S*,6*R*)-6-(4-amino-2-oxopyrimidin-1(2*H*)-yl)-3-((*S*)-3-((*tert*-butoxycarbonyl)amino)-5-(1-methylguanidino)pentanamido)-3,6-dihydro-2*H*-pyran-2-carboxylate (5). Blasticidin S hydrochloride (502.0 mg, 1.092 mmol) was suspended in anhydrous MeOH (65.0 mL) and cooled in an ice bath (0 °C) with stirring. Thionyl chloride (2.40 mL, 33.1 mmol) was added slowly, and the suspension became a solution, which was then warmed to rt. After 18 h, the solution was concentrated under a stream of nitrogen to yield a white glassy solid. This residue was dissolved in anhydrous MeOH (20 mL) and concentrated to yield an amorphous white solid. Dissolution and concentration were repeated and extended drying yielded blasticidin S methyl ester trihydrochloride (**4**).

Blasticidin S methyl ester trihydrochloride (**4**) was dissolved in anhydrous MeOH (12 mL) and the solution cooled in an ice bath (0 °C). Boc anhydride (0.33 mL, 1.4 mmol) was added slowly, followed immediately by Et_3N (0.35 mL, 2.5 mmol). The solution was warmed to rt and, after 18 h, concentrated under a stream of nitrogen and then vacuum to yield an amorphous white solid. The residue was dissolved in a minimal amount of deionized water and purified using automated flash chromatography (C18, 30 g, 0-50% B) to yield **5** as the mono-formic acid salt (572.1 mg, 90%) as a white amorphous solid: $[\alpha]_D^{24}$ = +35 (*c* 1.2, CH_3OH); ^{1}H NMR (500 MHz, CD_3OD) δ 7.51 (d, *J* = 7.5 Hz, 1H), 6.54 – 6.57 (m, 1H), 6.1 (d, *J* = 10.4 Hz, 1H), 5.94 (d, *J* = 7.5 Hz, 1H), 5.88 (d, *J* = 10.4 Hz, 1H), 4.82 – 4.76 (m, 1H), 4.32 (d, *J* = 8.7, 1H), 3.90 – 3.83 (m, 1H), 3.72 (s, 3H), 3.51 – 3.33 (m, 2H), 3.04 (s, 3H), 2.46 (dd, *J* = 14.3, 5.8 Hz, 1H), 2.38 (dd, *J* = 14.4, 7.4 Hz, 1H), 1.84 – 1.95 (m, 1H), 1.74 (dtd, *J* = 14.1, 9.4, 5.0 Hz, 1H), 1.45 (s, 9H); ^{13}C NMR (125 MHz, CD_3OD) δ 172.9, 170.5, 167.7, 167.4, 158.3, 158.0,

157.8, 143.3, 134.0, 127.7, 97.0, 80.6, 80.5, 77.3, 53.3, 47.2, 46.1, 42.3, 36.7, 32.5, 28.8; HRMS (ESI) calcd for $C_{23}H_{37}N_8O_7$ $[M+H]^+$ 537.2780, found 537.2797.

***tert*-Butyl ((*S*)-1-(((2*S*,3*S*,6*R*)-6-(4-amino-2-oxopyrimidin-1(2*H*)-yl)-2-carbamoyl-3,6-dihydro-2*H*-pyran-3-yl)amino)-5-(1-methylguanidino)-1-oxopentan-3-yl)carbamate (6).** The mono-formic acid salt of **5** (76.1 mg, 0.131 mmol) was dissolved in 7 M ammonia in MeOH (1.5 mL) and stirred in a sealed vial. After 18 h, the reaction was concentrated under a stream of nitrogen and then vacuum to yield a white solid, which was dissolved in a minimal amount of deionized water and purified using automated flash chromatography (C18, 30 g, 0-50% B) to yield the mono-formic acid salt of **6** (69.4 mg, 94%) as an amorphous white solid: $[\alpha]_D^{24}$ = +61 (*c* 1.4, CH_3OH); ^1H NMR (500 MHz, CD_3OD) δ 7.57 (d, *J* = 7.5 Hz, 1H), 6.59 (dt, *J* = 3.4, 1.8 Hz, 1H), 6.07 (dt, *J* = 10.2, 2.0 Hz, 1H), 5.96 (d, *J* = 7.5 Hz, 1H), 5.83 (ddd, *J* = 10.2, 2.5, 1.5 Hz, 1H), 4.79 (dq, *J* = 9.4, 2.6 Hz, 1H), 4.22 (d, *J* = 9.4 Hz, 1H), 3.78 - 3.88 (m, 1H), 3.41 – 3.52 (m, 1 H), 3.26 – 3.36 (m, 1H), 3.03 (s, 3H), 2.50 (dd, *J* = 13.6, 5.0 Hz, 1H), 2.30 (dd, *J* = 13.6, 8.6 Hz, 1H), 1.89 – 1.99 (m, 1H), 1.66 – 1.77 (m, 1H), 1.45 (s, 9H); ^{13}C NMR (125 MHz, CD_3OD) δ 173.0, 172.7, 167.8, 158.28, 158.25, 157.9, 143.2, 135.0, 127.2, 97.2, 81.1, 80.5, 77.6, 47.7, 46.6, 43.1, 36.6, 32.0, 28.8, 25.3; HRMS (ESI) calcd for $C_{22}H_{36}N_9O_6$ $[M+H]^+$ 522.2783, found 522.2790.

***tert*-Butyl ((*S*)-1-(((2*S*,3*S*,6*R*)-6-(4-amino-2-oxopyrimidin-1(2*H*)-yl)-2-(methylcarbamoyl)-3,6-dihydro-2*H*-pyran-3-yl)amino)-5-(1-methylguanidino)-1-oxopentan-3-yl)carbamate (7).** The mono-formic acid salt of **5** (76.4 mg, 0.131 mmol) was dissolved in 2 M methylamine in MeOH (1.5 mL) and stirred in a sealed vial. After 18 h, the reaction was concentrated under a stream of nitrogen and then vacuum to yield a white solid, which was dissolved in a minimal amount of deionized water and purified using automated flash chromatography (C18, 30 g, 0-50% B) to yield the mono-formic acid salt of **7** (44.2 mg, 58%) as an amorphous white solid: $[\alpha]_D^{23}$ = +48° (*c* 2.2, CH_3OH); ^1H NMR (500 MHz, CD_3OD) δ 7.55 (d, *J* = 7.5 Hz, 1H), 6.57 (dt,

J = 3.5, 1.9 Hz, 1H), 6.07 (dt, J = 10.2, 2.1 Hz, 1H), 5.95 (d, J = 7.5 Hz, 1H), 5.83 (ddd, J = 10.2, 2.6, 1.5 Hz, 1H), 4.73 (dq, J = 9.3, 2.6 Hz, 1H), 4.20 (d, J = 9.3 Hz, 1H), 3.80 – 3.88 (m, 1H), 3.53 – 3.42 (m, 1H), 3.31 – 3.49 (m, 1H), 3.04 (s, 3H), 2.71 (s, 3H), 2.48 (dd, J = 13.9, 5.1 Hz, 1H), 2.33 (dd, J = 13.8, 8.0 Hz, 1H), 1.91 – 2.01 (m, 1H), 1.76 (dtd, J = 14.3, 9.8, 4.8 Hz, 1H), 1.45 (s, 9H); ^{13}C NMR (125 MHz, CD_3OD) δ 172.6, 170.9, 167.7, 158.3, 158.1, 157.9, 143.3, 135.0, 127.2, 97.2, 81.1, 80.5, 77.9, 49.9, 47.5, 46.8, 42.8, 36.6, 32.1, 28.8, 26.3; HRMS (ESI) calcd for $C_{23}H_{38}N_9O_6$ $[M+H]^+$ 536.2940, found 536.2936.

***tert*-Butyl ((*S*)-1-(((2*S*,3*S*,6*R*)-6-(4-amino-2-oxopyrimidin-1(2*H*)-yl)-2-(ethylcarbamoyl)-3,6-dihydro-2*H*-pyran-3-yl)amino)-5-(1-methylguanidino)-1-oxopentan-3-yl)carbamate (8).** The mono-formic acid salt of **5** (77.7 mg, 0.133 mmol) was dissolved in 2 M ethylamine in MeOH (1.5 mL) and stirred in a sealed vial. After 18 h, the reaction was concentrated under a stream of nitrogen and then vacuum to yield a clear solid, which was dissolved in a minimal amount of deionized water and purified using automated flash chromatography (C18, 30g, 0-50% B) to yield the mono-formic acid salt of **8** (72.4 mg, 91%) as an amorphous white solid: $[\alpha]_D^{24}$ = +47° (*c* 2.2, CH_3OH); ^{1}H NMR (400 MHz, CD_3OD) δ 7.56 (d, J = 7.4 Hz, 1H), 6.57 (dt, J = 3.4, 1.9 Hz, 1H), 6.07 (dt, J = 10.2, 2.1 Hz, 1H), 5.94 (d, J = 7.5 Hz, 1H), 5.83 (ddd, J = 10.3, 2.6, 1.5 Hz, 1H), 4.74 (dq, J = 9.3, 2.5 Hz, 1H), 4.19 (d, J = 9.2 Hz, 1H), 3.91 – 3.78 (m, 1H), 3.53 – 3.40 (m, 1H), 3.39 – 3.33 (m, 1H), 3.19 (q, J = 7.2 Hz, 2H), 3.04 (s, 3H), 2.47 (dd, J = 14.0, 5.1 Hz, 1H), 2.34 (dd, J = 13.9, 7.9 Hz, 1H), 2.02 – 1.88 (m, 1H), 1.76 (dtd, J = 14.4, 9.7, 4.9 Hz, 1H), 1.45 (s, 9H), 1.10 (t, J = 7.2 Hz, 3H); ^{13}C NMR (125 MHz, CD_3OD) δ 172.6, 170.1, 167.6, 166.5, 158.3, 158.0, 157.9, 143.4, 135.0, 127.2, 97.1, 81.2, 80.5, 78.0, 47.4, 46.7, 42.7, 36.6, 35.1, 32.2, 28.8, 14.8; HRMS (ESI) calcd for $C_{24}H_{40}N_9O_6$ $[M+H]^+$ 550.3096, found 550.3108.

***tert*-Butyl ((*S*)-1-(((2*S*,3*S*,6*R*)-6-(4-amino-2-oxopyrimidin-1(2*H*)-yl)-2-(propylcarbamoyl)-3,6-dihydro-2*H*-pyran-3-yl)amino)-5-(1-methylguanidino)-1-oxopentan-3-yl)carbamate (9).** The mono-formic acid salt of **5** (75.0 mg, 0.129 mmol) was dissolved in a 1:7 ratio (v/v) solution

of propylamine to anhydrous MeOH (1.5 mL) and stirred for 18 h in a sealed vial. The reaction was concentrated under a stream of nitrogen and then vacuum to yield a white solid, which was dissolved in a minimal amount of deionized water and purified using automated flash chromatography (C18, 30 g, 0-50% B) to yield the mono-formic acid salt of **9** (68.3 mg, 87%) as an amorphous clear to white solid: $[\alpha]_D^{25}$ = +53° (*c* 3.4, CH_3OH); ^1H NMR (500 MHz, CD_3OD) δ 7.55 (d, *J* = 7.5 Hz, 1H), 6.56 (dt, *J* = 3.5, 1.9 Hz, 1H), 6.07 (dt, *J* = 10.2, 2.1 Hz, 1H), 5.94 (d, *J* = 7.5 Hz, 1H), 5.84 – 5.79 (m, 1H), 4.74 (dq, *J* = 9.0, 2.7 Hz, 1H), 4.20 (d, *J* = 9.3, 1H), 3.84 (tt, *J* = 9.3, 4.7 Hz, 1H), 3.50 – 3.40 (m, 1H), 3.39 – 3.32 (m, 1H), 3.11 (t, *J* = 7.2, 2H), 3.03 (s, 3H), 2.46 (dd, *J* = 14.0, 5.2 Hz, 1H), 2.33 (dd, *J* = 14.0, 7.8 Hz, 1H), 1.98 - 1.87 (m, 1H), 1.76 (dtd, *J* = 14.2, 9.7, 4.9 Hz, 1H), 1.50 (sext, *J* = 7.2 Hz, 2H), 1.45 (s, 9H), 0.89 (t, *J* = 7.5 Hz, 3H); ^{13}C NMR (125 MHz, CD_3OD) δ 172.6, 170.2, 167.53, 167.46, 158.3, 157.8, 143.5, 135.1, 127.1, 97.1, 81.1, 80.4, 78.1, 48.9, 47.4, 46.6, 42.7, 42.1, 36.6, 32.2, 28.8, 23.5, 11.8; HRMS (ESI) calcd for $C_{25}H_{42}N_9O_6$ $[M+H]^+$ 564.3253, found 564.3249.

***tert*-Butyl ((*S*)-1-(((2*S*,3*S*,6*R*)-6-(4-amino-2-oxopyrimidin-1(2*H*)-yl)-2-(dimethylcarbamoyl)-3,6-dihydro-2*H*-pyran-3-yl)amino)-5-(1-methylguanidino)-1-oxopentan-3-yl)carbamate (10).** The mono-formic acid salt of **5** (78.0 mg, 0.134 mmol) was dissolved in 2 M dimethylamine in MeOH (1.5 mL). Activated 3 Å molecular sieves were added and the mixture was shaken in an incubator at 37 °C. After 2 d, the reaction was concentrated under a stream of nitrogen and then vacuum to yield a white solid, which was dissolved in a minimal amount of deionized water and purified using automated flash chromatography (C18, 30 g, 0-50% B) to yield the mono formic acid salt of **10** (53.1 mg, 67%) as an amorphous white solid: $[\alpha]_D^{24}$ = +50° (*c* 2.7, CH_3OH); ^1H NMR (500 MHz, CD_3OD) δ 7.43 (d, *J* = 7.5 Hz, 1H), 6.54 (q, *J* = 2.2 Hz, 1H), 6.33 – 6.15 (m, 1H), 5.96 – 5.80 (m, 2H), 4.90 – 4.95 (m, 1H), 4.68 (d, *J* = 6.9 Hz, 1H), 3.85 – 3.76 (m, 1H), 3.50 – 3.31 (m, 2H), 3.12 (s, 3H), 3.03 (s, 3H), 2.87 (s, 3H), 2.44 (dd, *J* = 14.1, 5.7 Hz, 1H), 2.34 (dd, *J* = 14.1, 7.6 Hz, 1H), 1.82 – 1.96 (m, 1H), 1.69 – 1.79 (m, 1H), 1.45

(s, 9H); ^{13}C NMR (125 MHz, CD_3OD) δ 172.7, 169.2, 168.8, 167.7, 158.3, 158.2, 157.8, 143.5, 134.4, 126.1, 96.5, 80.4, 75.0, 49.9, 47.4, 45.1, 42.5, 37.7, 36.6, 36.4, 32.4, 28.8; HRMS (ESI) calcd for $C_{24}H_{40}N_9O_6$ $[M+H]^+$ 550.3096, found 550.3112.

***tert*-Butyl ((*S*)-1-(((2*S*,3*S*,6*R*)-6-(4-amino-2-oxopyrimidin-1(2*H*)-yl)-2-(prop-2-yn-1-ylcarbamoyl)-3,6-dihydro-2*H*-pyran-3-yl)amino)-5-(1-methylguanidino)-1-oxopentan-3-yl)carbamate (11).** The mono-formic acid salt of **5** (150.7 mg, 0.259 mmol) was dissolved in a 1:4 (v/v) ratio of anhydrous MeOH to propargylamine solution (2.5 mL) and stirred in a sealed vial. After 2 d, the reaction was concentrated under a stream of nitrogen then vacuum to yield an orange/white solid, which was dissolved in a minimal amount of deionized water and purified using automated flash chromatography (C18, 30 g, 0-50% B) to yield the mono-formic acid salt of **11** (143.2 mg, 91%) as an amorphous orange/white solid: $[\alpha]_D^{22}$ = +29° (*c* 0.91, CH_3OH); ^1H NMR (400 MHz, CD_3OD) δ 7.56 (d, *J* = 7.5 Hz, 1H), 6.58 (dt, *J* = 3.4, 1.8 Hz, 1H), 6.08 (dt, *J* = 10.2, 2.1 Hz, 1H), 5.95 (d, *J* = 7.3 Hz, 1H), 5.83 (ddd, *J* = 10.3, 2.6, 1.5 Hz, 1H), 4.75 (dq, *J* = 9.3, 2.6 Hz, 1H), 4.26 (d, *J* = 9.3 Hz, 1H), 3.98 (dd, *J* = 17.4, 2.5 Hz, 1H), 3.89 (dd, *J* = 17.4, 2.6 Hz, 1H), 3.81 – 3.87 (m, 1H), 3.41 – 3.50 (m, 1H), 3.49 – 3.32 (m, 1H), 3.05 (s, 3H), 2.57 (t, *J* = 2.5 Hz, 1H), 2.48 (dd, *J* = 13.9, 5.2 Hz, 1H), 2.38 – 2.30 (m, 1H), 2.01 – 1.91 (m, 1H), 1.75 (dtd, *J* = 14.3, 9.8, 4.8 Hz, 1H), 1.45 (s, 9H); ^{13}C NMR (125 MHz, CD_3OD) δ 172.6, 170.2, 167.5, 158.3, 157.8, 143.5, 135.1, 127.1, 97.1, 81.1, 80.4, 78.1, 48.9, 47.4, 46.6, 42.7, 42.1, 36.6, 32.2, 28.8, 23.5, 11.8; HRMS (ESI) calcd for $C_{25}H_{38}N_9O_6$ $[M+H]^+$ 460.2940, found 460.2935.

***tert*-Butyl ((*S*)-1-(((2*S*,3*S*,6*R*)-6-(4-amino-2-oxopyrimidin-1(2*H*)-yl)-2-(but-3-yn-1-ylcarbamoyl)-3,6-dihydro-2*H*-pyran-3-yl)amino)-5-(1-methylguanidino)-1-oxopentan-3-yl)carbamate (12).** The mono-formic acid salt of **5** (158.3 mg, 0.272 mmol) was dissolved in a 1:4 (v/v) ratio of anhydrous MeOH to 3-butynylamine solution (2.5 mL) and stirred in a sealed vial. After 24 h, the reaction was concentrated under a stream of nitrogen then vacuum to yield a yellow/white solid, which was dissolved in a minimal amount of deionized water and purified

using automated flash chromatography (C18, 30 g, 0-30% B) to yield the mono-formic acid salt of **12** (126.5 mg, 75%) as an amorphous off-white solid: $[\alpha]_D^{23}$ = +46° (*c* 6.3, CH_3OH); ^1H NMR (400 MHz, CD_3OD) δ 7.56 (d, *J* = 7.5 Hz, 1H), 6.58 (dt, *J* = 3.5, 1.9 Hz, 1H), 6.07 (dt, *J* = 10.3, 2.1 Hz, 1H), 5.95 (d, *J* = 7.6 Hz, 1H), 5.83 (d, *J* = 10.3 Hz, 1H), 4.74 (dq, *J* = 9.2, 2.6 Hz, 1H), 4.21 (dd, *J* = 9.3 Hz, 1H), 3.91 – 3.80 (m, 1H), 3.54 – 3.34 (m, 2H), 3.31 (t, *J* = 7.2 Hz, 1H), 3.09 (t, *J* = 6.8 Hz, 1H), 3.04 (s, 3H), 2.59 (td, *J* = 6.8, 2.6 Hz, 1H), 2.48 (dd, *J* = 13.9, 5.1 Hz, 1H), 2.36 (tt, *J* = 7.4, 2.5 Hz, 2H), 2.28 (t, *J* = 2.7 Hz, 1H), 2.01 – 1.88 (m, 1H), 1.77 (dtd, *J* = 14.2, 9.7, 4.9 Hz, 1H), 1.46 (s, 9H); ^{13}C NMR (125 MHz, CD_3OD) δ 172.6, 170.4, 167.4, 165.5, 158.3, 157.9, 157.6, 143.6, 135.0, 127.1, 97.1, 82.1, 81.1, 80.5, 77.9, 71.0, 47.4, 46.8, 42.7, 39.3, 36.6, 32.2, 28.8, 19.7; HRMS (ESI) calcd for $C_{26}H_{40}N_9O_6$ $[M+H]^+$ 574.3096, found 574.3104.

***tert*-Butyl ((*S*)-1-(((2*S*,3*S*,6*R*)-6-(4-amino-2-oxopyrimidin-1(2*H*)-yl)-2-((2-hydroxyethyl)carbamoyl)-3,6-dihydro-2*H*-pyran-3-yl)amino)-5-(1-methylguanidino)-1-oxopentan-3-yl)carbamate (13).** The mono-formic acid salt of **5** (82.4 mg, 0.141 mmol) was dissolved in a 1:6 ratio (v/v) solution of anhydrous MeOH to ethanolamine (1.75 mL) and stirred in a sealed vial. After 18 h, the reaction was concentrated under a stream of nitrogen then vacuum to yield a white solid, which was dissolved in a minimal amount of deionized water and purified using automated flash chromatography (C18, 30 g, 0-50% B) to yield the mono-formic acid salt of **13** (71.8 mg, 77%) as an amorphous white solid: $[\alpha]_D^{25}$ = +47° (*c* 3.6, CH_3OH); ^1H NMR (500 MHz, CD_3OD) δ 7.57 (d, *J* = 7.5 Hz, 1H), 6.58 (dt, *J* = 3.5, 1.8 Hz, 1H), 6.07 (dt, *J* = 10.5, 2.4 Hz, 1H), 5.94 (d, *J* = 7.5 Hz, 1H), 5.81 – 5.86 (m, 1H), 4.76 (dq, *J* = 9.2, 2.6 Hz, 1H)), 4.21 (d, *J* = 9.3 Hz, 1H), 3.88 – 3.80 (m, 1H), 3.59 (t, *J* = 5.8 Hz, 2H), 3.50 – 3.41 (m, 1H), 3.38 – 3.32 (m, 1H), 3.29 (t, *J* = 5.8 Hz, 2H), 3.04 (s, 3H), 2.48 (dd, *J* = 14.0, 5.1 Hz, 1H), 2.34 (dd, *J* = 14.0, 7.9 Hz, 1H), 2.01 – 1.90 (m, 1H), 1.76 (dtd, *J* = 14.2, 9.7, 4.7 Hz, 1H), 1.46 (s, 9H); ^{13}C NMR (125 MHz, CD_3OD) δ 172.7, 170.6, 168.1, 167.6, 158.3, 157.9, 143.4, 135.0, 127.2, 97.2,

81.2, 80.5, 78.1, 61.4, 58.9, 48.9, 47.4, 46.8, 42.7, 36.6, 32.2, 28.8; HRMS (ESI) calcd for $C_{24}H_{40}N_9O_7$ [M+H]$^+$ 566.3045, found 566.3045.

***tert*-Butyl ((*S*)-1-(((2*S*,3*S*,6*R*)-6-(4-amino-2-oxopyrimidin-1(2*H*)-yl)-2-(phenethylcarbamoyl)-3,6-dihydro-2*H*-pyran-3-yl)amino)-5-(1-methylguanidino)-1-oxopentan-3-yl)carbamate (14).** The mono-formic acid salt of **5** (77.4 mg, 0.133 mmol) was dissolved in a 1:6 ratio (v/v) solution of anhydrous MeOH to phenethylamine (1.75 mL) and stirred for 18 h in a sealed vial. The reaction was concentrated under a stream of nitrogen and then vacuum to yield a white solid, which was dissolved in a minimal amount of deionized water and purified using automated flash chromatography (C18, 30 g, 0-50% B) to yield the mono-formic acid salt of **14** (72.1 mg, 81%) as an amorphous white solid: $[\alpha]_D^{25}$ = +45° (*c* 3.6, CH_3OH); ^{1}H NMR (500 MHz, CD_3OD) δ 7.52 (d, *J* = 7.4 Hz, 1H), 7.31 – 7.22 (m, 2H), 7.22 – 7.11 (m, 3H), 6.56 (dt, *J* = 3.4, 1.8 Hz, 1H), 6.07 (dt, *J* = 10.2, 2.1 Hz, 1H), 5.94 (d, *J* = 7.2 Hz, 1H), 5.82 (ddd, *J* = 10.2, 2.7, 1.4 Hz, 1H), 4.74 (dq, *J* = 9.4, 2.6 Hz, 1H), 4.19 (d, *J* = 9.3 Hz, 1H), 3.85 (tt, *J* = 9.2, 4.6 Hz, 1H), 3.51 – 3.33 (m, 4H), 3.03 (s, 3H), 2.78 (t, *J* = 7.4 Hz, 2H), 2.47 (dd, *J* = 13.9, 5.1 Hz, 1H), 2.34 (dd, *J* = 14.0, 8.0 Hz, 1H), 1.90 – 2.00 (m, 1H), 1.77 (m, 1H), 1.44 (s, 9H); ^{13}C NMR (125 MHz, CD_3OD) δ 172.6, 170.2, 168.4, 167.6, 158.3, 158.0, 157.8, 143.3, 140.3, 135.0, 129.9, 129.5, 127.4, 127.1, 97.2, 81.1, 80.5, 77.9, 47.4, 46.7, 42.7, 41.8, 36.5, 36.3, 32.2, 28.8; HRMS (ESI) calcd for $C_{30}H_{44}N_9O_6$ [M+H]$^+$ 626.3409, found 626.3420.

General Method for Deprotection of Amides 6–14

BOC-protected amide (**6–14)** mono-formic acid salt was placed in an ice bath (0 °C) and dissolved in a 1:1 mixture of CH_2Cl_2 to trifluoroacetic acid (TFA). The solution was warmed to rt over 2-3 h, after which it was concentrated and suspended in PhH for azeotropic removal of excess TFA under vacuum (x3). The residue was dried extensively under vacuum before being

dissolved in a minimal amount of water and purified using automated flash chromatography (C18, 30 g). Compounds were isolated as their tri-TFA salts or as the trihydrochloride salts.

(2*S*,3*S*,6*R*)-6-(4-Amino-2-oxopyrimidin-1(2*H*)-yl)-3-((*S*)-3-amino-5-(1-methylguanidino)pentanamido)-3,6-dihydro-2*H*-pyran-2-carboxamide (P10, 2). Amide **6** mono-formic acid salt (64.9 mg, 0.122 mmol) was treated with CH_2Cl_2:TFA (1.5 mL) according to the general procedure and purified (0-20% B) to yield the mono-formic acid salt of **2**, which was dissolved in 0.3% (v/v) conc. $HCl_{(aq.)}$ in MeOH (5 mL) and concentrated, this dissolution/concentration being repeated a total of three times. Extensive drying under vacuum yielded **2** as the trihydrochloride salt (19.8 mg, 31%). Spectral data were in accord with those previously reported.[1]

(2*S*,3*S*,6*R*)-6-(4-Amino-2-oxopyrimidin-1(2*H*)-yl)-3-((*S*)-3-amino-5-(1-methylguanidino)pentanamido)-*N*-methyl-3,6-dihydro-2*H*-pyran-2-carboxamide (15). Amide (**7**) mono-formic acid salt (44.2 mg, 0.076 mmol) was deprotected in CH_2Cl_2:TFA (2.0 mL) according to the general procedure and purified (0-30% B) to yield the mono-formic acid salt of **15**, which was converted to the trihydrochloride by dissolution in 0.3% (v/v) conc. $HCl_{(aq.)}$ in MeOH (5 mL) and concentration, this dissolution/concentration being repeated a total of three times. Extensive drying under vacuum yielded **15** as the hydrochloride salt (38.0 mg, 92%): $[\alpha]_D^{24}$ = +48° (*c* 0.90, CH_3OH); ^1H NMR (500 MHz, CD_3OD) δ 7.57 (d, *J* = 7.5 Hz, 1H), 6.60 (dt, *J* = 3.3, 1.8 Hz, 1H), 6.07 (dt, *J* = 10.2, 2.0 Hz, 1H), 5.97 (d, *J* = 7.5 Hz, 1H), 5.86 (ddd, *J* = 10.2, 2.6, 1.4 Hz, 1H), 4.82 (dq, *J* = 9.3, 2.6 Hz, 1H), 4.22 (d, *J* = 9.3 Hz, 1H), 3.69 – 3.45 (m, 3H), 3.09 (s, 3H), 2.73 (s, 3H), 2.79 – 2.64 (m, 1H), 2.60 (dd, *J* = 15.6, 7.7 Hz, 1H), 2.16 – 2.00 (m, 2H); ^{13}C NMR (125 MHz, CD_3OD) δ 171.4, 171.1, 167.8, 158.4, 158.1, 143.3, 134.6, 127.7, 97.3, 81.3, 77.5, 48.4, 48.1, 46.9, 38.5, 36.7, 30.8, 26.1; HRMS (ESI) calcd for $C_{18}H_{30}N_9O_4$ $[M+H]^+$ 436.2415, found 436.2420.

(2*S*,3*S*,6*R*)-6-(4-Amino-2-oxopyrimidin-1(2*H*)-yl)-3-((*S*)-3-amino-5-(1-methylguanidino)pentanamido)-*N*-ethyl-3,6-dihydro-2*H*-pyran-2-carboxamide (16). Amide (**8**) mono-formic acid salt (72.4 mg, 0.122 mmol) was deprotected in CH_2Cl_2:TFA (4.0 mL) according to the general procedure and purified (0-30% B) to yield the tri-TFA salt of **16** (64.0 mg, 67%): $[\alpha]_D^{24}$ = +29° (*c* 0.85, CH_3OH); ^{1}H NMR (600 MHz, CD_3OD) δ 7.81 (d, *J* = 7.8 Hz, 1H), 6.60 (dt, *J* = 3.4, 1.8 Hz, 1H), 6.15 – 6.10 (m, 2H), 5.88 (ddd, *J* = 10.3, 2.7, 1.5 Hz, 1H), 4.85 (dtd, *J* = 9.4, 2.9, 2.0 Hz, 1H), 4.21 (d, *J* = 9.4 Hz, 1H), 3.65 – 3.59 (m, 1H), 3.58 – 3.44 (m, 2H), 3.21 (q, *J* = 7.2 Hz, 2H), 3.08 (s, 3H), 2.70 (dd, *J* = 15.7, 4.5 Hz, 1H), 2.58 (dd, *J* = 15.7, 7.7 Hz, 1H), 2.10 – 1.99 (m, 2H), 1.10 (t, *J* = 7.3 Hz, 3H); ^{13}C NMR (125 MHz, CD_3OD) δ 171.4, 169.9, 161.8, 158.3, 149.0, 146.8, 135.9, 126.1, 96.0, 80.9, 77.6, 48.4, 48.0, 46.6, 38.2, 36.6, 35.1, 30.7, 14.7; HRMS (ESI) calcd for $C_{19}H_{32}N_9O_4$ $[M+H]^+$ 450.2572, found 450.2576.

(2*S*,3*S*,6*R*)-6-(4-Amino-2-oxopyrimidin-1(2*H*)-yl)-3-((*S*)-3-amino-5-(1-methylguanidino)pentanamido)-*N*-propyl-3,6-dihydro-2*H*-pyran-2-carboxamide (17). Amide (**9**) mono-formic acid salt (68.3 mg, 0.112 mmol) was deprotected in CH_2Cl_2:TFA (4.0 mL) according to the general procedure and purified (0-50% B) to yield the tri-TFA salt of **17** (66.3 mg, 74%): $[\alpha]_D^{26}$ = +33° (*c* 3.3, CH_3OH); ^{1}H NMR (500 MHz, CD_3OD) δ 7.85 (d, *J* = 7.8 Hz, 1H), 6.60 (dt, *J* = 3.4, 1.8 Hz, 1H), 6.17 – 6.11 (m, 2H), 5.89 (ddd, *J* = 10.3, 2.7, 1.5 Hz, 1H), 4.88 – 4.84 (m, 1H), 4.24 (d, *J* = 9.4 Hz, 1H), 3.62 (qd, *J* = 7.1, 4.4 Hz, 1H), 3.58 – 3.44 (m, 2H), 3.13 (td, *J* = 7.1, 2.3 Hz, 2H), 3.08 (s, 3H), 2.71 (dd, *J* = 15.7, 4.5 Hz, 1H), 2.59 (dd, *J* = 15.5, 7.4 Hz, 1H), 2.10 – 1.99 (m, 2H), 1.52 (sext, *J* = 7.4 Hz, 2H), 0.90 (t, *J* = 7.4 Hz, 3H); ^{13}C NMR (125 MHz, CD_3OD) δ 171.4, 170.1, 162.2, 158.3, 149.7, 146.6, 135.8, 126.2, 96.1, 80.9, 77.6, 48.4, 48.0, 46.6, 42.0, 38.2, 36.6, 30.7, 23.5, 11.7; HRMS (ESI) calcd for $C_{20}H_{34}N_9O_4$ $[M+H]^+$ 464.2728, found 464.2734.

(2*S*,3*S*,6*R*)-6-(4-Amino-2-oxopyrimidin-1(2*H*)-yl)-3-((*S*)-3-amino-5-(1-methylguanidino)pentanamido)-*N*,*N*-dimethyl-3,6-dihydro-2*H*-pyran-2-carboxamide (18).

Amide (**10**) mono-formic acid salt (53.1 mg, 0.0891 mmol) was deprotected in CH_2Cl_2:TFA (4.0 mL) according to the general procedure and purified (0-50% B) to yield the mono-formic acid salt of **18**, which was converted to the trihydrochloride by dissolution in 0.3% (v/v) conc. $HCl_{(aq.)}$ in MeOH (5 mL) and concentration, this dissolution/concentration being repeated a total of three times. Extensive drying under vacuum yielded **18** as the trihydrochloride salt (8.7 mg, 18%): $[\alpha]_D^{21}$ = +49° (*c* 0.44, CH_3OH); ^1H NMR (500 MHz, CD_3OD) δ 7.83 (d, *J* = 7.8 Hz, 1H), 6.61 (q, *J* = 2.1 Hz, 1H), 6.27 (dt, *J* = 10.3, 2.3 Hz, 1H), 6.13 (d, *J* = 7.8 Hz, 1H), 5.90 (dt, *J* = 10.3, 2.1 Hz, 1H), 5.05 (dq, *J* = 7.8, 2.5 Hz, 1H), 4.83 (d, *J* = 8.1 Hz, 1H), 3.67 – 3.59 (m, 1H), 3.58 – 3.47 (m, 2H), 3.17 (s, 3H), 3.08 (s, 3H), 2.92 (s, 3H), 2.74 (dd, *J* = 16.3, 4.7 Hz, 1H), 2.63 (dd, *J* = 16.3, 7.4 Hz, 1H), 2.11 – 1.92 (m, 2H); ^{13}C NMR (125 MHz, CD_3OD) δ 171.3, 168.6, 161.4, 158.3, 148.6, 147.0, 135.8, 125.0, 95.5, 80.8, 75.1, 48.1, 47.9, 45.1, 37.7, 37.6, 36.8, 36.4, 30.7; HRMS (ESI) calcd for $C_{19}H_{32}N_9O_4$ $[M+H]^+$ 450.2572, found 450.2568.

(2*S*,3*S*,6*R*)-6-(4-Amino-2-oxopyrimidin-1(2*H*)-yl)-3-((*S*)-3-amino-5-(1-methylguanidino)pentanamido)-*N*-(prop-2-yn-1-yl)-3,6-dihydro-2*H*-pyran-2-carboxamide (19). Amide (**11**) mono-formic acid salt (143.2 mg, 0.236 mmol) was deprotected in CH_2Cl_2:TFA (5.0 mL) according to the general procedure and purified (0-20% B) to yield the tri-TFA salt of **19** (145.5 mg, 77%): $[\alpha]_D^{23}$ = +34° (*c* 1.8, CH_3OH); ^1H NMR (500 MHz, CD_3OD) δ 7.89 (d, *J* = 7.9 Hz, 1H), 6.62 (dt, *J* = 3.4, 1.8 Hz, 1H), 6.19 – 6.14 (m, 2H), 5.90 (ddd, *J* = 10.3, 2.7, 1.6 Hz, 1H), 4.87 (dtd, *J* = 9.3, 2.8, 2.0 Hz, 1H), 4.31 (d, *J* = 9.3 Hz, 1H), 3.99 (dd, *J* = 17.4, 2.5 Hz, 1H), 3.92 (dd, *J* = 17.4, 2.6 Hz, 1H), 3.66 – 3.60 (m, 1H), 3.59 – 3.46 (m, 2H), 3.09 (s, 3H), 2.73 (dd, *J* = 15.8, 4.6 Hz, 1H), 2.64 – 2.58 (m, 2H), 2.13 – 2.00 (m, 2H); ^{13}C NMR (125 MHz, CD_3OD) δ 171.4, 170.0, 168.7, 156.9, 152.0, 143.8, 133.9, 125.3, 95.3, 79.6, 78.9, 76.0, 70.9, 46.9, 46.6, 45.1, 36.6, 35.2, 29.2, 27.9; HRMS (ESI) calcd for $C_{20}H_{30}N_9O_4$ $[M+H]^+$ 460.2415, found 460.2409.

(2*S*,3*S*,6*R*)-6-(4-Amino-2-oxopyrimidin-1(2*H*)-yl)-3-((*S*)-3-amino-5-(1-methylguanidino)pentanamido)-*N*-(but-3-yn-1-yl)-3,6-dihydro-2*H*-pyran-2-carboxamide (20). Amide (**12**) mono-formic acid salt (126.5 mg, 0.204 mmol) was deprotected in CH_2Cl_2:TFA (5.0 mL) according to the general procedure and purified (0-100% B) to yield the tri-TFA salt of **20** (112.5 mg, 68%): $[\alpha]_D^{22}$ = +36° (*c* 5.9, CH_3OH); ^{1}H NMR (500 MHz, CD_3OD) δ 7.91 (d, *J* = 7.9 Hz, 1H), 6.61 (dt, *J* = 3.4, 1.8 Hz, 1H), 6.21 – 6.12 (m, 2H), 5.90 (ddd, *J* = 10.2, 2.7, 1.5 Hz, 1H), 4.89 – 4.84 (m, 1H), 4.27 (d, *J* = 9.3 Hz, 1H), 3.67 – 3.61 (m, 1H), 3.60 – 3.47 (m, 2H), 3.38 – 3.28 (m, 2H), 3.10 (s, 3H), 2.73 (dd, *J* = 15.8, 4.5 Hz, 1H), 2.62 (dd, *J* = 15.8, 7.6 Hz, 1H), 2.41 – 2.36 (m, 2H), 2.32 – 2.30 (m, 1H), 2.13 – 2.01 (m, 2H); ^{13}C NMR (125 MHz, CD_3OD) δ 171.4, 170.5, 165.2, 158.3, 154.6, 144.8, 135.2, 126.9, 96.9, 82.0, 81.0, 77.6, 71.0, 48.4, 48.0, 46.7, 39.2, 38.3, 36.6, 30.7, 19.6; HRMS (ESI) calcd for $C_{21}H_{32}N_9O_4$ $[M+H]^+$ 474.2572, found 474.2577.

(2*S*,3*S*,6*R*)-6-(4-Amino-2-oxopyrimidin-1(2*H*)-yl)-3-((*S*)-3-amino-5-(1-methylguanidino)pentanamido)-*N*-(2-hydroxyethyl)-3,6-dihydro-2*H*-pyran-2-carboxamide (21). Amide (**13**) mono-formic acid salt (71.8 mg, 0.109 mmol) was deprotected in CH_2Cl_2:TFA (5.0 mL) according to the general procedure and purified (0-30% B) to yield the tri-TFA salt of **21** (30.6 mg, 35%): $[\alpha]_D^{24}$ = +33° (*c* 1.5, CH_3OH); ^{1}H NMR (500 MHz, CD_3OD) δ 7.92 (d, *J* = 7.9 Hz, 1H), 6.61 (dt, *J* = 3.4, 1.8 Hz, 1H), 6.21 – 6.14 (m, 2H), 5.90 (ddd, *J* = 10.3, 2.7, 1.6 Hz, 1H), 4.92 – 4.87 (m, 1H), 4.27 (d, *J* = 9.4 Hz, 1H), 3.68 – 3.58 (m, 3H), 3.58 – 3.44 (m, 2H), 3.34 – 3.30 (m, 3H), 3.09 (s, 3H), 2.74 (dd, *J* = 15.9, 4.5 Hz, 1H), 2.62 (dd, *J* = 15.9, 7.7 Hz, 1H), 2.13 – 1.99 (m, 2H); ^{13}C NMR (125 MHz, CD_3OD) δ 171.5, 170.5, 161.9, 158.3, 149.2, 146.7, 135.8, 126.1, 96.1, 80.9, 77.7, 61.3, 48.3, 48.0, 46.7, 42.7, 38.1, 36.6, 30.7; HRMS (ESI) calcd for $C_{19}H_{32}N_9O_5$ $[M+H]^+$ 466.2521, found 466.2518.

(2*S*,3*S*,6*R*)-6-(4-Amino-2-oxopyrimidin-1(2*H*)-yl)-3-((*S*)-3-amino-5-(1-methylguanidino)pentanamido)-*N*-phenethyl-3,6-dihydro-2*H*-pyran-2-carboxamide (22).

Amide (**14**) mono-formic acid salt (72.1 mg, 0.107 mmol) was deprotected in CH_2Cl_2:TFA (4.0 mL) according to the general procedure and purified (0-50% B) to yield the mono-formic acid salt of **22**, which was converted to the trihydrochloride by dissolution in 0.3% (v/v) conc. $HCl_{(aq.)}$ in MeOH (5 mL) and concentration, this dissolution/concentration being repeated a total of three times. Extensive drying under vacuum yielded **22** as the trihydrochloride salt (50.8. mg, 75%): $[\alpha]_D^{24}$ = +36° (*c* 2.5, CH_3OH); ^1H NMR (400 MHz, CD_3OD) δ 7.88 (d, *J* = 7.9 Hz, 1H), 7.32 – 7.13 (m, 5H), 6.60 (dt, *J* = 3.3, 1.8 Hz, 1H), 6.20 – 6.12 (m, 2H), 5.90 (ddd, *J* = 10.3, 2.7, 1.6 Hz, 1H), 4.88 – 4.84 (m, 1H), 4.26 (d, *J* = 9.4 Hz, 1H), 3.69 – 3.60 (m, 1H), 3.59 – 3.47 (m, 2H), 3.46 – 3.33 (m, 2H), 3.08 (s, 3H), 2.80 (t, *J* = 7.5 Hz, 2H), 2.74 (dd, *J* = 15.8, 4.6 Hz, 1H), 2.63 (dd, *J* = 15.8, 7.7 Hz, 1H), 2.13 – 2.02 (m, 2H); ^{13}C NMR (125 MHz, CD_3OD) δ 171.4, 170.1, 161.6, 158.2, 148.7, 147.0, 140.3, 135.9, 129.9, 129.5, 127.4, 126.0, 95.9, 80.9, 77.5, 48.4, 48.1, 46.6, 41.8, 38.1, 36.8, 36.3, 30.7; HRMS (ESI) calcd for $C_{25}H_{36}N_9O_4$ $[M+H]^+$ 526.2885, found 526.2883.

^{1}H and ^{13}C NMR Spectra for Compounds 5-22

Figure S1. ^{1}H NMR of **5** (500 MHz, CD_3OD)

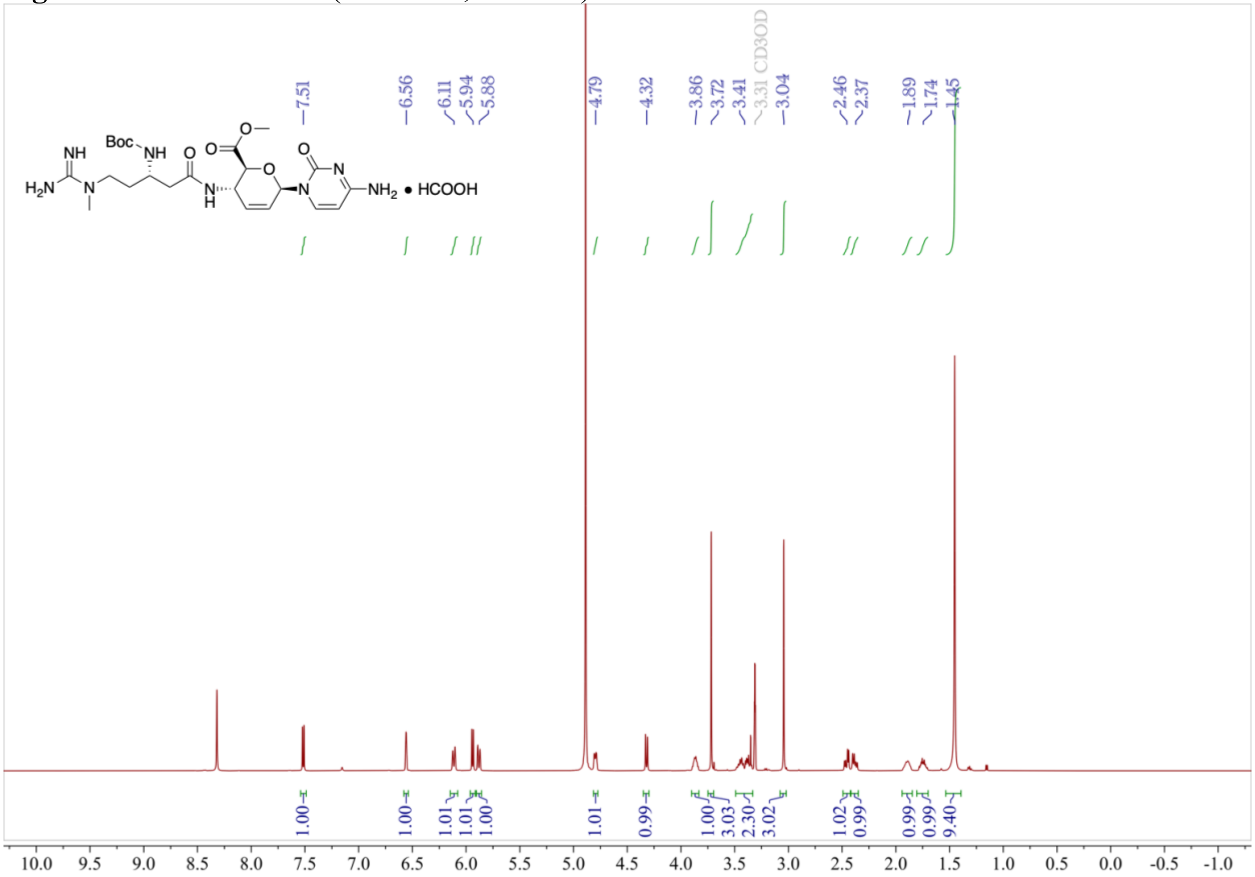

Figure S2. ^{13}C NMR of **5** (125 MHz, CD_3OD)

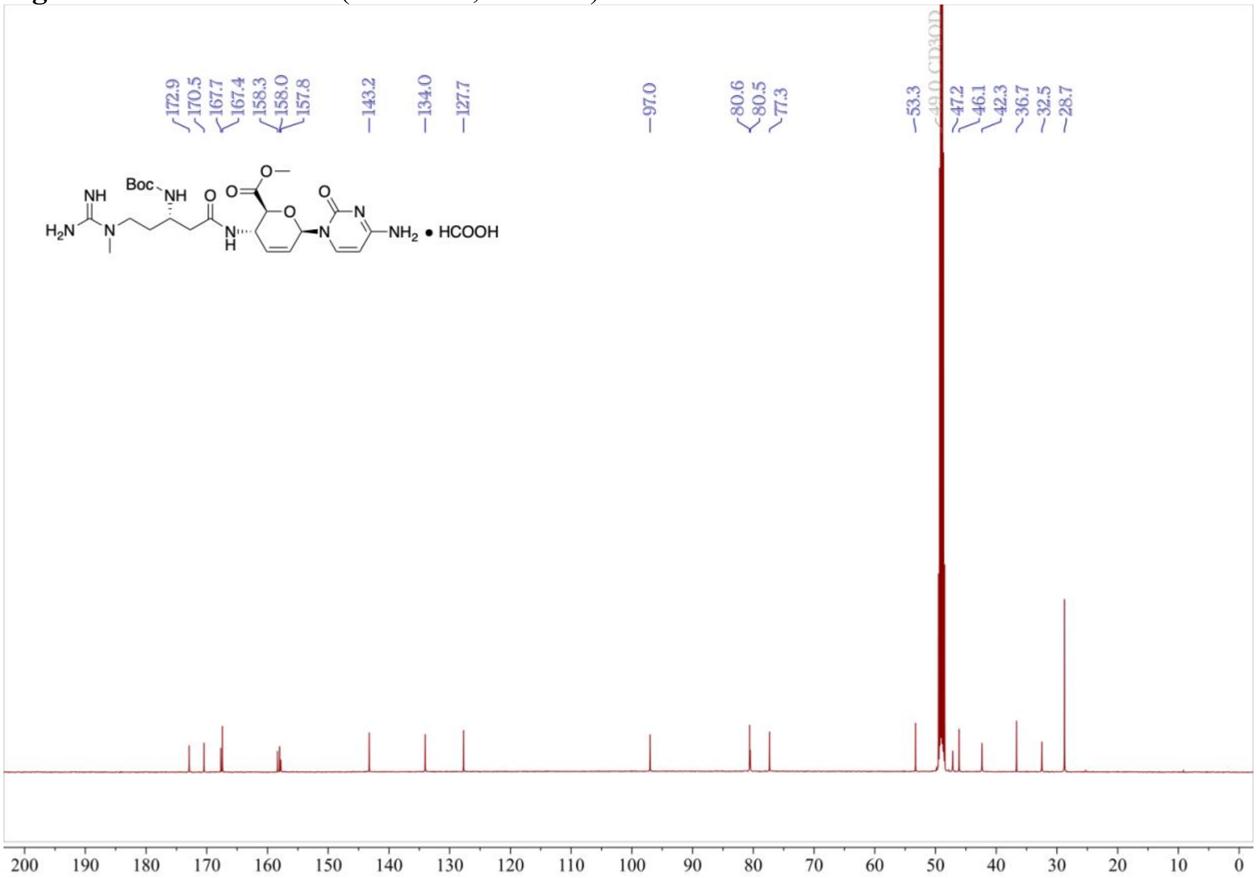

Figure S3. ^{1}H NMR of **6** (500 MHz, CD_3OD)

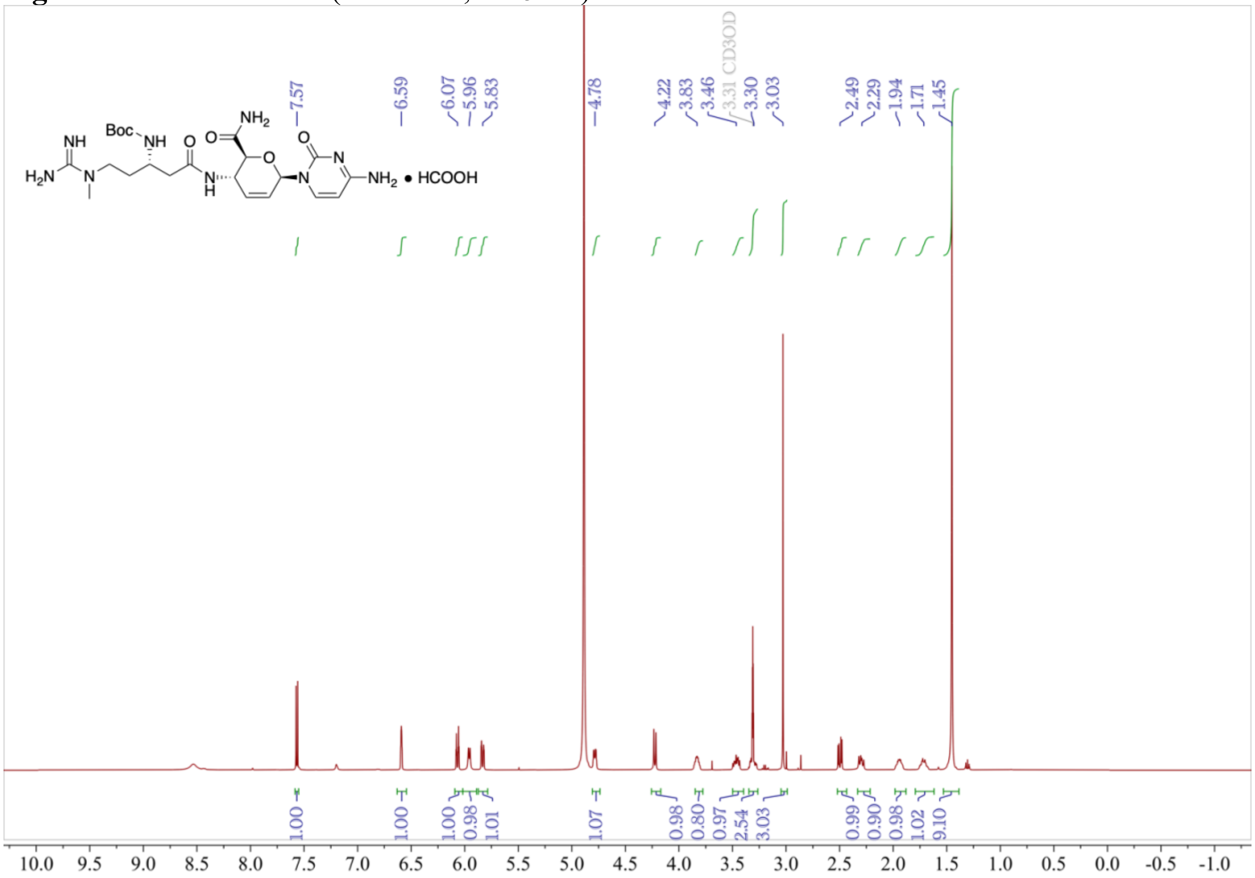

Figure S4. ^{13}C NMR of **6** (125 MHz, CD_3OD)

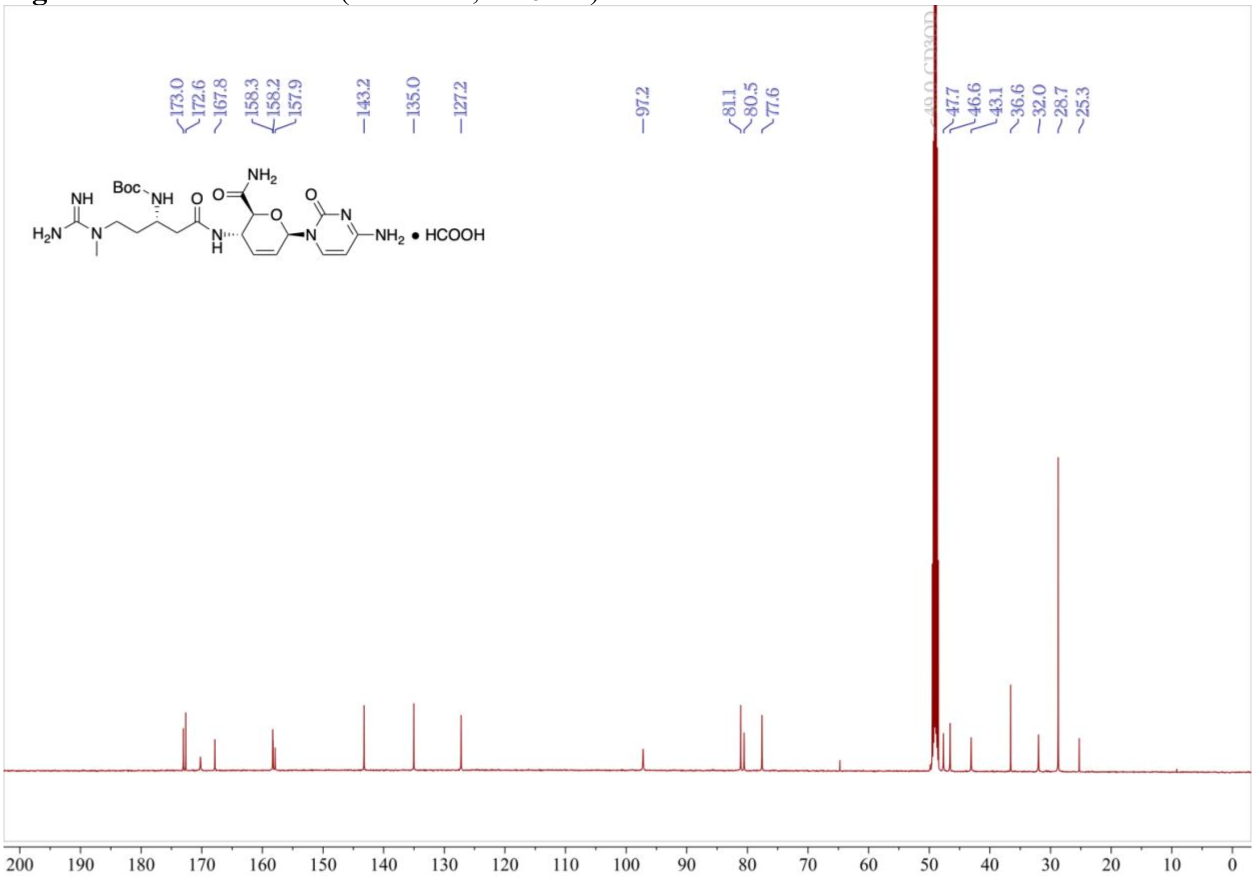

Figure S5. ^{1}H NMR of **7** (500 MHz, CD_3OD)

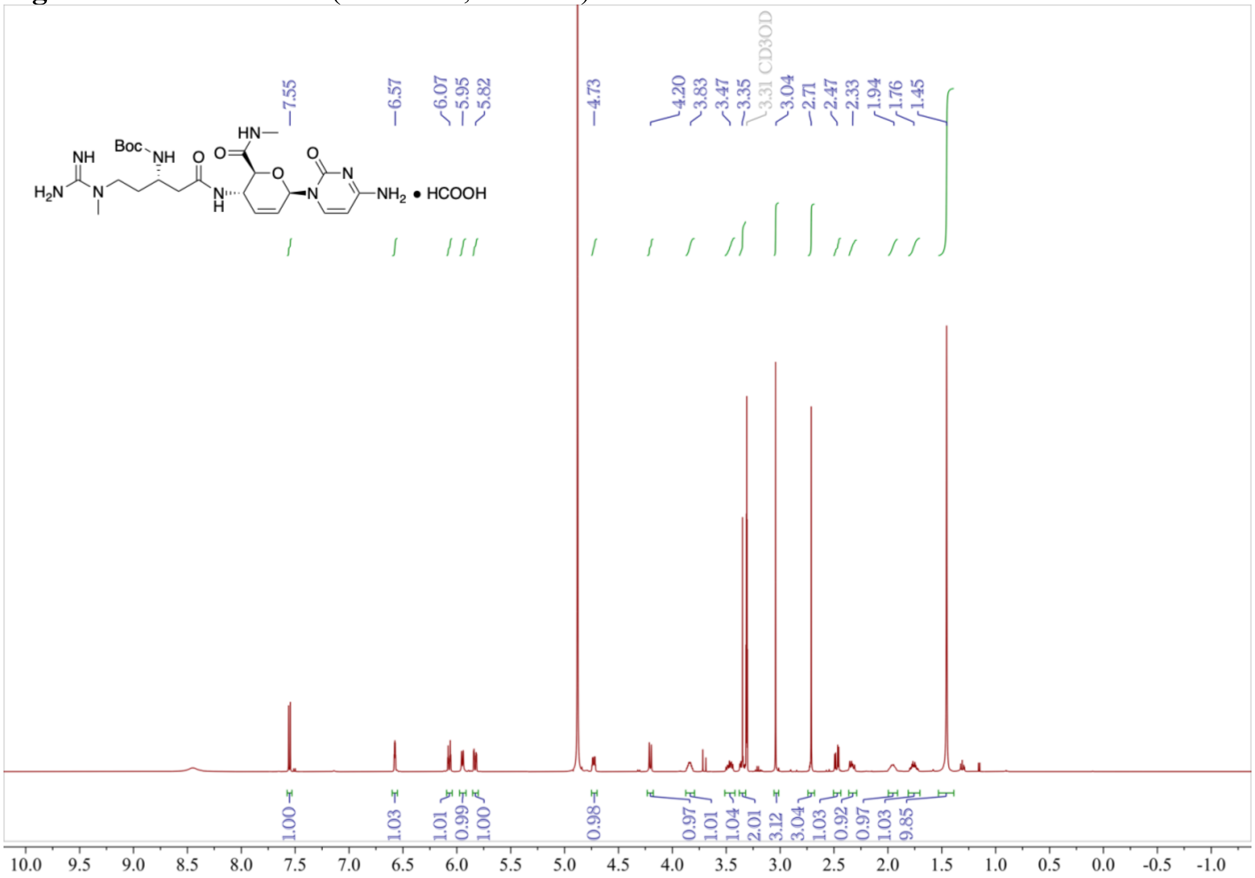

Figure S6. ^{13}C NMR of **7** (125 MHz, CD_3OD)

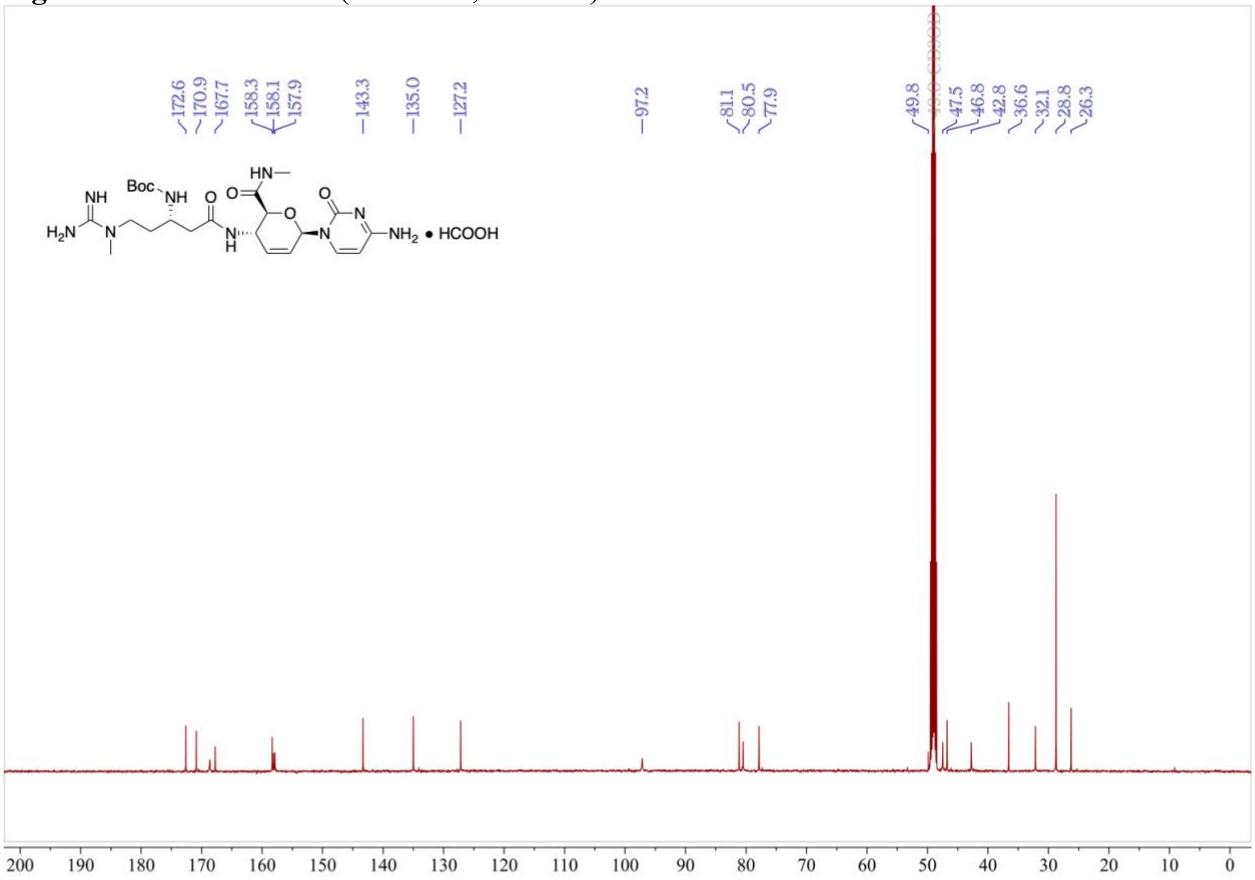

Figure S7. ^{1}H NMR of **8** (400 MHz, CD_3OD)

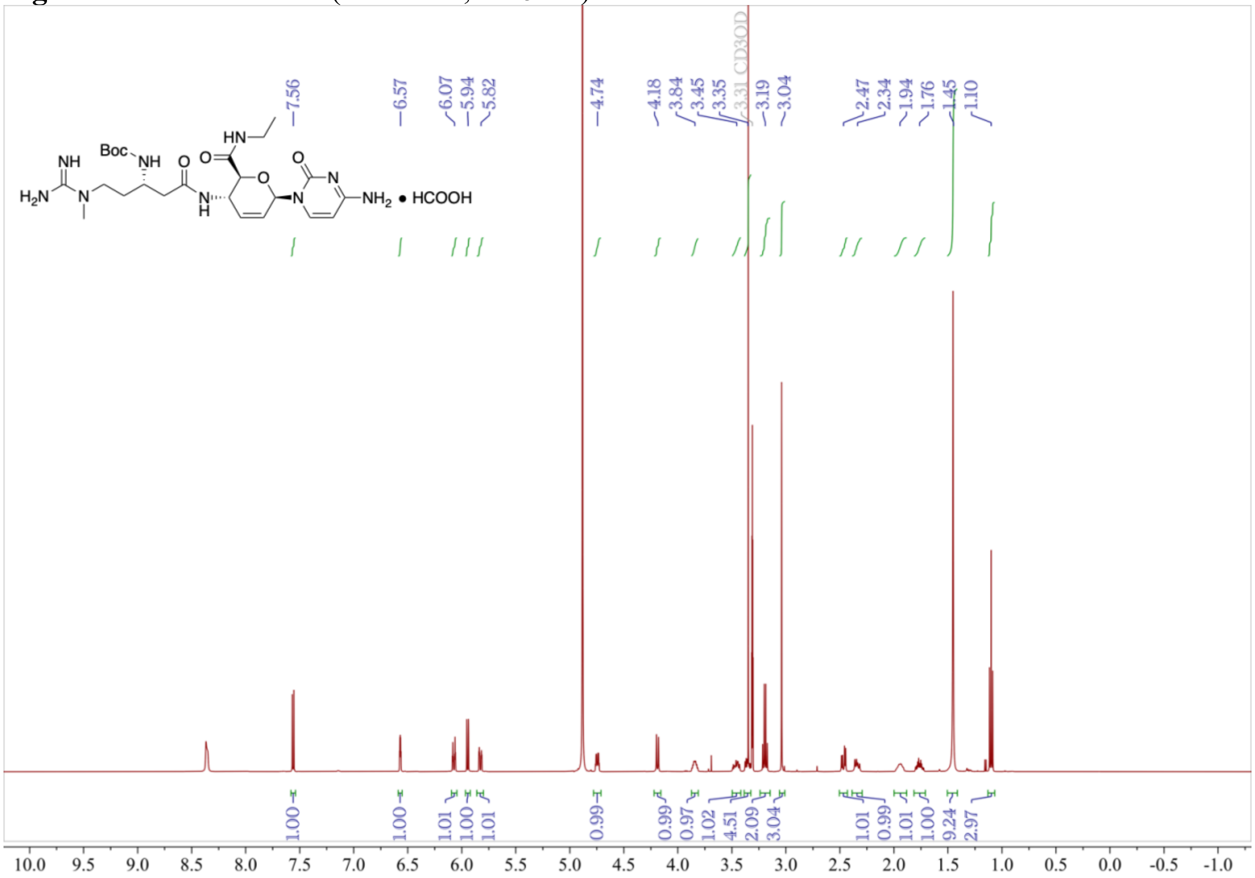

Figure S8. ^{13}C NMR of **8** (125 MHz, CD_3OD)

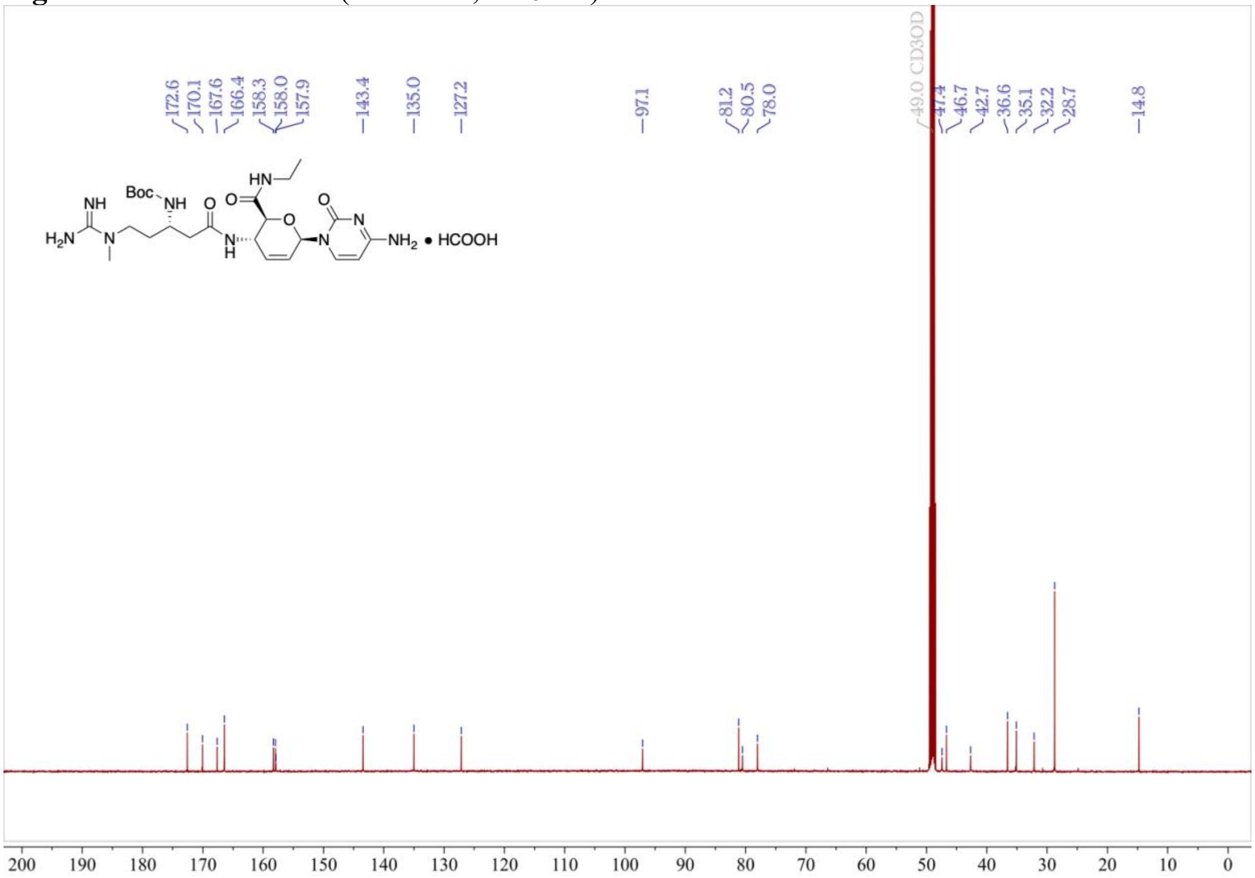

Figure S9. ^{1}H NMR of **9** (500 MHz, CD_3OD)

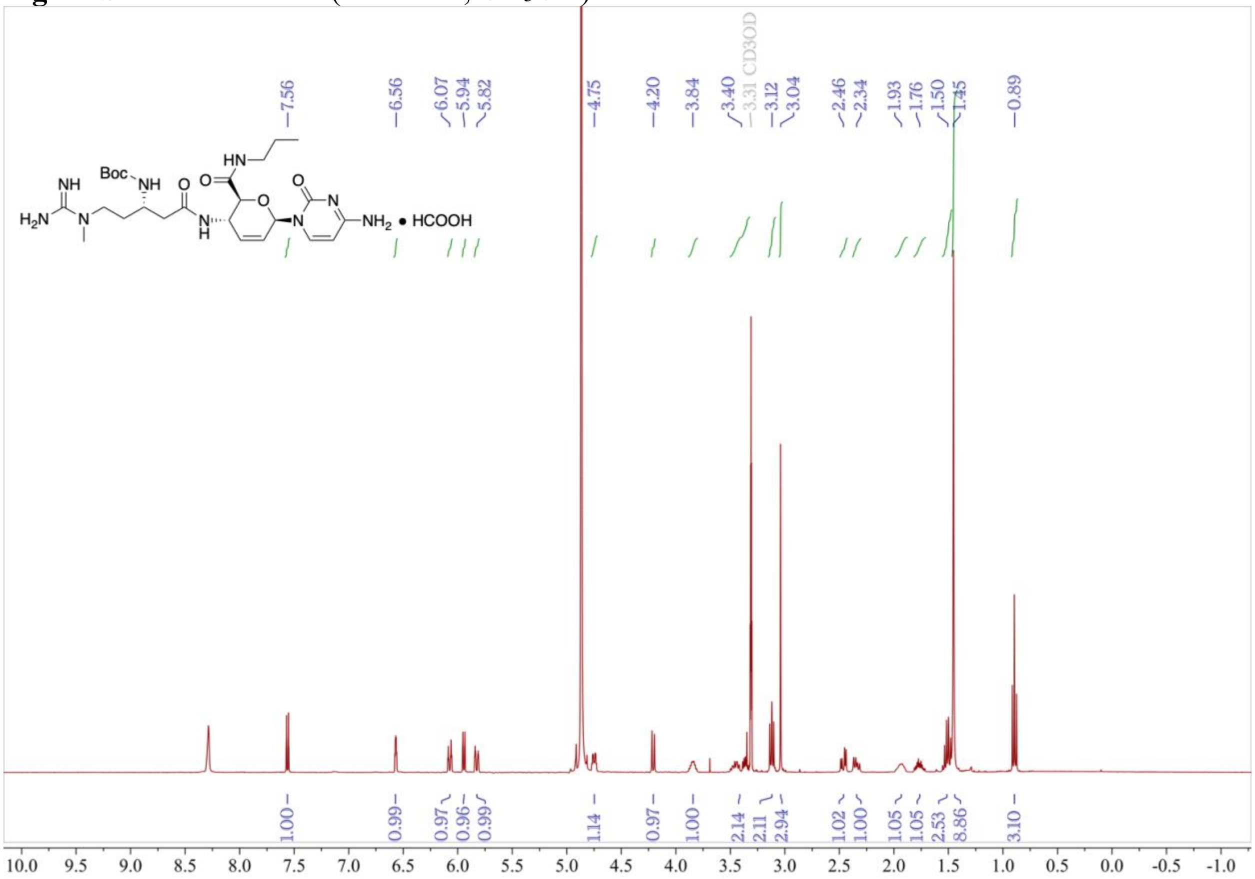

Figure S10. ^{13}C NMR of **9** (500 MHz, CD_3OD)

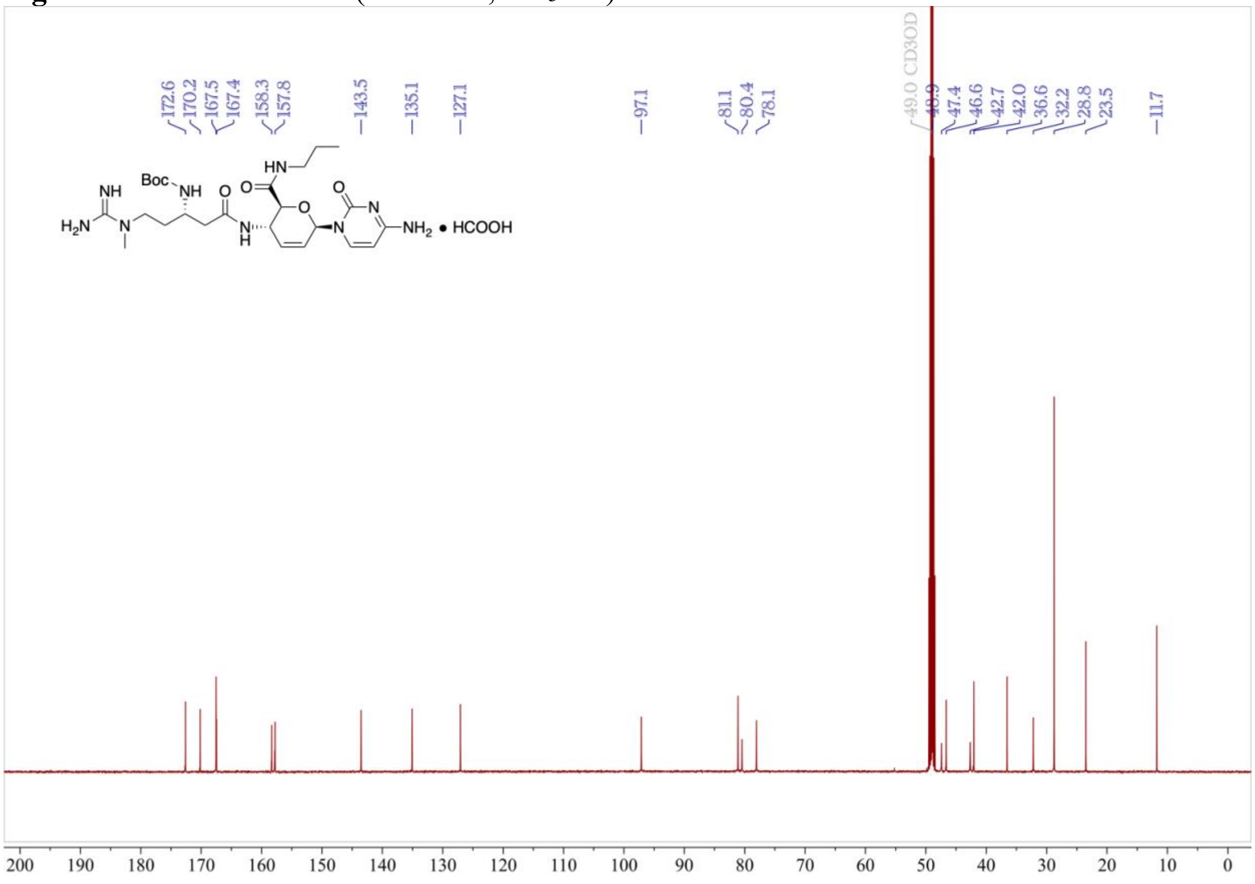

Figure S11. ^{1}H NMR of **10** (500 MHz, CD_3OD)

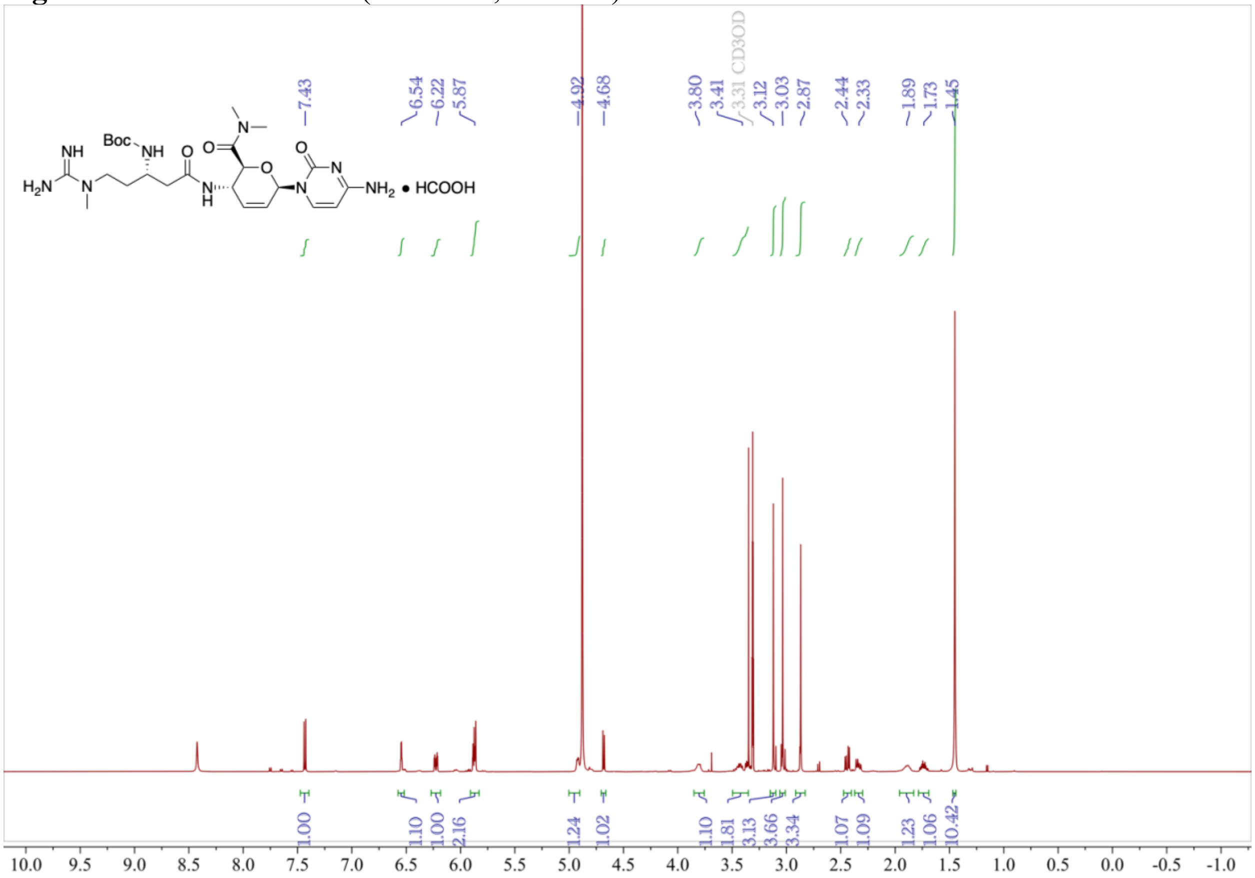

Figure S12. ^{13}C NMR of **10** (125 MHz, CD_3OD)

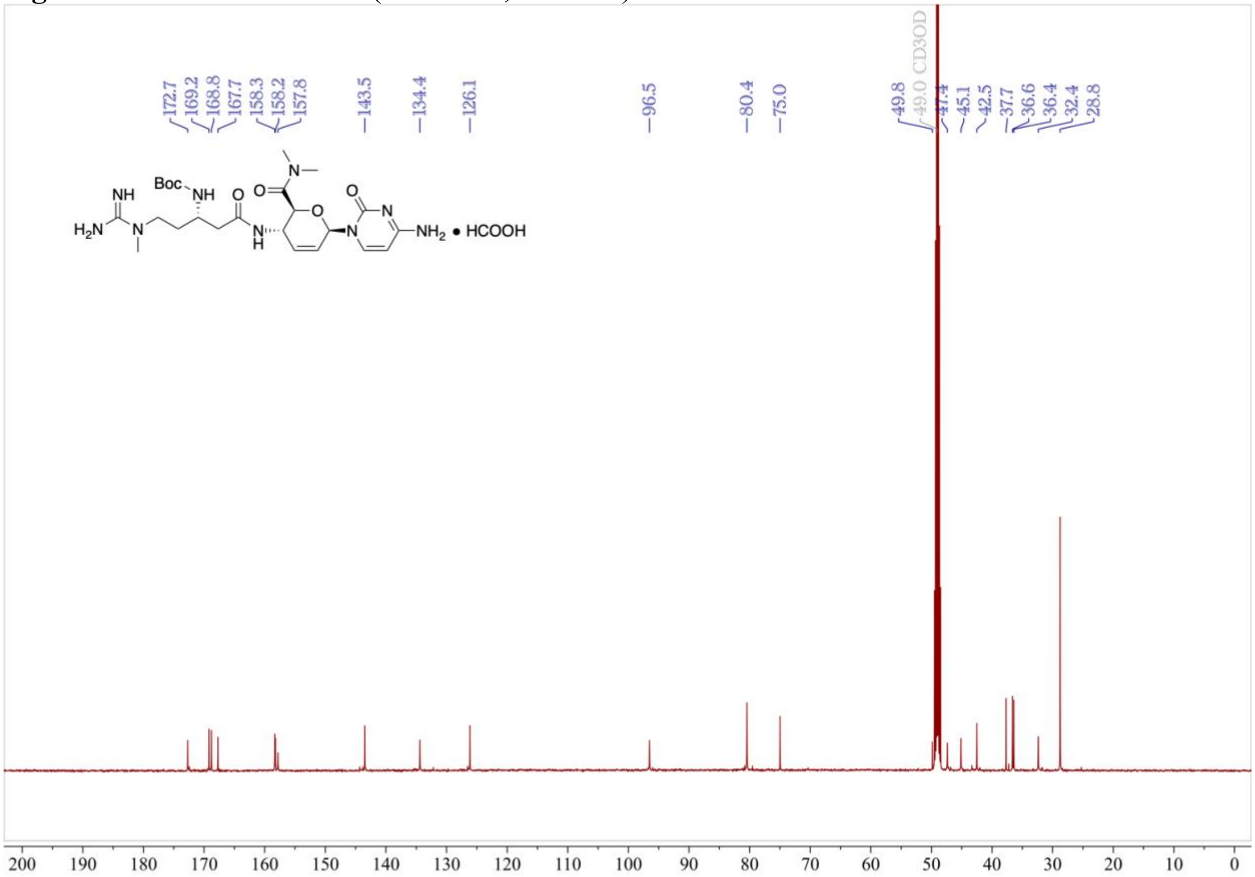

Figure S13. ^{1}H NMR of **11** (400 MHz, CD_3OD)

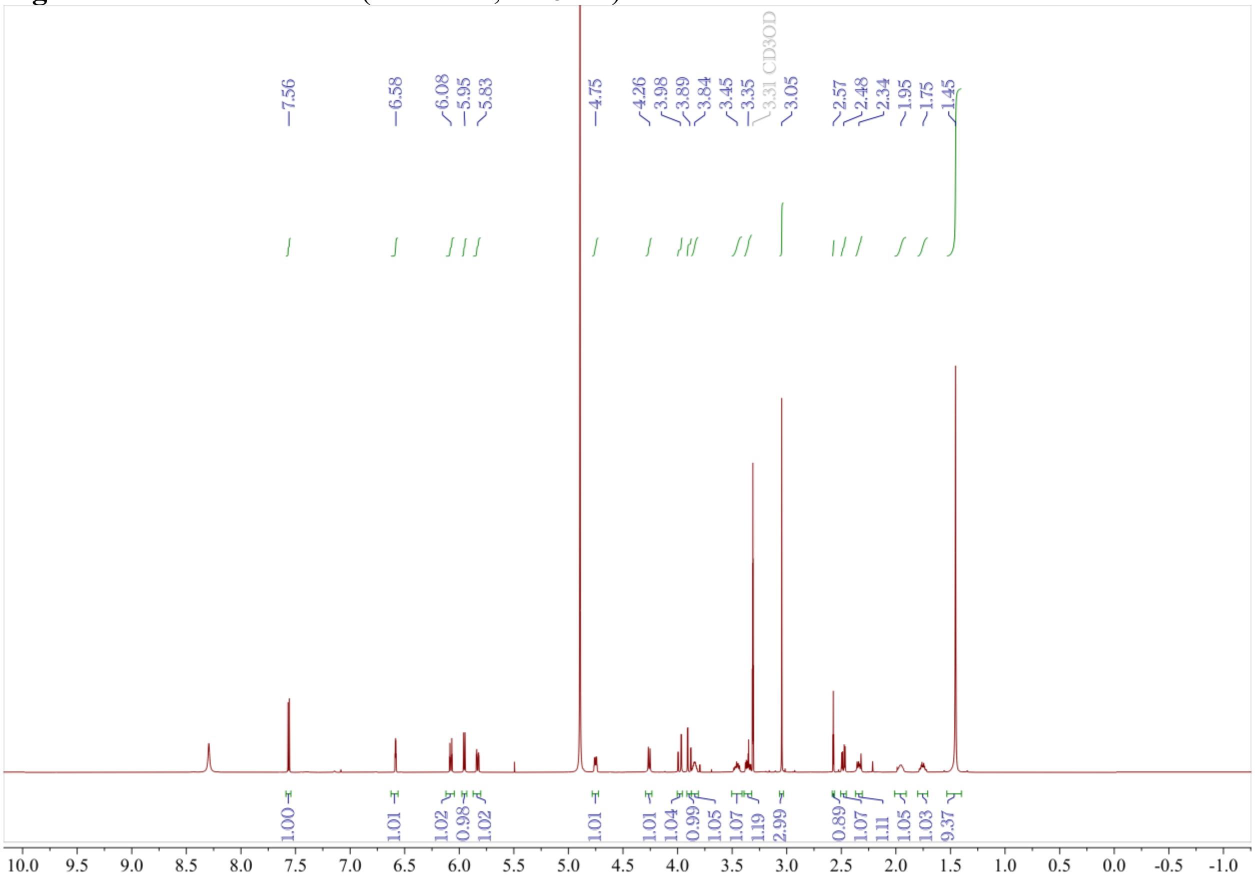

Figure S14. ^{13}C NMR of **11** (125 MHz, CD_3OD)

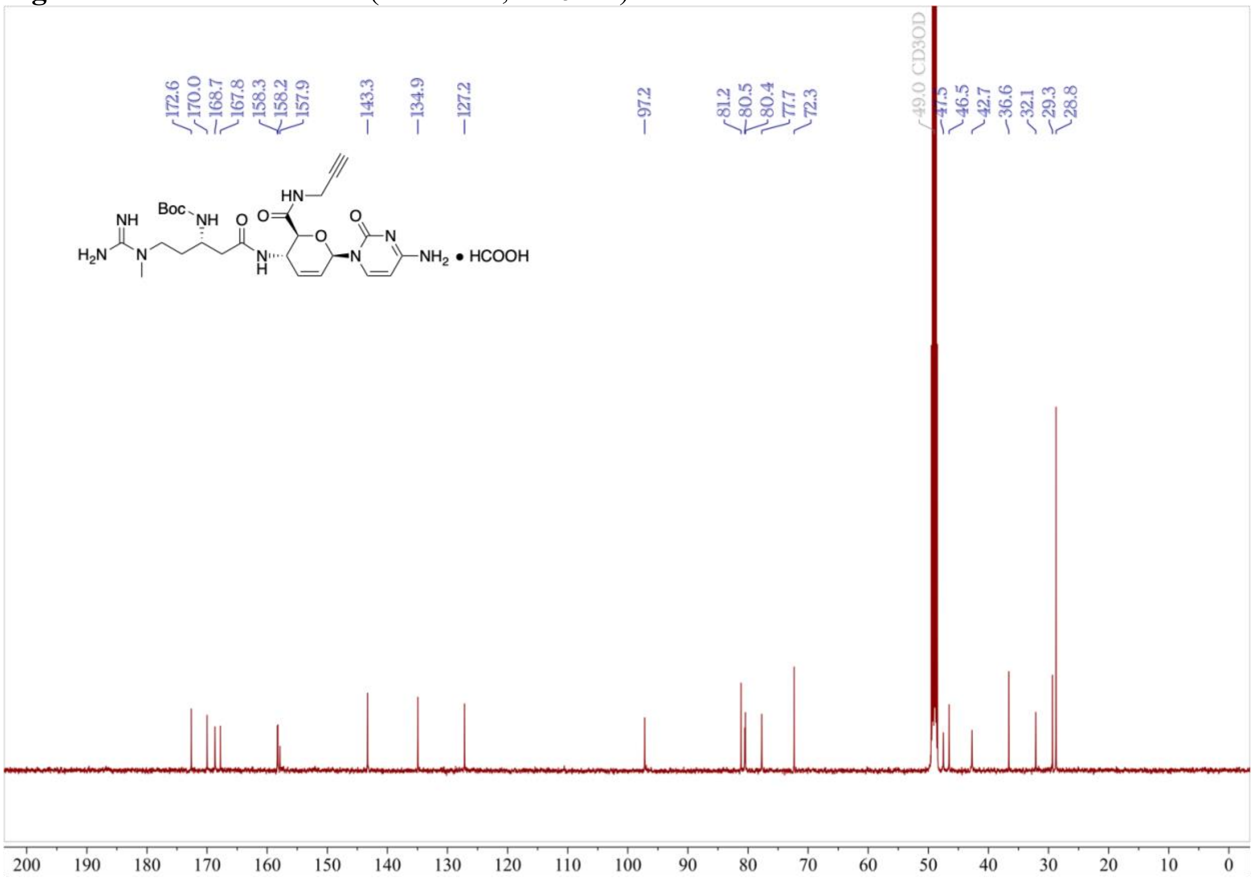

Figure S15. ^{1}H NMR of **12** (400 MHz, CD_3OD)

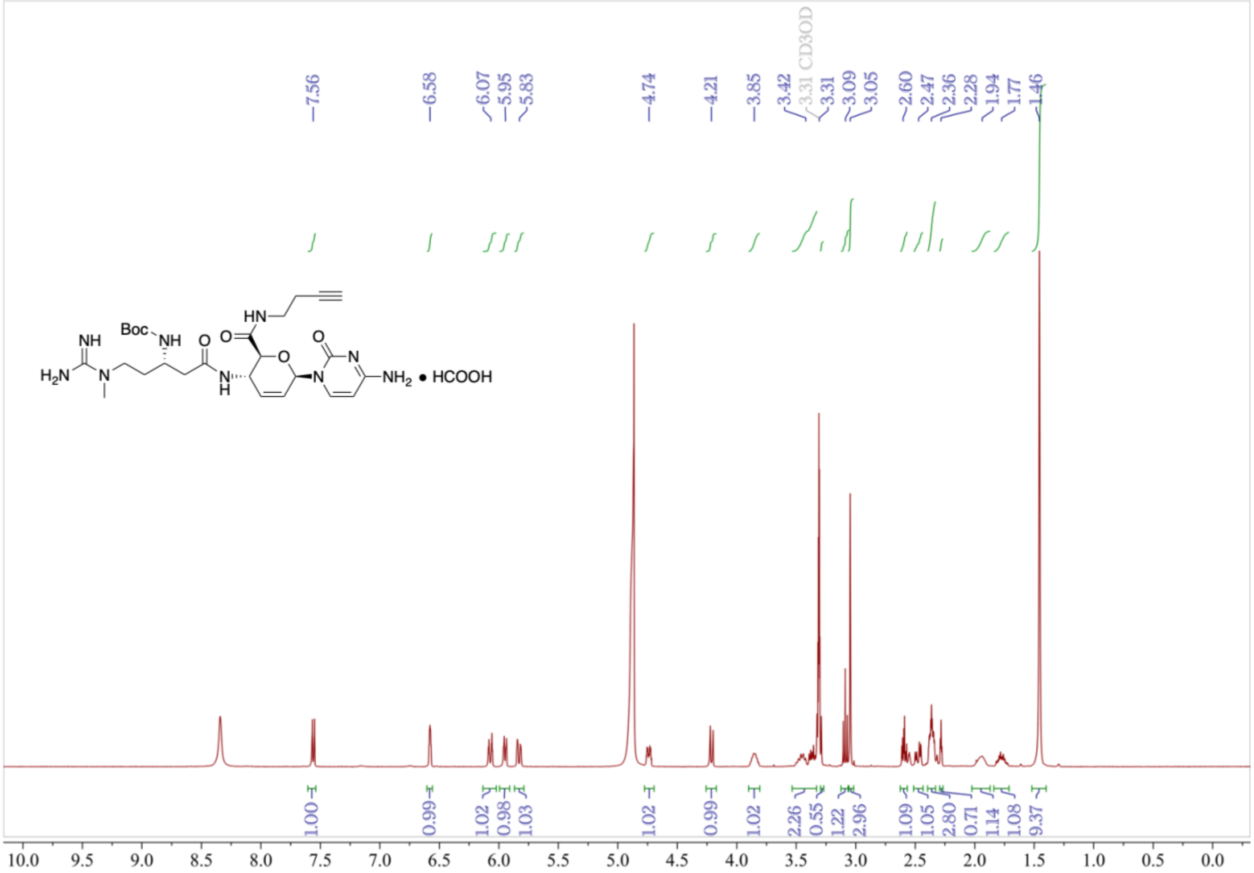

Figure S16. ^{13}C NMR of **12** (125 MHz, CD_3OD)

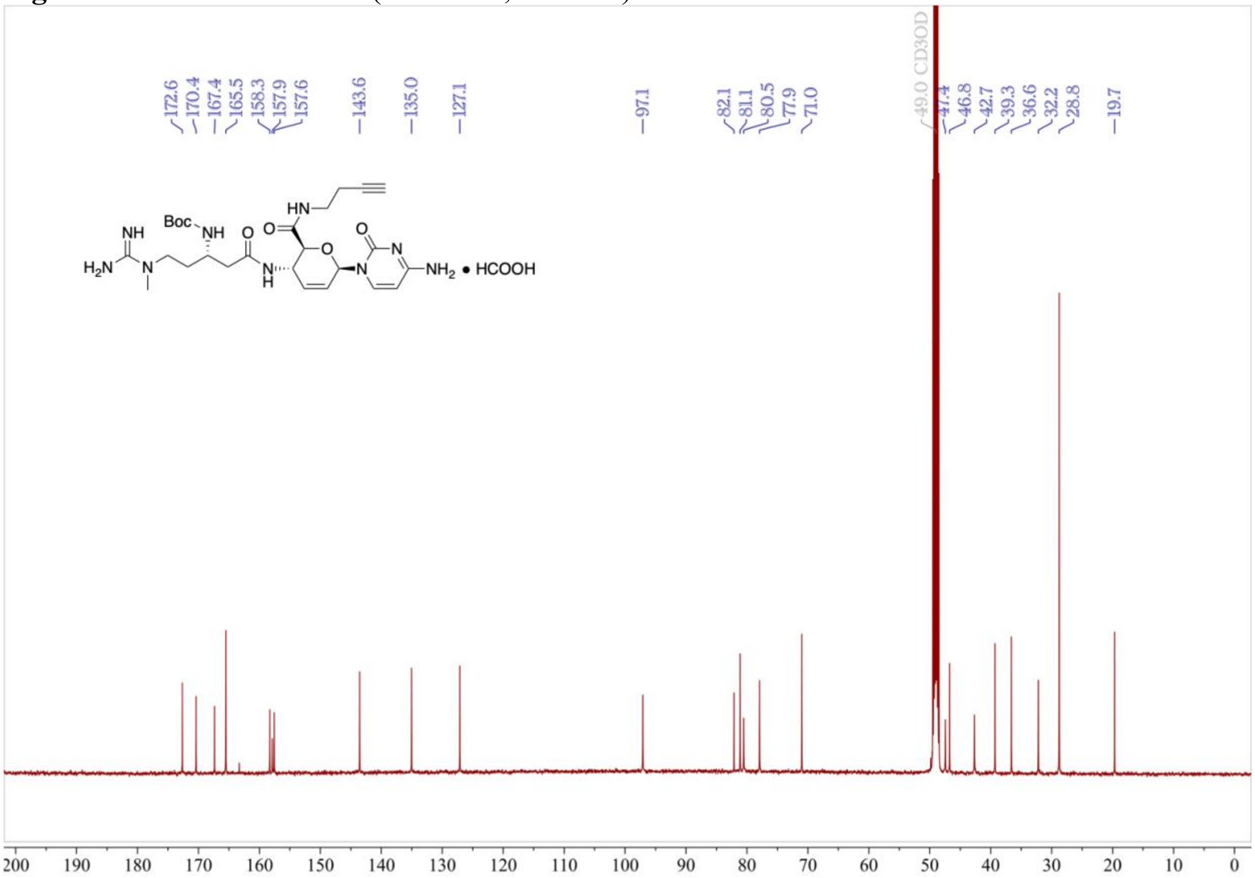

Figure S17. ^{1}H NMR of **13** (500 MHz, CD_3OD)

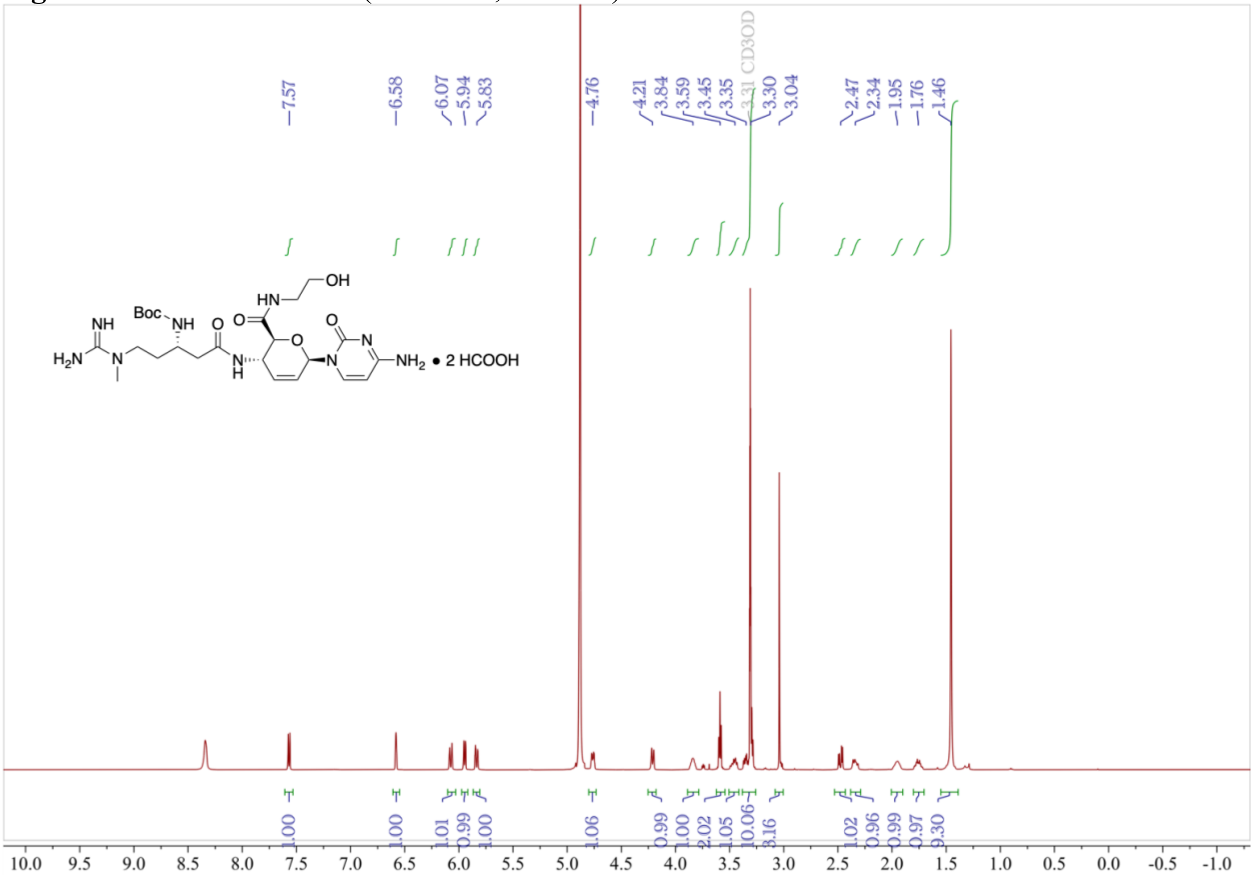

Figure S18. ^{13}C NMR of **13** (125 MHz, CD_3OD)

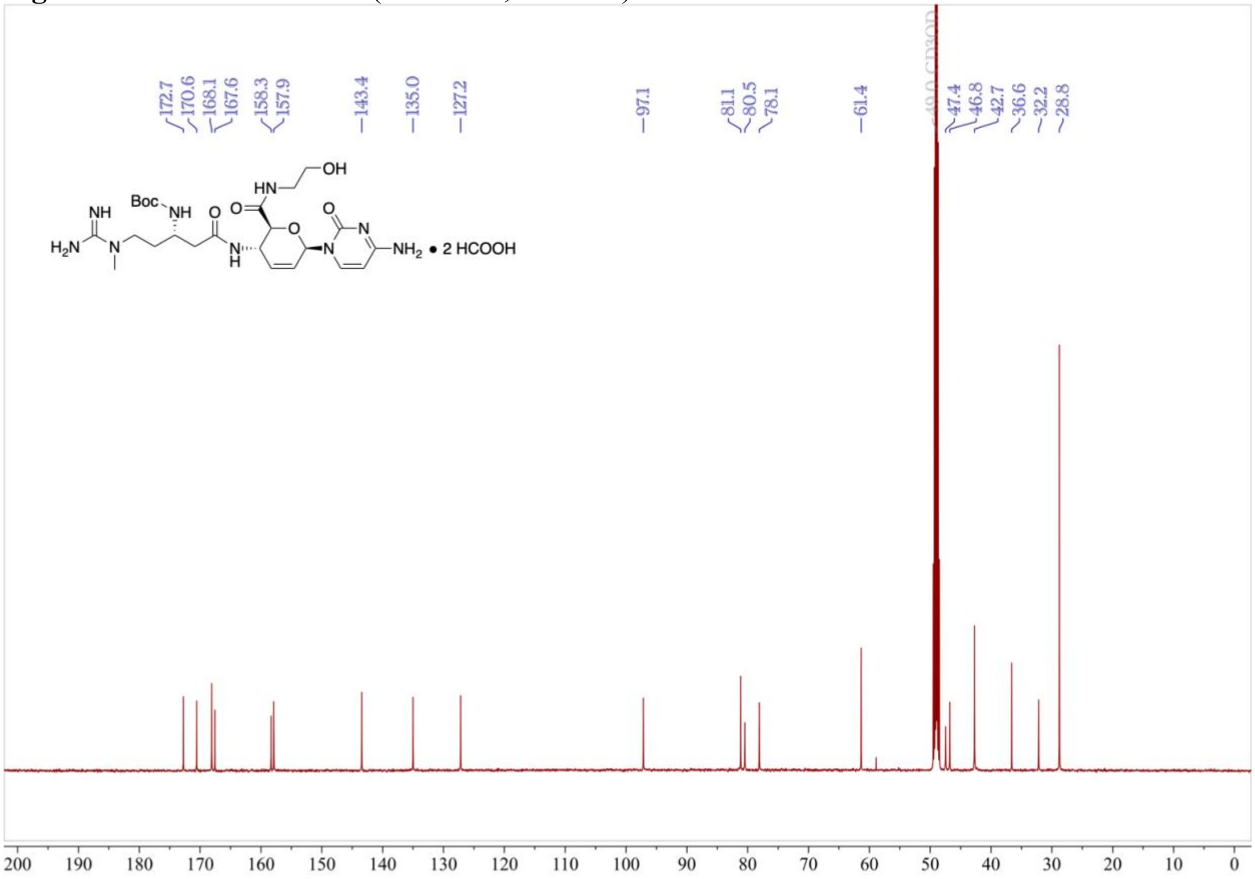

Figure S19. ^{1}H NMR of **14** (500 MHz, CD_3OD)

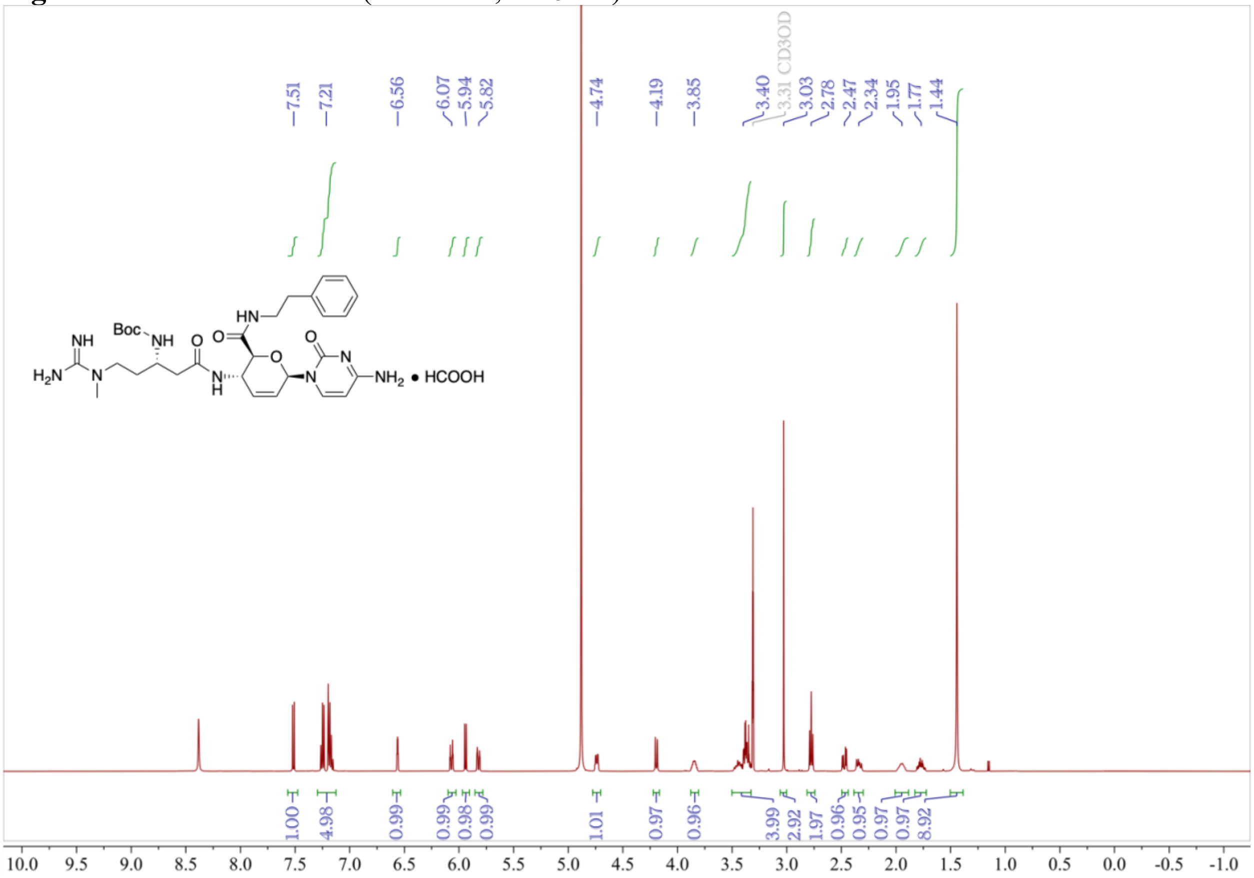

Figure S20. ^{13}C NMR of **14** (125 MHz, CD_3OD)

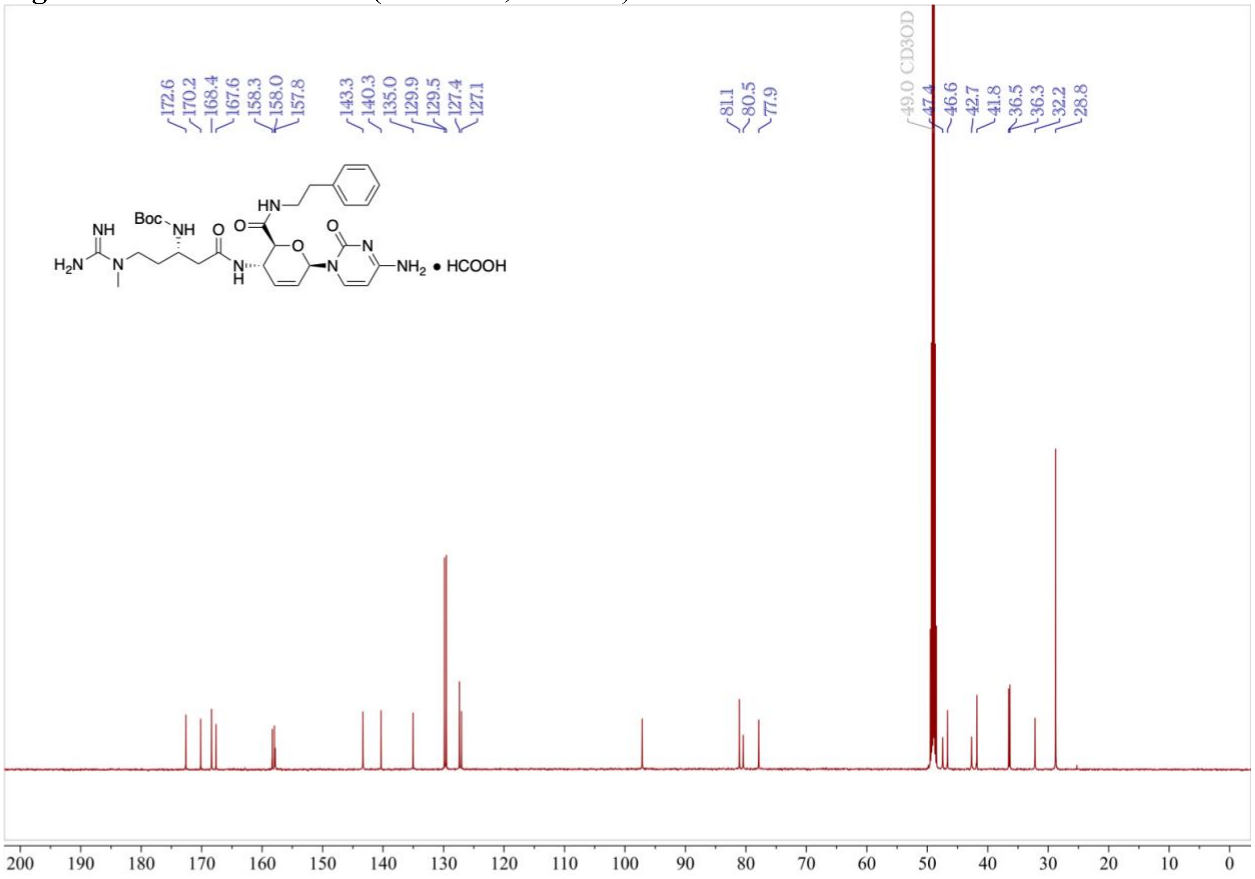

Figure S21. ^{1}H NMR of **15** (500 MHz, CD_3OD)

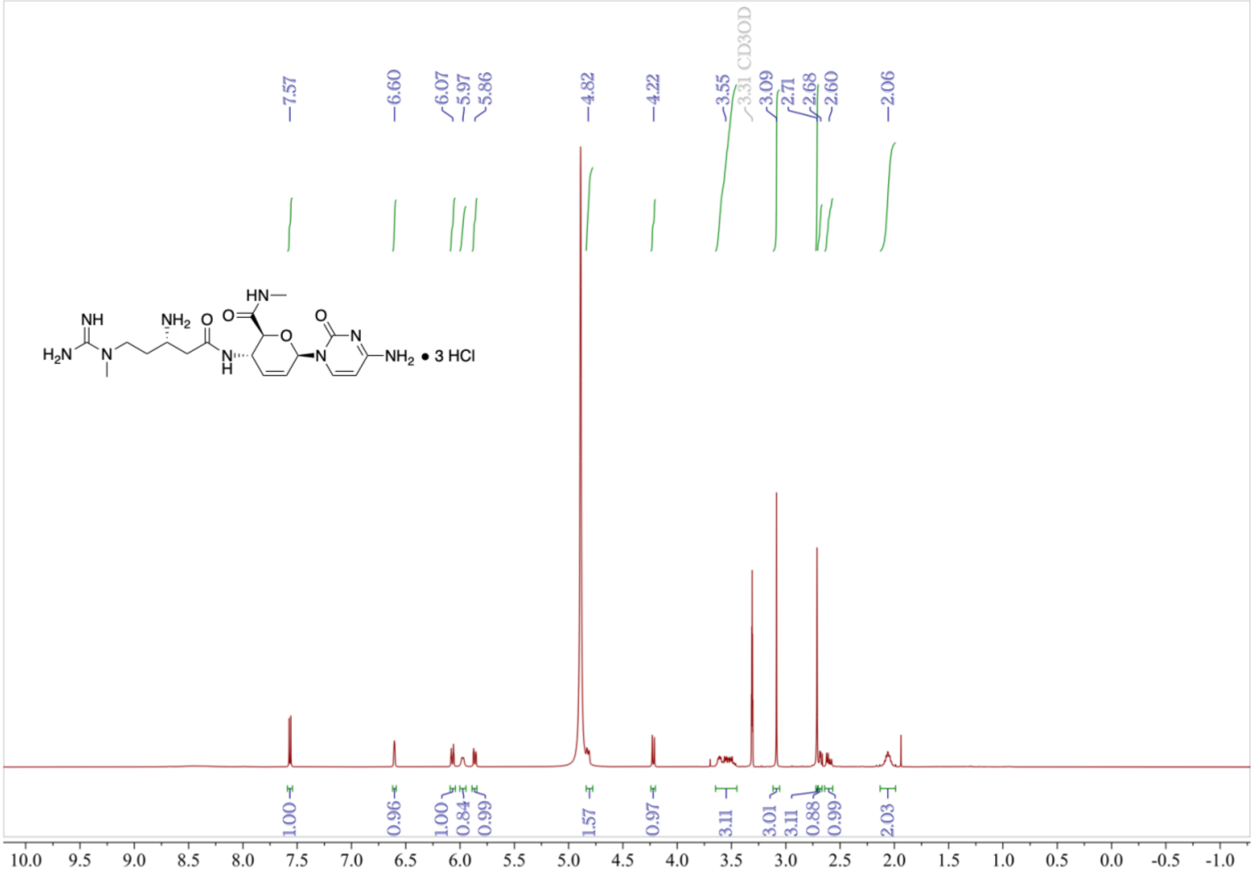

Figure S22. ^{13}C NMR of **15** (125 MHz, CD_3OD)

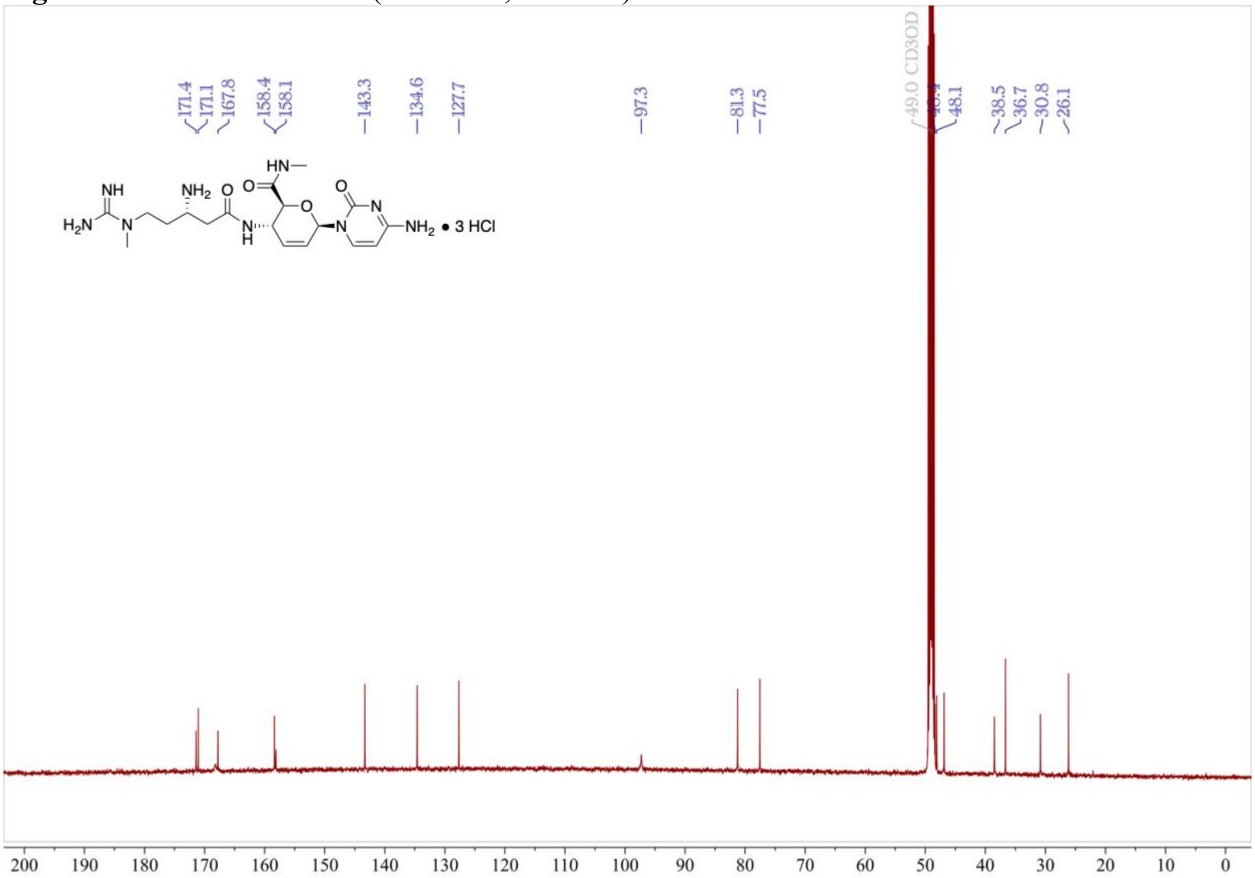

Figure S23. ^{1}H NMR of **16** (600 MHz, CD_3OD)

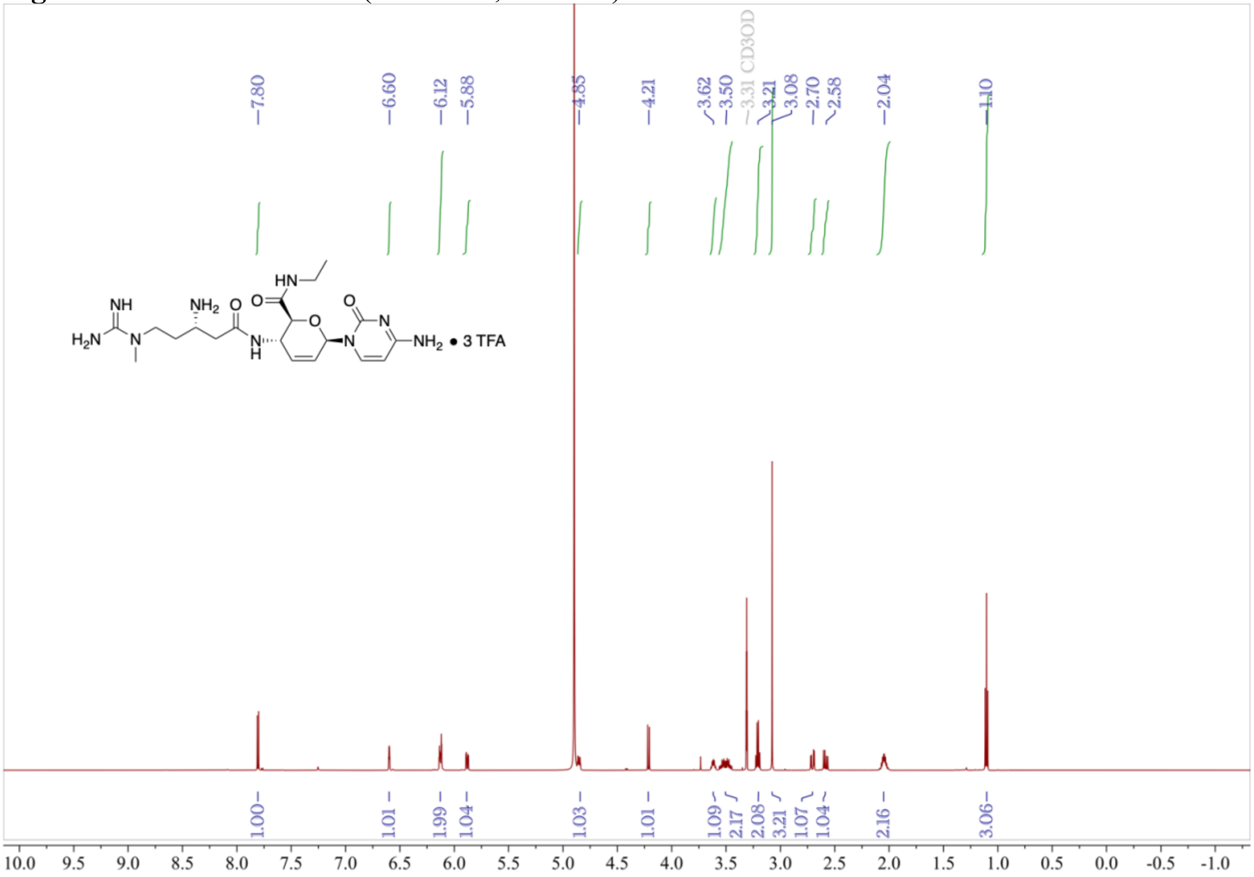

Figure S24. ^{13}C NMR of **16** (125 MHz, CD_3OD)

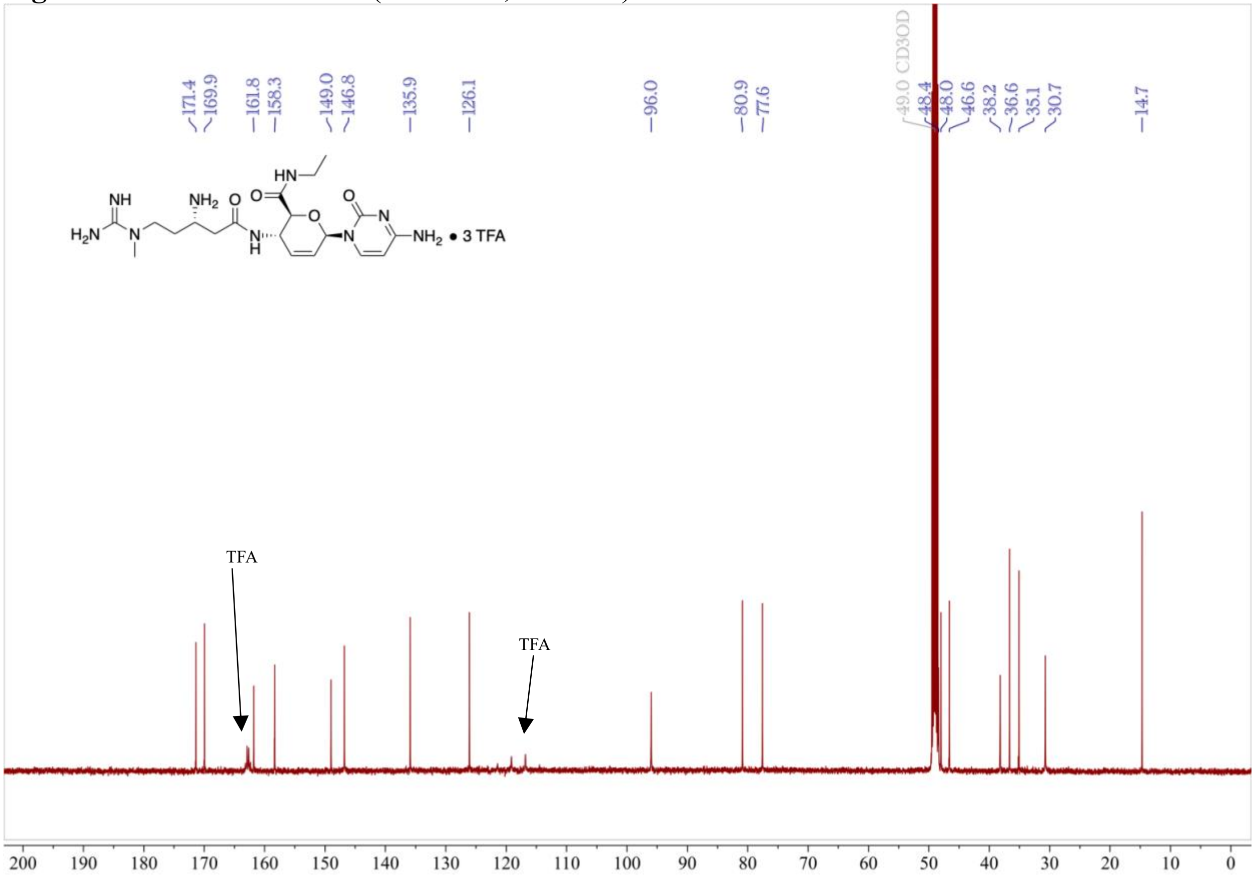

Figure S25. ^{1}H NMR of **17** (500 MHz, CD_3OD)

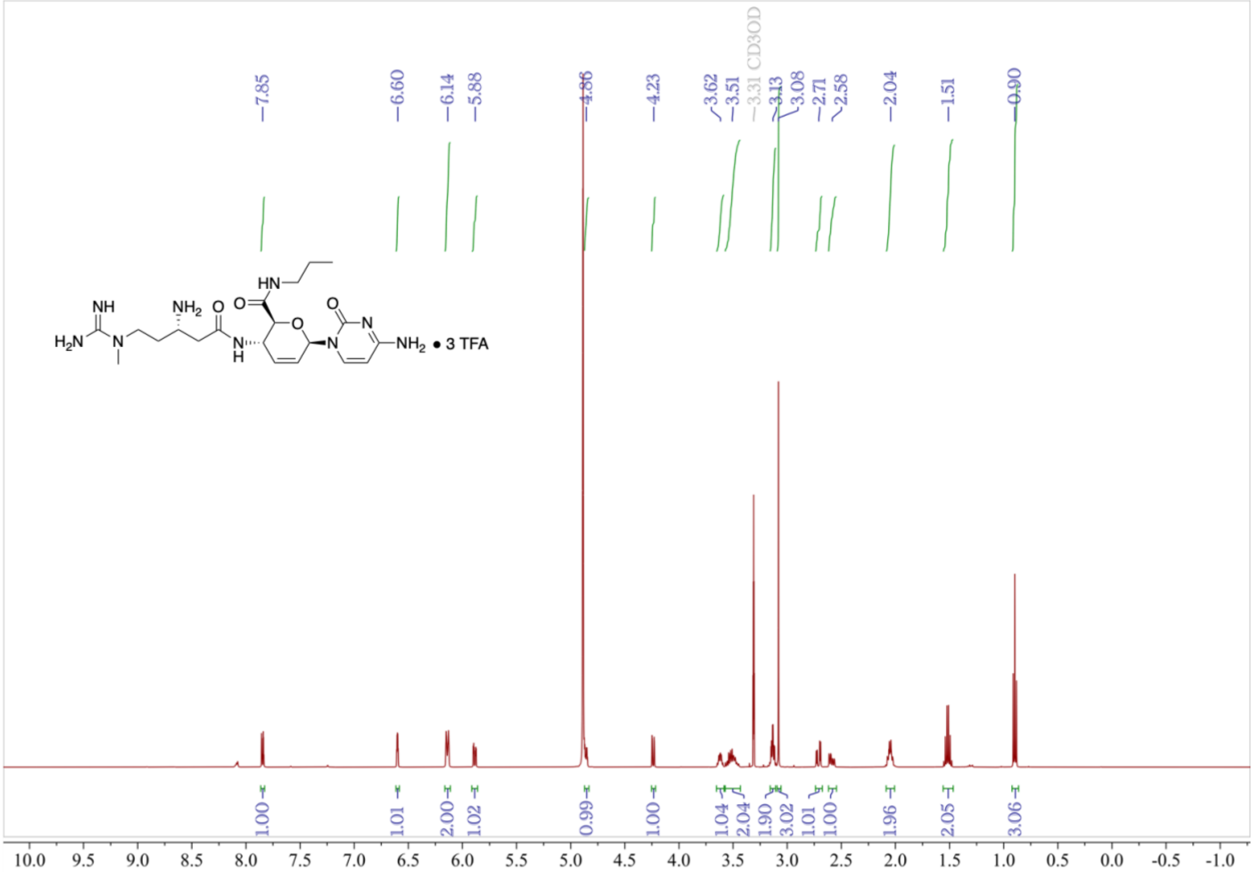

Figure S26. ^{13}C NMR of **17** (125 MHz, CD_3OD)

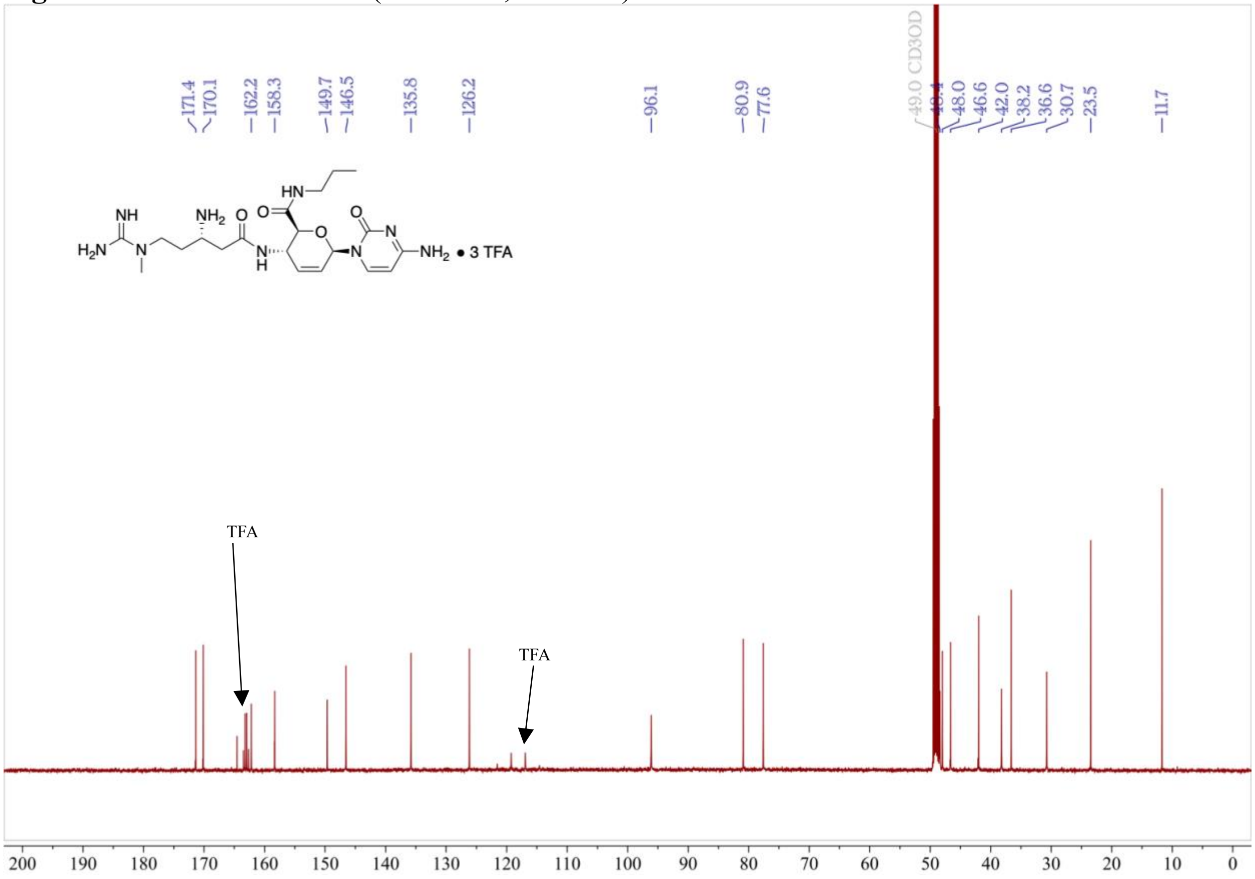

Figure S27. ^{1}H NMR of **18** (500 MHz, CD_3OD)

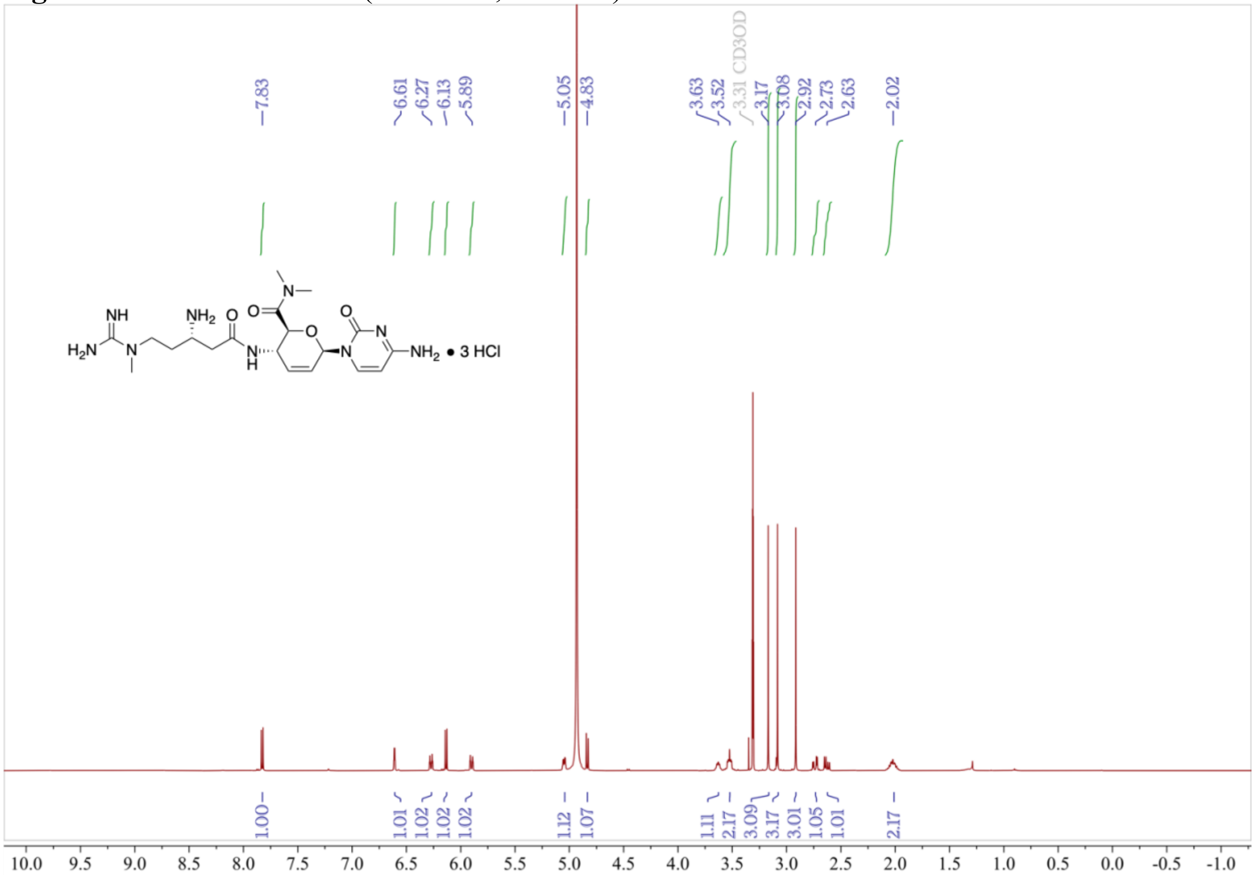

Figure S28. ^{13}C NMR of **18** (125 MHz, CD_3OD)

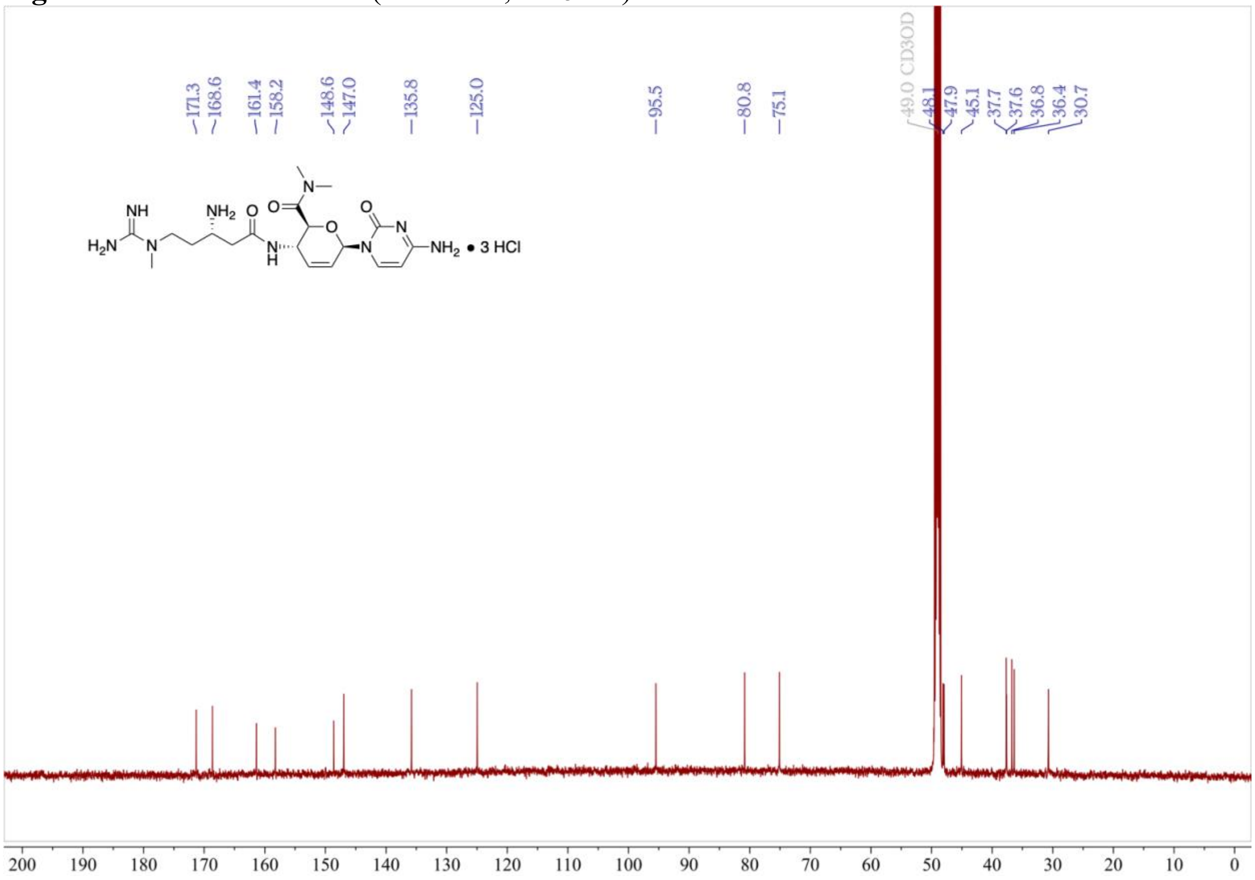

Figure S29. ^{1}H NMR of **19** + CH_3OH (500 MHz, CD_3OD)

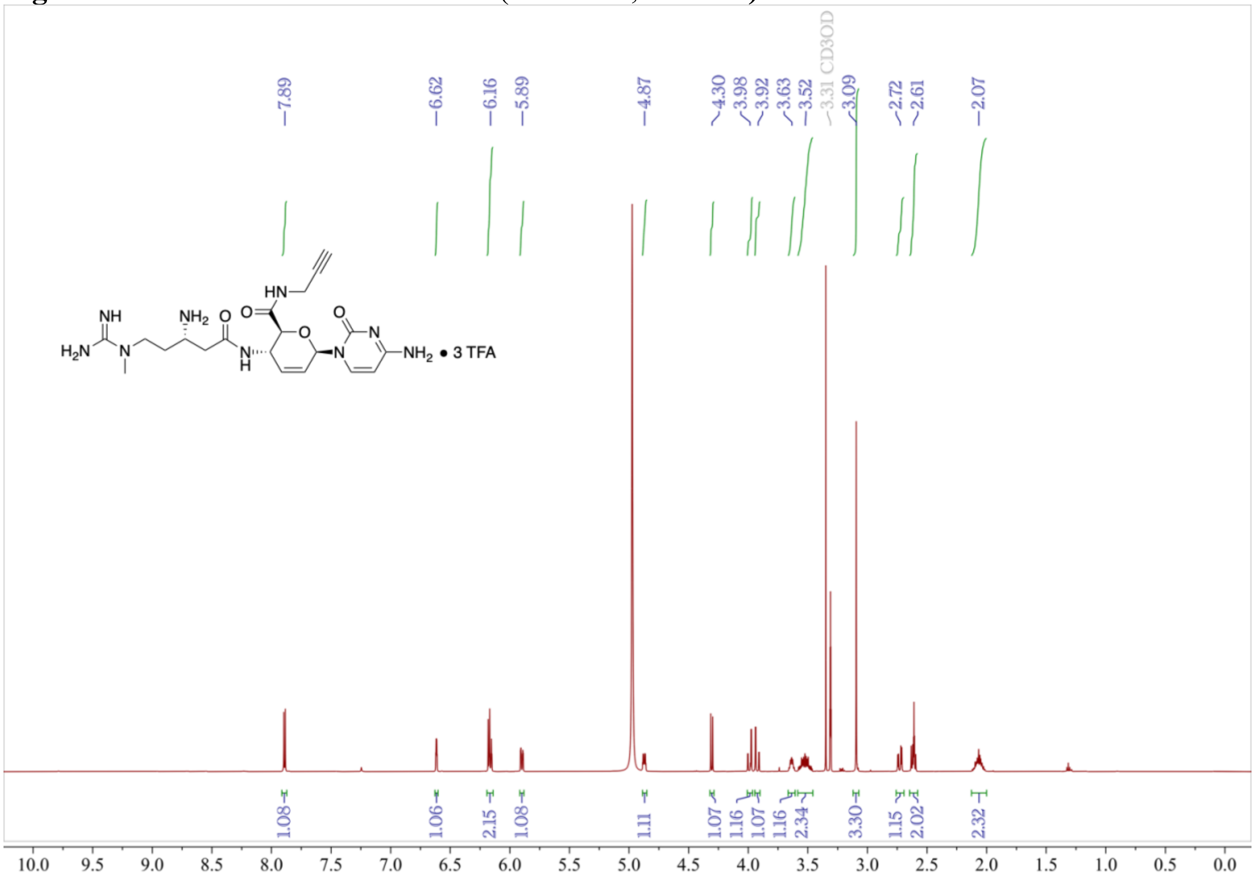

Figure S30. ^{13}C NMR of **19** (125 MHz, CD_3OD)

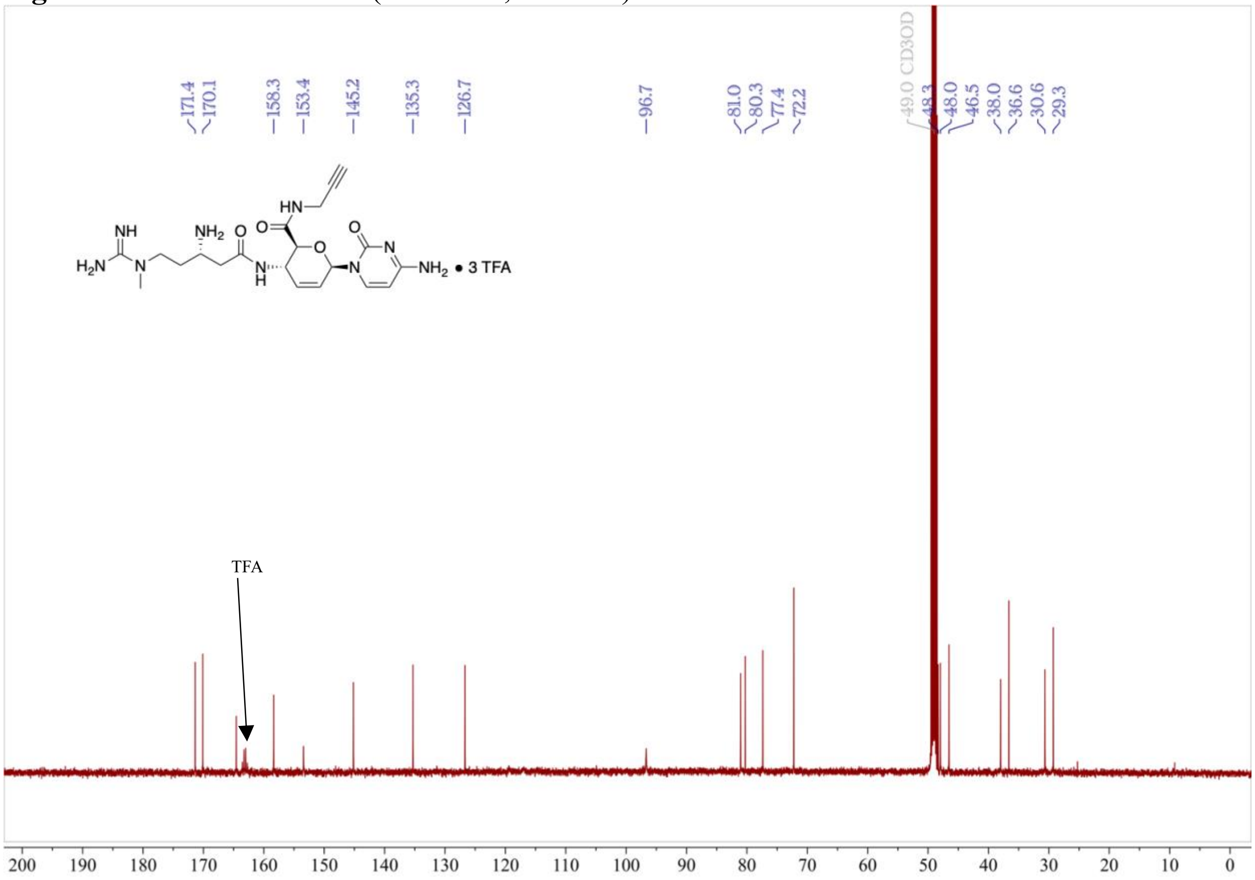

Figure S31. ^{1}H NMR of **20** (500 MHz, CD_3OD)

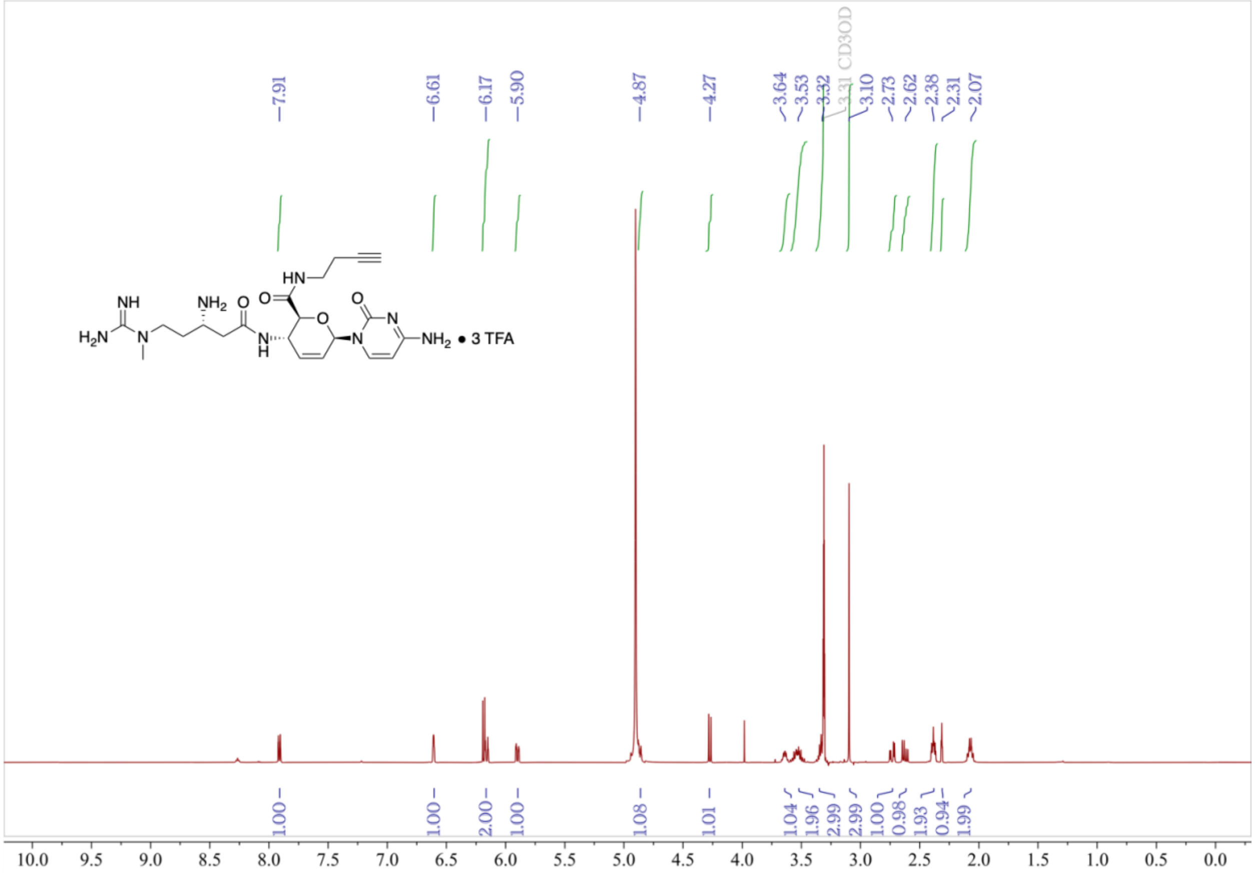

Figure S32. ^{13}C NMR of **20** (125 MHz, CD_3OD)

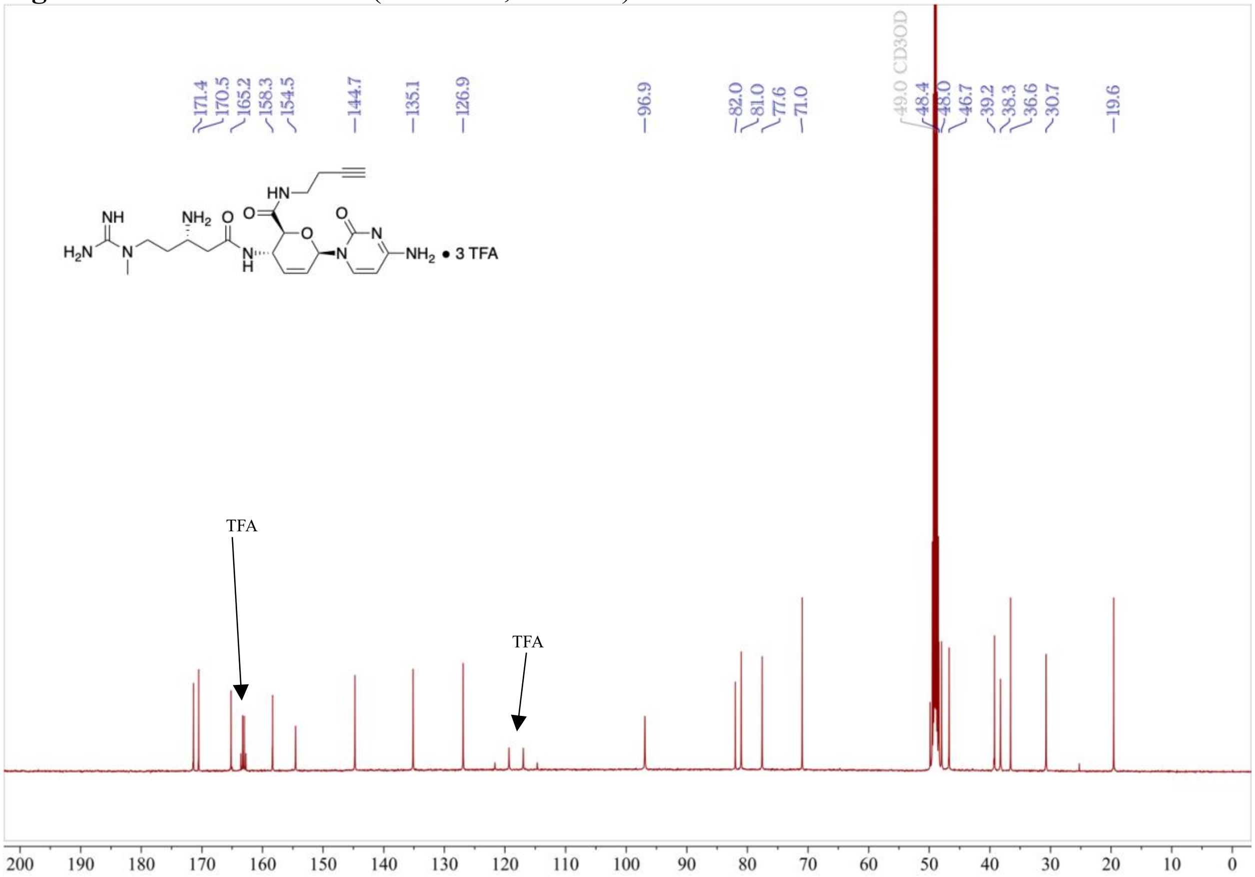

Figure S33. ^{1}H NMR of **21** (500 MHz, CD_3OD)

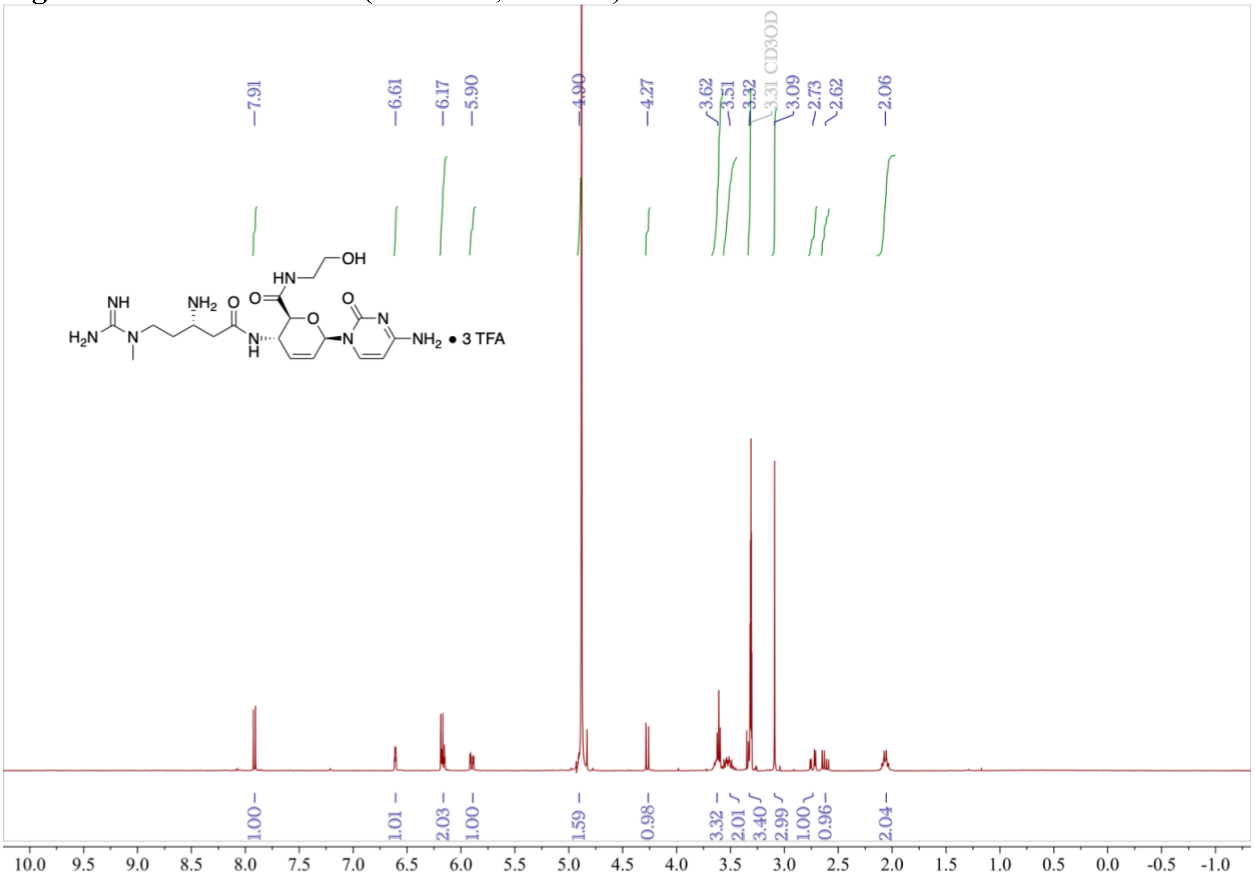

Figure S34. ^{13}C NMR of **21** (125 MHz, CD_3OD)

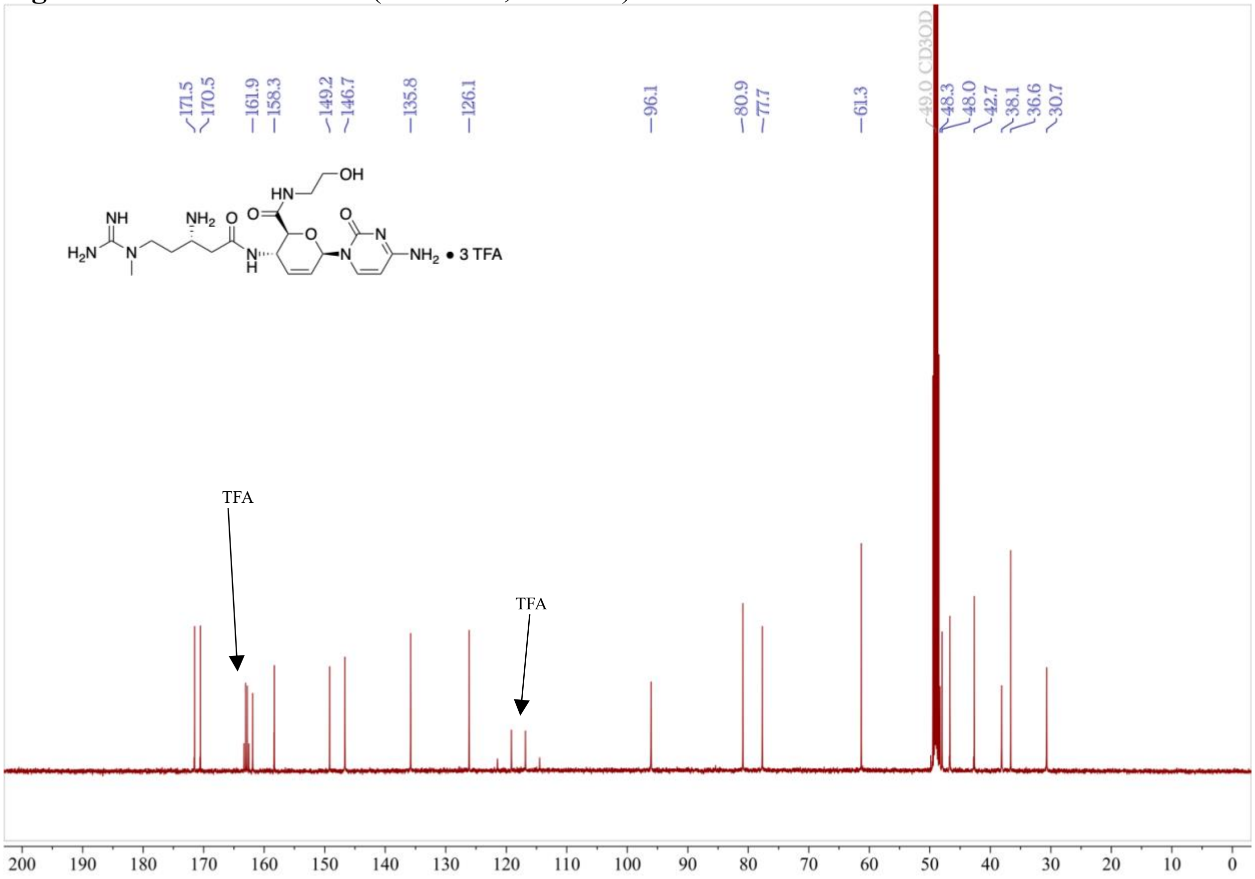

Figure S35. ^{1}H NMR of **22** (400 MHz, CD_3OD)

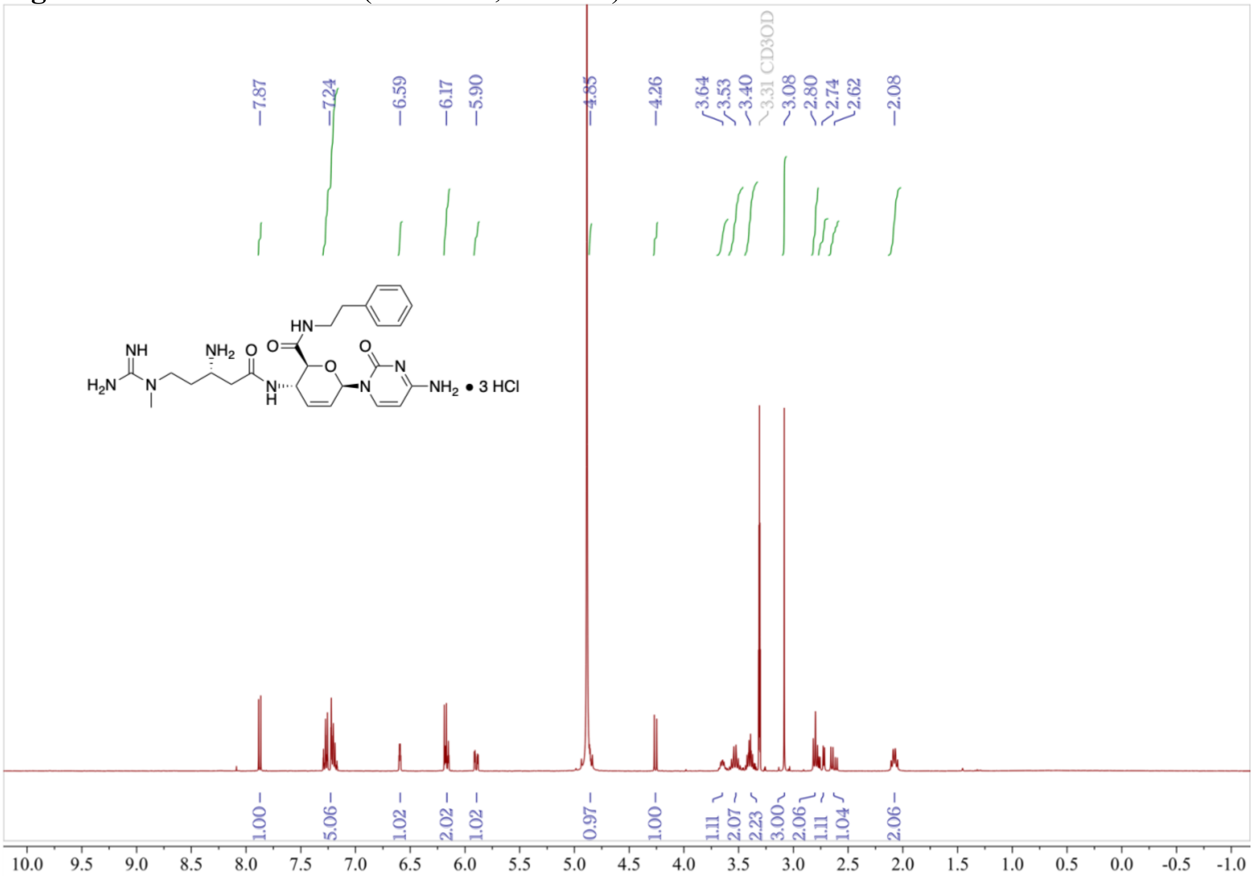

Figure S36. ^{13}C NMR of **22** (125 MHz, CD_3OD)

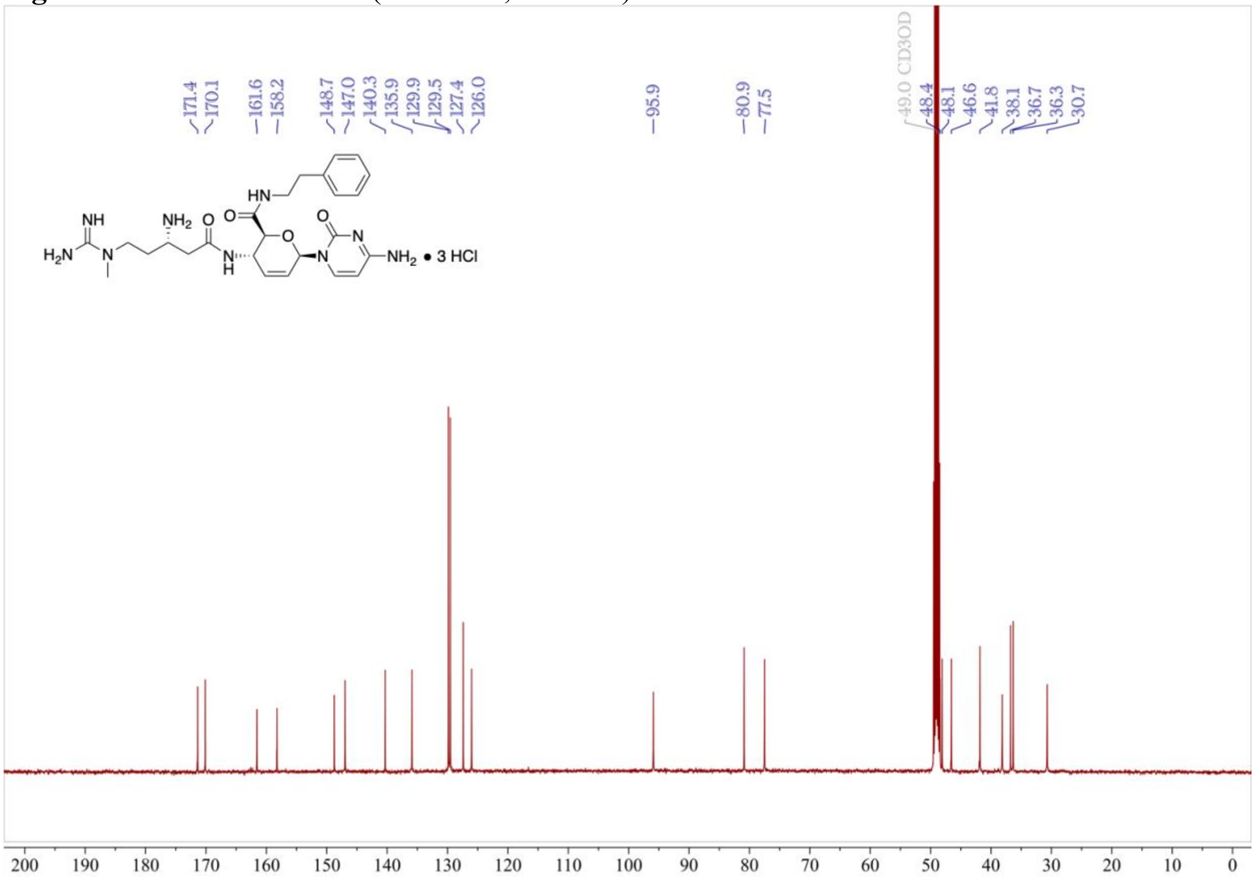

Representative HPLC traces for compounds 1, 2, and 22

Representative HPLC Traces for Compounds **1**, **2**, and **22** and vehicle/background trace

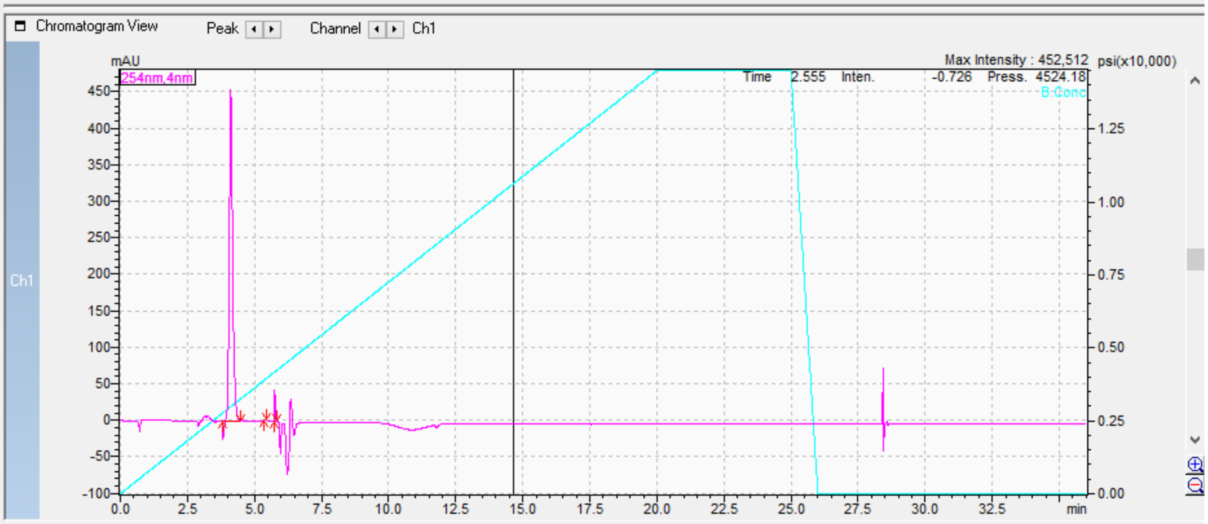

Results View - Peak Table

Peak Table | Compound | Group | Calibration Curve

Peak#	Ret. Time	Area	Height	Mark	Conc.	Unit	ID#	Name	Area%
1	4.112	3899671	454111	M	0.000				97.048
2	5.406	120	47	M	0.000				0.003
3	5.756	118485	41891	M	0.000				2.949
Total		4018276	496049		0.000				100.000

Extracted 190nm | **Ch1 254nm**

Figure S37. HPLC trace of commercial blasticidin S (**1**) with background subtraction.

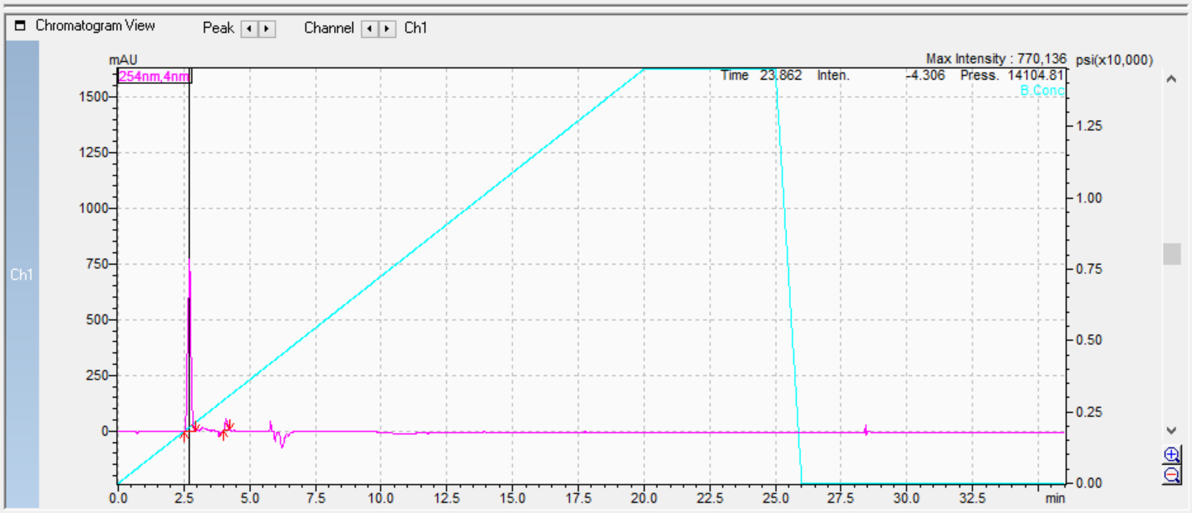

Results View - Peak Table

Peak Table | Compound | Group | Calibration Curve

Peak#	Ret. Time	Area	Height	Mark	Conc.	Unit	ID#	Name	Area%
1	2.712	6310533	770434	M	0.000				95.180
2	4.100	319561	47676	M	0.000				4.820
Total		6630094	818110		0.000				100.000

Extracted 190nm | **Ch1 254nm**

PDA Data A... | Data Comp...

Figure S38. HPLC trace of **2** with background subtraction.

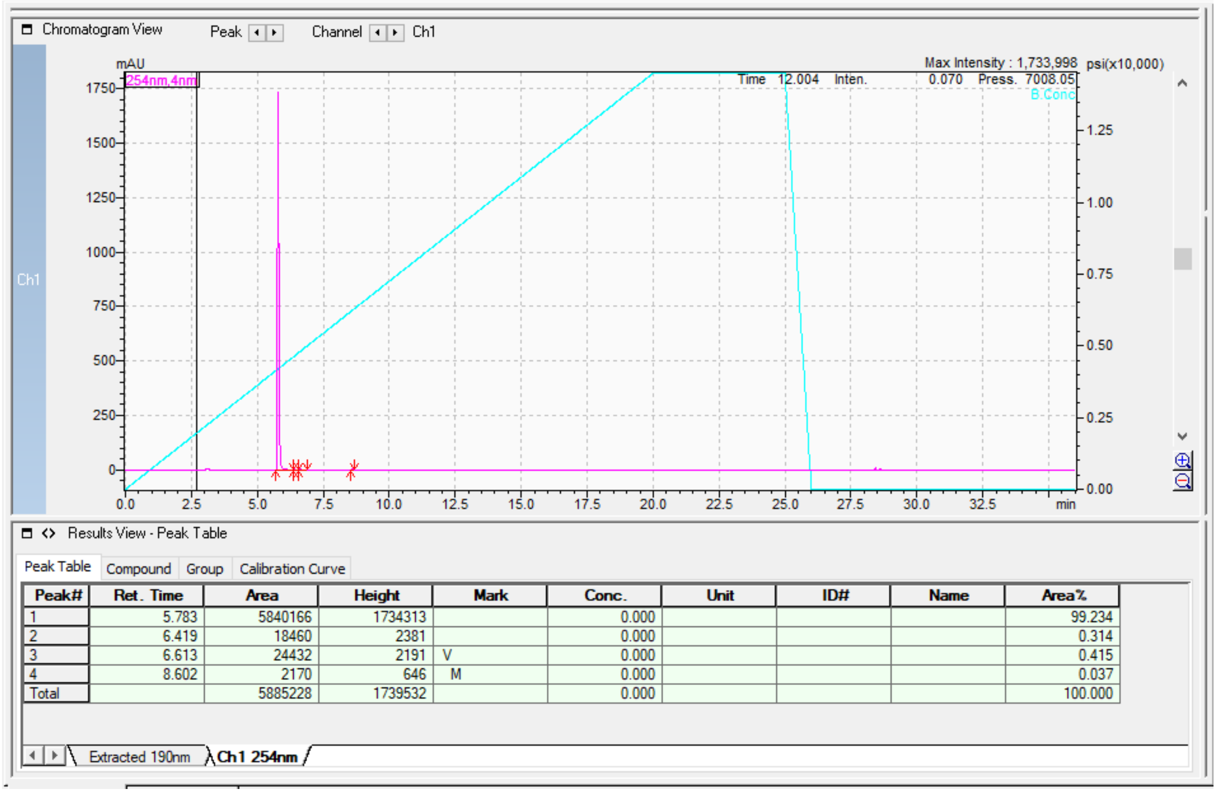

Peak#	Ret. Time	Area	Height	Mark	Conc.	Unit	ID#	Name	Area%
1	5.783	5840166	1734313		0.000				99.234
2	6.419	18460	2381		0.000				0.314
3	6.613	24432	2191	V	0.000				0.415
4	8.602	2170	646	M	0.000				0.037
Total		5885228	1739532		0.000				100.000

Figure S39. HPLC trace of **22** with background subtraction.

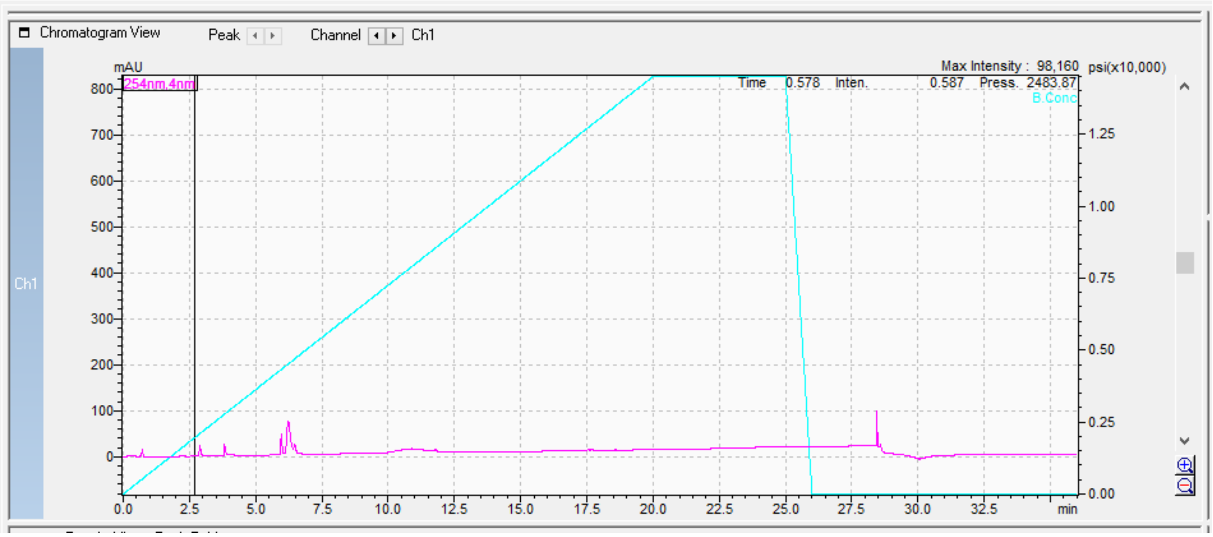

Figure S40. HPLC trace of the vehicle blank. Peaks present are column/equipment peaks.

Antibacterial Assay Procedure

Broth Microdilution Assays

Media and solutions were autoclaved or sterile filtered prior to use and manipulations were carried out in a laminar flow hood. Antibacterial testing was performed in polypropylene 96-well flat bottom plates. The minimal inhibitory concentration of blasticidin S (**1**) hydrochloride, P10 (**2**), and derivatives **15**–**22** were assessed against *Staphylococcus aureus* ΔNorA, *S. aureus* 8810, methicillin-resistant *S. aureus* (MRSA) ATCC 43300, *Enterococcus faecalis* ATCC 29212, vancomycin-resistant *Enterococcus*, *Klebsiella pneumoniae* ATCC 29665, *Pseudomonas aeruginosa*, and *Acinetobacter baumannii* ATCC 17978 using serial dilutions. Doxycycline (Gold Bio) as a positive control and the vehicle as a negative control. Stock solutions of each compound were prepared at 5.12 mg/mL in 50% DMSO/water and serially diluted in 50% DMSO/water to create master plates. From the master plates, 10 µL of each dilution was applied to test plates using a Bravo automated liquid transfer system (Agilent). Bacteria previously grown overnight (37 °C, 120 – 200 RPM), in LB broth were diluted to an OD600 of 0.05 in cation-adjusted Mueller-Hinton broth, grown to an OD600 of ~0.5, and diluted to an OD600 of 0.004 in cation-adjusted Mueller-Hinton broth. These diluted cultures were applied to the test plates (190 µL per test well) resulting in final concentrations of the test compounds of 256–2 µg/mL. Growth controls (wells with inoculated media but no vehicle or test compounds) were included to assess any potential toxicity from the vehicle. The plates were incubated at 37 °C for 16–18 h, except for VRE and *E. faecalis*, which were incubated at 37 °C for 24 h. Inhibition was determined by measuring the optical density at 600 nm (OD600) with a Cytation 3 plate reader (BioTek). The optical density measurements were normalized to the positive and negative controls and the vehicle and growth controls were compared and found to be indistinguishable, indicating no toxicity from the vehicle used. The MIC was the lowest concentration that inhibited >90% of growth. Two biological replicates were performed in duplicate. MIC values are reported

as a range from biological replicates. Inhibition curves were generated using Prism (GraphPad). The IC_{50} values reported in **Table 2** are the average of the two biological replicates ± standard deviation.

<u>Cytotoxicity Assay Procedure</u>

Human lung fibroblast cells, MRC-5 (CCL-171), were cultured at 37°C with 5% CO2 and were plated at 5000 cells/well in a 96-well plate. Compounds 1, 2, and 15-22 were initially prepared at concentrations of 5.12 mg/mL in a vehicle of 50% DMSO. Dilutions of the compounds (4 – 512 µg/mL) were then prepared in growth medium containing Dulbecco's modified Eagle's medium (DMEM) with high glucose, L-glutamine, and sodium pyruvate supplemented with 10% fetal bovine serum (FBS), 1% non-essential amino acids, and 0.1% gentamicin sulfate, and 25mM HEPES. The vehicle (50% DMSO) was also prepared via the same dilution series to serve as a control. Growth media was removed from the cells and replaced with the same volume of each compound dilution. Cells were incubated for 24 h, after which CellTiter 96® AQueous One Solution Reagent was added to each well as recommended by the manufacturer's protocol (Promega CellTiter 96 AQueous One Solution Cell Proliferation Assay) and further incubated for 4 h. After incubation, absorbance was measured at 490 nm on an Infinite M plex multimode microplate reader with i-control Tecan software. Viability was calculated by subtracting media only absorbance values and normalizing to the vehicle control at each concentration. The presented data is one assay performed in triplicate that is representative of multiple trials. Data for the 256 and 512 µg/mL concentrations were removed from analysis due to vehicle toxicity displayed at these concentrations. CC50 concentrations were determined via the ED50 Plus v1.0 software (https://archive.org/details/ed50v10_zip).

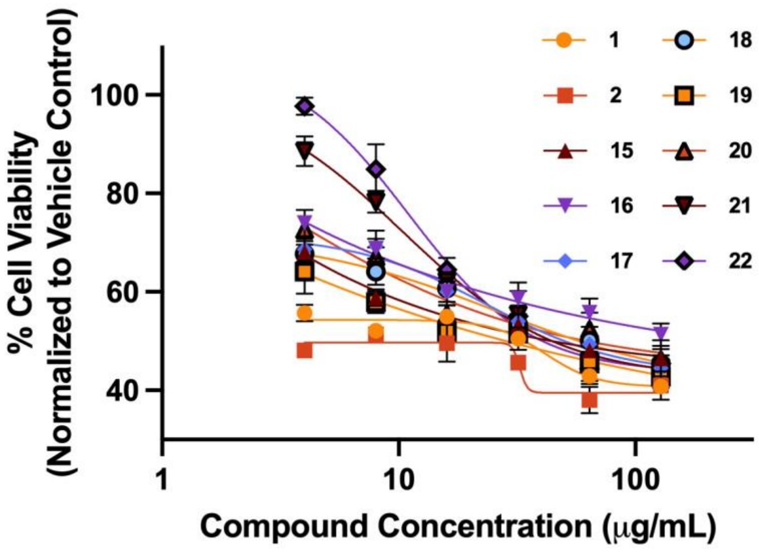

Figure S41. The effect of compounds on cell viability. The lines represent non-linear regression curves, and error bars represent means ± SD (n=3). Cell viability concentrations between 4 - 128 μg/mL are displayed.

Molecular Docking Information

Computational studies were performed using a 70S *Thermus thermophilus* ribosome with *Escherichia coli* release factor 1 (RF1) crystal structure (PDB ID: 6B4V)[2] retrieved from RCSB PDB. The *T. thermophilus* ribosome was in complex with a co-crystalized blasticidin S ligand. PyMOL 2.5.0 (Schrodinger, LLC. The PyMOL Molecular Graphics System, Version 1.8. 2015.) was used to prepare the ribosome for molecular docking. Small molecules, waters, and ions were removed from the environment. The crystal structure was parsed to include all structural elements of the large 50S ribosomal subunit within 15 Å of the co-crystalized blasticidin S binding site. The co-crystalized blasticidin S ligand was separated from the ribonucleoprotein complex, followed by export of each object as PDB files with relative atom coordinates preserved. Blasticidin S derivatives were designed in a 2D format using ChemDraw, accounting for the expecting protonation state under physiological conditions. Chem3D was used to convert the derivatives to 3D PDB files, which were further refined using OPLS4 energy minimization in Schrodinger-Maestro. AutoDock Tools 1.5.6[3] was used to prepare the structure and ligand files by adding polar hydrogens, determining ligand torsion, computing Gasteiger partial charges, and

assigning AD4 atom types. Molecular redocking was performed with AutoDock Vina[4] using a box size of 30 Å x 30 Å x 30 Å centered on the coordinates 60.307, 130.797, 107.596, which is based on the blasticidin S ligand position in the *T. thermophilus* ribosome. Previous work has established this box size as suitable for unbiased redocking to validate ribosome crystal structures[5]. Redocking resulted in ligand poses with a root mean square deviation (RMSD) value of less than 2 Å (**Table S1**), establishing that the processed ribosome structure from PDB ID: 6B4V can reliably reflect real-world binding poses in computational studies.

All derivatives, and a blasticidin S structure prepared identically to the derivatives, were docked using a box size of 20 Å x 20 Å x 20 Å centered on the previously mentioned coordinates. The box size was selected specifically to accommodate the blasticidin S derivatives so that they would have ample room to sample a vast amount of conformations while remaining near the experimentally known target site to minimize off target poses. Interaction fingerprinting from Schrodinger-Maestro 2021-28 (Schrödinger, LLC. Maestro. 2021-2 ed.; New York, NY, 2021) was used to determine the frequency of docked ligand interactions with specific RNA nucleotides in the ribosome structure. The interaction fingerprint for blasticidin S was made using the docking results from the 20 Å x 20 Å x 20 Å dock.

Table S1. RMSD and predicted binding affinity from redocking blasticidin S in a *Thermus thermophilus* large 50S ribosomal subunit with *Escherichia coli* RF1 (PDB ID: 6B4V).

Pose	RMSD (Å)	Binding Affinity (kcal/mol)
1	3.43	-8.9
2	3.81	-8.9
3	3.34	-8.8
4	**1.29**	-8.7
5	3.52	-8.4
6	9.18	-8.4
7	11.0	-8.2
8	8.48	-8.2
9	10.2	-8.1
AVG	6.02	-8.5

Table S2. RMSD and predicted binding affinity from redocking blasticidin S in a *Thermus thermophilus* large 50S ribosomal subunit (PDB ID: 4V9Q).[6]

Pose	RMSD (Å)	Binding Affinity (kcal/mol)
1	13.0	-9.1
2	**1.78**	-9.0
3	9.03	-9.0
4	3.81	-8.9
5	10.3	-8.8
6	12.8	-8.8
7	5.73	-8.7
8	7.47	-8.7
9	3.46	-8.7
AVG	7.48	-8.9

Figures S42-S52. Lowest energy docking poses for all compounds overlaid with crystal BLS in a *Thermus thermophilus* large 50S ribosomal subunit with *Escherichia coli* RF1 (PDB ID: 6B4V).

Crystal structure BLS redock

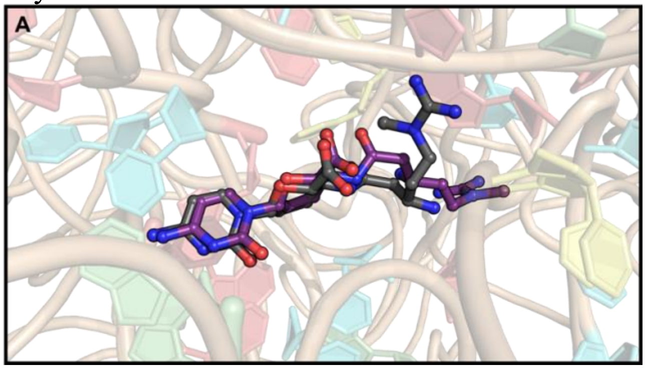

BLS (**1**)

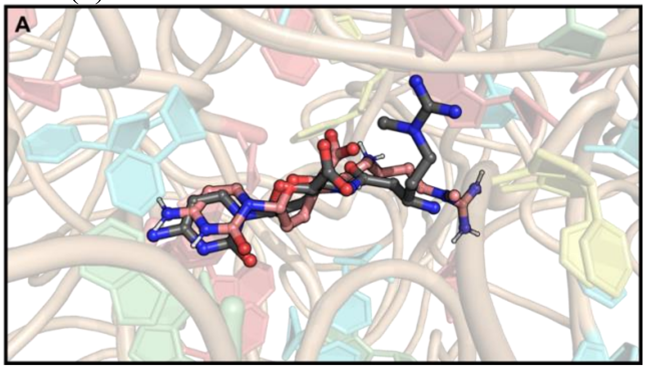

P10 (**2**)

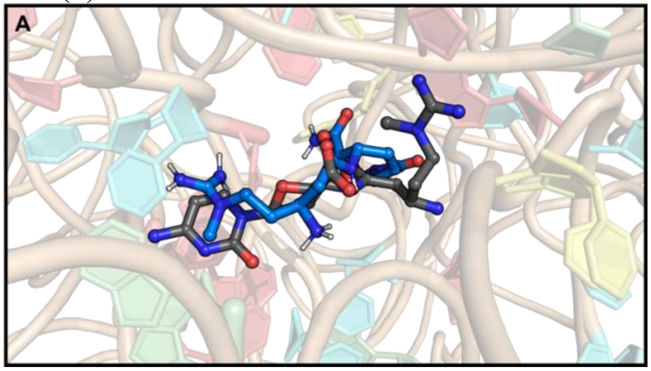

Methyl amide (**15**)

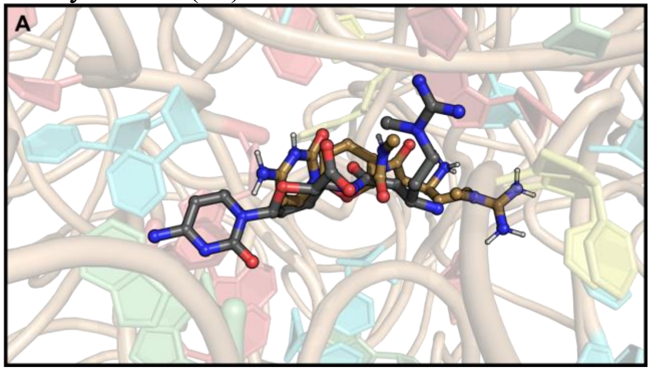

Ethyl amide (**16**)

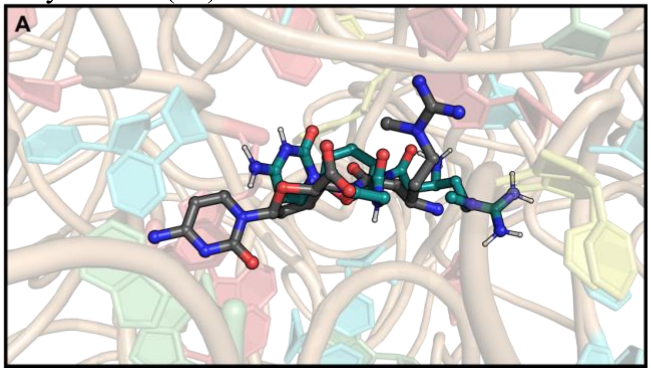

Propyl amide (**17**)

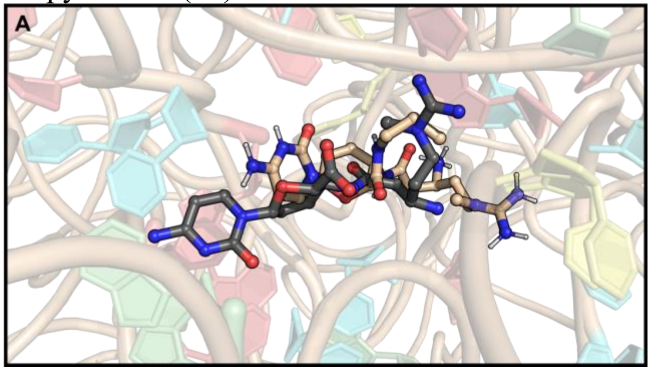

Dimethyl amide (**18**)

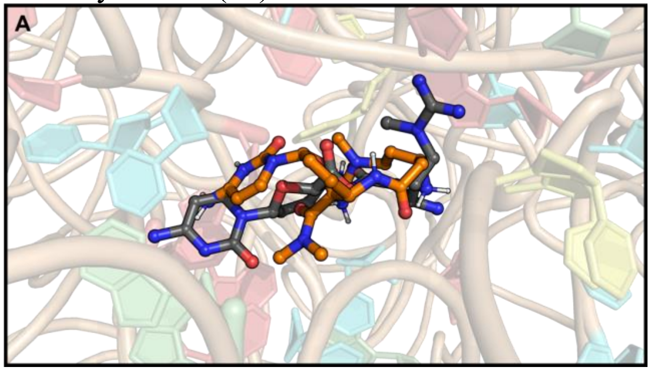

Propargyl amide (**19**)

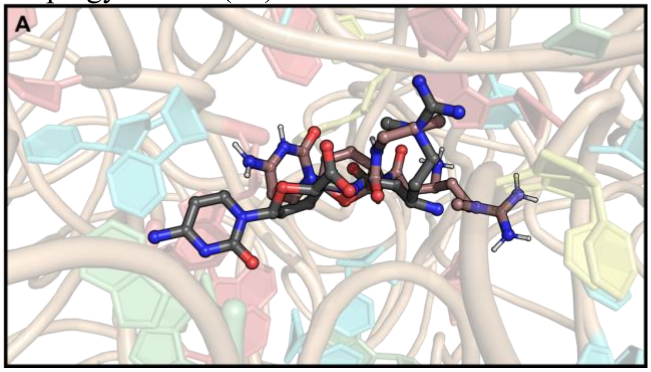

3-Butynyl amide (**20**)

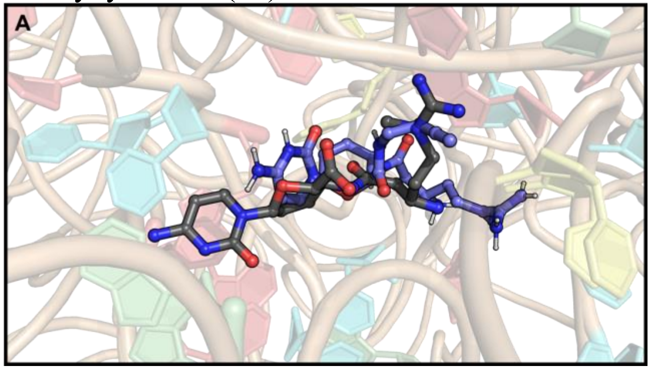

Ethanol amide (**21**)

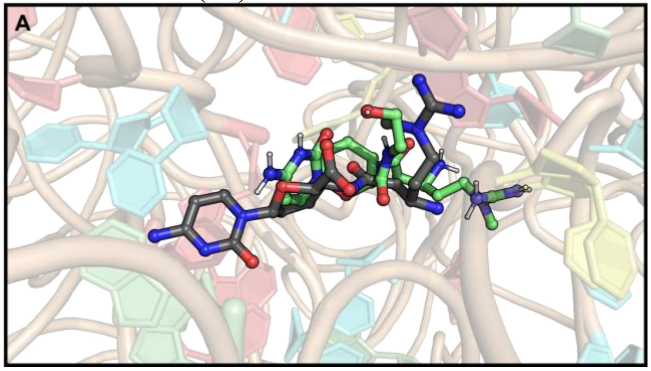

Phenethyl amide (**22**)

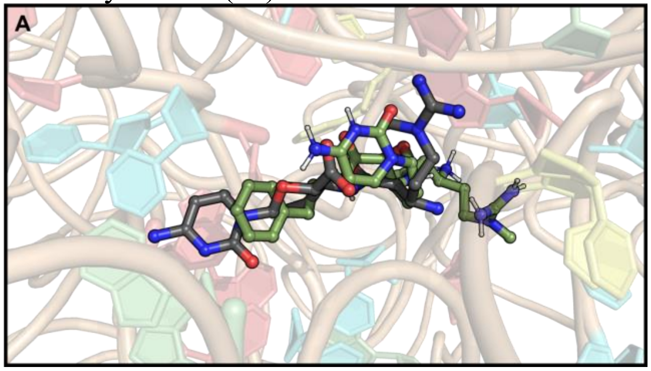

Property Data for Compounds 1, 2, 15-22

Table S3. Selected Computed Property Data for Compounds

All property data was generated by the SwissADME web tool[7] and is sorted according to the Silicos-IT log P value (green highlight). The average MIC for the Gram-positive bacteria minus the *S. aureus* NorA knockout was added to the data.

Compound Number	Formula	MW	#Heavy atoms	#Aromatic heavy atoms	Fraction Csp3	#Rotatable bonds
**2**	C17H29N9O4++	423.47	30	6	0.47	10
**1**	C17H27N8O5+	423.45	30	6	0.47	10
**21**	C19H33N9O5++	467.52	33	6	0.53	13
**18**	C19H33N9O4++	451.52	32	6	0.53	11
**15**	C18H31N9O4++	437.5	31	6	0.5	11
**16**	C19H33N9O4++	451.52	32	6	0.53	12
**19**	C20H31N9O4++	461.52	33	6	0.45	12
**17**	C20H35N9O4++	465.55	33	6	0.55	13
**20**	C21H33N9O4++	475.54	34	6	0.48	13
**22**	C25H37N9O4++	527.62	38	12	0.4	14

Compound Number	#H-bond acceptors	#H-bond donors	MR	TPSA	iLOGP	XLOGP3
**2**	5	6	110.08	224.82	0.25	-3.59
**1**	6	5	107	221.86	0.66	-5.22
**21**	6	7	120.95	231.06	0.63	-3.87
**18**	5	5	119.88	202.04	1.6	-3.01
**15**	5	6	114.98	210.83	0.83	-3.19
**16**	5	6	119.79	210.83	1.33	-2.82
**19**	5	6	122.76	210.83	1.22	-3.08
**17**	5	6	124.6	210.83	1.37	-2.3
**20**	5	6	127.57	210.83	1.53	-2.61
**22**	5	6	144.28	210.83	1.65	-1.23

Compound Number	WLOGP	MLOGP	Silicos-IT Log P	Consensus Log P	ESOL Log S	ESOL Solubility (mg/mL)
**2**	-5.67	-5.92	-3.58	-3.7	0.31	8.61E+02
**1**	-6.41	-5.52	-3.34	-3.97	1.34	9.16E+03
**21**	-6.05	-6.2	-3.3	-3.76	0.42	1.24E+03
**18**	-5.07	-5.45	-3.16	-3.02	-0.16	3.15E+02

**15**	-5.41	-5.69	-3.12	-3.31	0.04	4.80E+02
**16**	-5.02	-5.45	-2.71	-2.94	-0.21	2.79E+02
**19**	-5.33	-5.3	-2.48	-2.99	-0.1	3.64E+02
**17**	-4.63	-5.23	-2.31	-2.62	-0.55	1.30E+02
**20**	-4.94	-5.08	-2.07	-2.63	-0.42	1.82E+02
**22**	-3.8	-4.37	-1.24	-1.8	-1.65	1.19E+01

**Compound Number**	**ESOL Solubility (mol/l)**	**ESOL Class**	**Ali Log S**	**Ali Solubility (mg/mL)**	**Ali Solubility (mol/L)**	**Silicos-IT LogSw**
**2**	2.03E+00	Highly soluble	-0.55	1.20E+02	2.84E-01	-0.2
**1**	2.16E+01	Highly soluble	1.21	6.81E+03	1.61E+01	0.02
**21**	2.65E+00	Highly soluble	-0.39	1.92E+02	4.10E-01	-0.8
**18**	6.98E-01	Very soluble	-0.67	9.64E+01	2.14E-01	-0.64
**15**	1.10E+00	Highly soluble	-0.67	9.39E+01	2.15E-01	-0.98
**16**	6.17E-01	Very soluble	-1.05	4.00E+01	8.87E-02	-1.38
**19**	7.88E-01	Very soluble	-0.78	7.62E+01	1.65E-01	-1.06
**17**	2.79E-01	Very soluble	-1.59	1.19E+01	2.56E-02	-1.77
**20**	3.83E-01	Very soluble	-1.27	2.55E+01	5.37E-02	-1.45
**22**	2.26E-02	Very soluble	-2.7	1.05E+00	1.98E-03	-3.83

**Compound Number**	**Silicos-IT Solubility (mg/mL)**	**Silicos-IT Solubility (mol/L)**	**Average Activity against *S. aureus* (ug/mL)**	**Average Activity against MRSA (ug/mL)**	**Average Activity against *E. faecalis* (ug/mL)**	**Average Activity against VRE (ug/mL)**
**2**	2.67E+02	6.31E-01	32	32	16	64
**1**	4.40E+02	1.04E+00	>256	256	64	256
**21**	7.38E+01	1.58E-01	64	48	32	128
**18**	1.03E+02	2.28E-01	64	32	32	96
**15**	4.53E+01	1.04E-01	32	48	32	32
**16**	1.90E+01	4.22E-02	32	32	16	64
**19**	4.01E+01	8.69E-02	32	24	16	64
**17**	7.98E+00	1.71E-02	24	24	16	64
**20**	1.68E+01	3.54E-02	16	32	16	64
**22**	7.88E-02	1.49E-04	12	12	8	32

References

(1) Davison, J. R.; Lohith, K. M.; Wang, X.; Bobyk, K.; Mandadapu, S. R.; Lee, S.-L.; Cencic, R.; Nelson, J.; Simpkins, S.; Frank, K. M.; et al. A new natural product analog of blasticidin S reveals cellular uptake facilitated by the NorA multidrug transporter. *Antimicrob. Agents Chemother.* **2017**, *61* (6).

(2) Svidritskiy, E.; Korostelev, A. A. Mechanism of inhibition of translation termination by blasticidin S. *J. Mol. Biol.* **2018**, *430* (5), 591-593.

(3) Morris, G. M.; Huey, R.; Lindstrom, W.; Sanner, M. F.; Belew, R. K.; Goodsell, D. S.; Olson, A. J. AutoDock4 and AutoDockTools4: Automated docking with selective receptor flexibility. *J. Comput. Chem.* **2009**, *30* (16), 2785-2791.

(4) Trott, O.; Olson, A. J. AutoDock Vina: improving the speed and accuracy of docking with a new scoring function, efficient optimization, and multithreading. *J. Comput. Chem.* **2010**, *31* (2), 455-461.

(5) Breiner, L. M.; Briganti, A. J.; McCord, J. P.; Heifetz, M. E.; Philbrook, S. Y.; Slebodnick, C.; Brown, A. M.; Lowell, A. N. Synthesis, testing, and computational modeling of pleuromutilin 1,2,3-triazole derivatives in the ribosome. *Tetrahedron Chem* **2022**, *4*, 100034.

(6) Svidritskiy, E.; Ling, C.; Ermolenko, D. N.; Korostelev, A. A. Blasticidin S inhibits translation by trapping deformed tRNA on the ribosome. *Proc. Natl. Acad. Sci. U.S.A.* **2013**, *110* (30), 12283-12288.

(7) Daina, A.; Michielin, O.; Zoete, V. SwissADME: A free web tool to evaluate pharmacokinetics, drug-likeness and medicinal chemistry friendliness of small molecules. *Sci. Rep.* **2017**, *7* (1), 42717.
